# Controlling Redox and Photophysical Properties of First-Row Transition Metal Complexes via Ligand Perhalogenation

**DOI:** 10.1021/acs.inorgchem.5c05333

**Published:** 2026-02-28

**Authors:** Tim-Niclas Streit, Malte Sellin, Bruno Lazarevski, Oliver S. Wenger, Moritz Malischewski

**Affiliations:** † Freie Universität Berlin, Institut für Chemie und Biochemie, Fabeckstraße 34-36, 14195 Berlin, Germany; ‡ University of Basel, Department of Chemistry, St. Johanns-Ring 19, 4056 Basel, Switzerland

## Abstract

Halogenation of ligands intensely modulates the redox and photophysical properties of transition-metal complexes, yet fully halogenated systems remain largely unexplored. Here we report the synthesis and structural characterization of homoleptic Ni(0) complexes with perhalogenated aryl isocyanide ligands [Ni­(CN-C_6_X_5_)_4_] (X = F, Cl). Comparative electrochemical studies reveal a dramatic anodic shift of the Ni(0)/Ni­(I) couple from −0.60 V in [Ni­(CN–C_6_H_5_)_4_] to +0.03 V vs Fc^+/0^ for the perfluorinated species, reflecting the exceptional π-acceptor strength resulting from the C–H/C–F persubstitution. Surprisingly, metal-to-ligand charge-transfer (MLCT) absorption energies remain largely unchanged, a result supported by DFT calculations showing concurrent stabilization of both the Ni-centered HOMO and ligand-based LUMO. In contrast, the perchlorinated complex exhibits a red-shifted MLCT band due to asymmetric frontier-orbital tuning. Ultrafast transient absorption spectroscopy demonstrates ^3^MLCT excited states with lifetimes in the regime of 66–141 ps for all complexes. These findings establish perhalogenated isocyanides as powerful ligands for controlling excited-state redox potentials without altering excitation energies, an attractive feature for the rational design of robust Ni-based photoredox catalysts. More broadly, our findings establish ligand perhalogenation as a design strategy for developing new photoactive first-row transition metal complexes with potential applications in luminescent devices, photocatalysis, and photodynamic therapy.

## Graphical abstract

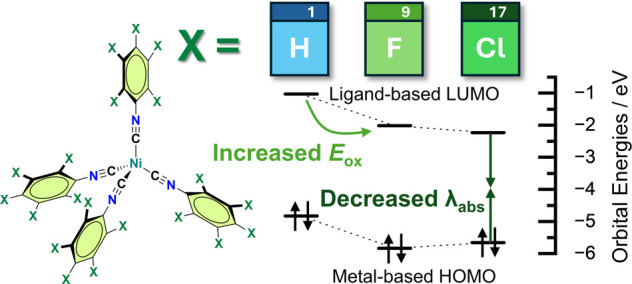

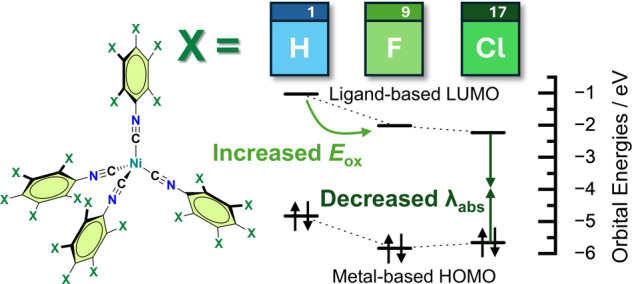

## Introduction

Photoactive complexes find many applications, including in the fields of catalysis,^1,2^ light-emitting devices,^3,4^ bioimaging and solar energy conversion.^5–7^ Almost all photoactive transition-metal complexes incorporate aryl moieties in the ligands, often to enable charge-transfer excited states; among these, triplet metal-to-ligand charge transfer (^3^MLCT) states have been historically dominant, although other charge-transfer and metal-centered excited states have also proven useful, particularly in the past decade.^8–10^ Targeting highly oxidative excited states, electron-withdrawing groups such as –F or –CF_3_ can be introduced into the aryl rings, lowering the σ-donor ability of the ligand. For the oxidative side of the photocatalysis, partially fluorinated iridium­(III) complexes are dominating.^11–13^ The introduction of trifluoromethyl groups at the cyclometalated phenyl rings lowers the σ-donor ability and thus increases the excited state reduction potential (*E*_1/2_(Ir^(IV/III)^)) of the complex.^14^ A handful of these Ir­(III) catalysts are even commercially available^15^ the most important being [Ir­({dF­(CF_3_)­ppy}_2_(dtbpy)]^+^[PF_6_]^−^ (Figure 1A), with an excited state potential of +0.92 V vs Fc^+/0^, more than half a volt stronger than its nonfluorinated version.^16^ Although there are some examples of the incorporation of fluoro and trifluoromethyl groups in ruthenium-based catalysts yielding similar improvements, these complexes are less established.^17,18^

**Figure 1:**
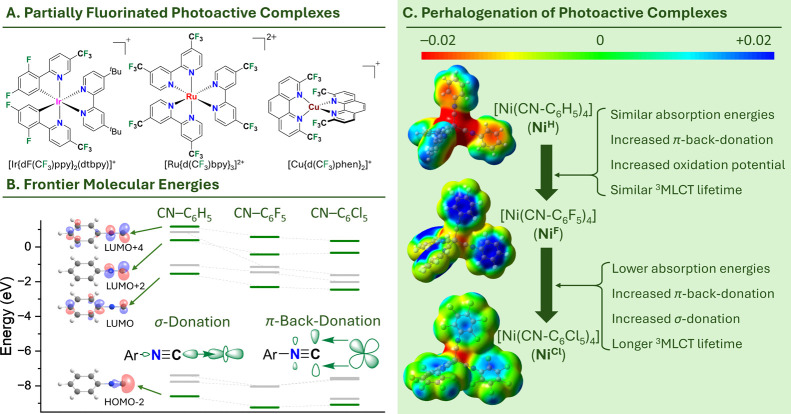
(A) Selection of partially fluorinated photoactive complexes. (B) Comparison of the frontier molecular orbital energies of phenyl isocyanide (CN–C_6_H_5_) with its perfluorinated (CN–C_6_F_5_) and its perchlorinated (CN–C_6_Cl_5_) version and the Dewar-Chatt-Duncanson model highlighting the relevant σ-donor and π-acceptor orbitals (C) Electrostatic surface potentials (ESPs; calculated at the B3LYP/def2-TZVP level of theory, 0.02 e^–^ Bohr^–3^) of **Ni** ^**H**^, **Ni** ^**F**^, and **Ni** ^**Cl**^, highlighting the polarity inversion of the C–X bonds.

As precious metals are a considerable cost factor for large-scale photophysical and photochemical applications, purely organic molecules and complexes featuring more earth-abundant first-row transition metals are getting increasing attention.^19–23^ Also here, fluorinated groups have been used for tailoring ligand properties.^24–27^ As metal-centered (MC) excited states are an especially prominent nonradiative decay pathway for ^3^MLCT excited states of first-row transition metal complexes,^10,28^ otherwise commonly used ligands such as bipyridine (bpy) yield long-lived ^3^MLCT states mostly in *d* ^10^ systems.^29–31^ Therefore, a very active field in photocatalysis has evolved around copper­(I), having excellent lifetimes and high luminescence quantum yields.^29,30,32^ Despite its identical electron configuration, ^3^MLCT states in nickel(0) complexes have received so far near to no attention.^33^ While formally isoelectronic Ni­(bpy)_2_ complexes are known, they are very reductive species,^34,35^ as they are best formulated as Ni^I^(bpy^.–^)­(bpy^0^), featuring a *d* ^9^ electron count.^36^

Nickel­(0) complexes with reasonable stability can be best achieved by using π-acceptor ligands (Dewar-Chatt-Duncanson model, Figure 1B),^37^ withdrawing excess electron density away from the metal atom, stabilizing the otherwise high-lying filled 3*d* orbitals. Aryl isocyanide ligands are good π-acceptor ligands, which have already been used to form well-performing photoactive complexes along with W^0^, Mo^0^, Cr^0^, and Mn^I^ metal centers.^38–41^ The perfluorination of the aryl moiety decreases the energies of both the σ-donor and π-acceptor orbitals by ca. 0.6 eV (Figure 1B), further increasing the π-acceptor ability of the ligand. Besides some examples of partially fluorinated aryl isocyanide ligands,^42–44^ the only example of a successfully isolated perfluorinated aryl isocyanide we found is pentafluorophenyl isocyanide (CN–C_6_F_5_).^45,46^ While a few transition-metal complexes containing CN–C_6_F_5_ have been prepared in seminal works by Lentz and co-workers, none of these complexes have been photophysically explored to date.^47^

Exchanging fluorine for chlorine in the phenyl isocyanide framework, the relevant orbital energies suggest that CN–C_6_Cl_5_ should be both an even better σ-donor and π-acceptor than CN–C_6_F_5_ (Figure 1B). Still, it seems that the coordination chemistry of the CN–C_6_Cl_5_ is even less explored than that of CN–C_6_F_5_, likely due to the fact that its synthesis involves phosgene.^48–51^ Photoactive complexes incorporating perchlorinated ligands, in general, have received little attention until now. One of only a few exceptions is octachlorophenanthroline (phen^Cl^).^52,53^ The MLCT band of its homoleptic copper complex [Cu­(phen^Cl^)_2_]^+^ is shifted by ca. 0.24 eV relative to its nonhalogenated analogue, which has been attributed to the stabilization of the π*-orbitals through the chlorination.^52^

The primary goal of this study is to evaluate the potential of ligand perfluorination and perchlorination as molecular design strategies for enhancing the photophysical and photochemical properties of transition metal compounds, with particular focus on first-row *d*-block elements. In pursuit of this greater objective, the photophysical and electrochemical properties of [Ni­(CN–C_6_X_5_)_4_] (X = H, F, Cl; **Ni** ^**X**^) complexes are examined using steady-state and fs-transient UV–vis absorption spectroscopy, cyclic voltammetry, as well as IR-, and Raman-spectroscopy. The study reveals key distinctions between perfluorination and perchlorination, leading to new design principles for next-generation luminophores and photocatalysts through strategies that have remained largely unexplored until now.^28^

## Results and Discussion

### Synthesis

Isocyanides are typically prepared by the *N*-formylation with subsequent dehydration (Scheme 1).^54,55^ This method is, however, not suitable for very electron-deficient organic isocyanides, as it leads to very low yields or uses very toxic dehydration agents.^50,51^ An early successful preparation of pure CN–C_6_F_5_ was developed by Lentz *et al.*, in which the respective *N*-dibromomethanimine is reduced by magnesium to the analytically pure desired product.^45^ In analogy to that procedure, we synthesized the perchlorinated isocyanide CN–C_6_Cl_5_ by the reduction of its known *N*-dichloromethanimine derivative using Co­(C_5_H_5_)_2_. While neat CN–C_6_F_5_ is unstable above its melting point at 13 °C, CN–C_6_Cl_5_ is a stable white solid, which can even be sublimed at reduced pressure.

**Scheme 1:**
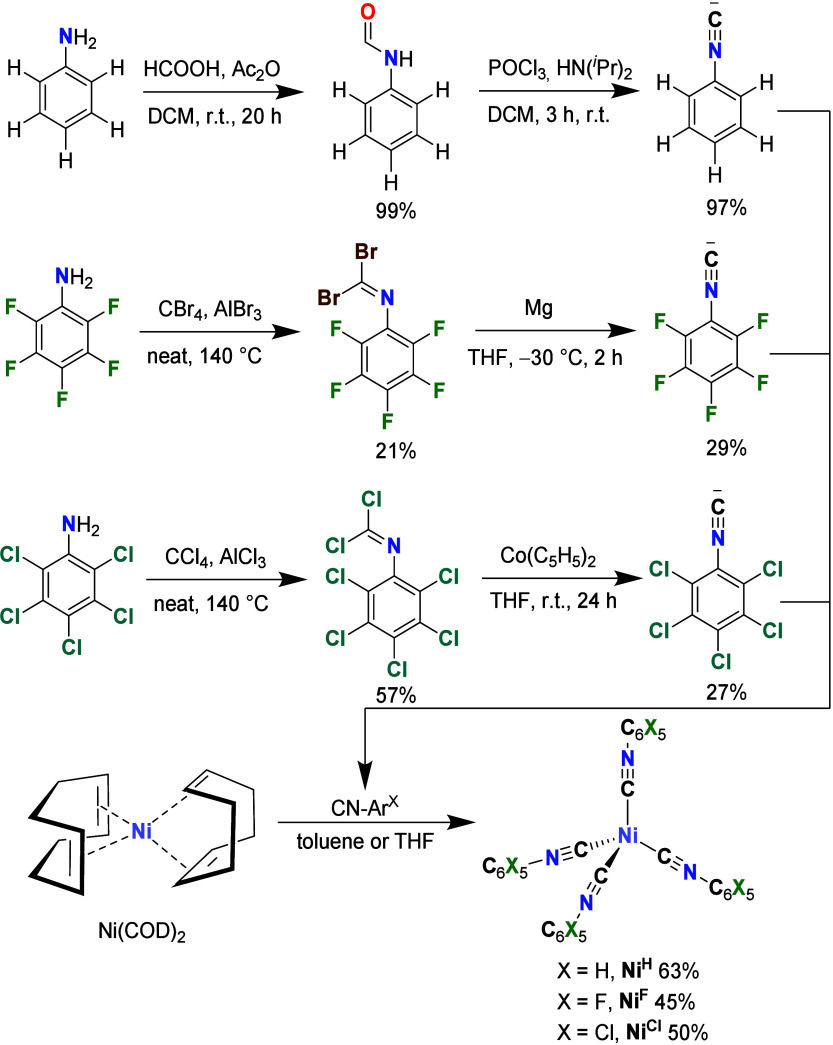
Synthesis of the Phenyl Isocyanide in Comparison to the Synthesis of Its Perfluorinated and -Chlorinated Derivatives and the Synthesis of the **Ni** ^**X**^ Complexes from Ni­(COD)_2_ ^a^

a COD = 1,5-cyclooctadiene.

The synthesis of the **Ni** ^**X**^ complexes was done in analogy to the known procedure for **Ni** ^**H**^ by Hahn et al.^56^ through the combination of Ni­(COD)_2_ (COD = 1,5-cyclooctadiene) with an excess of the respective isocyanide in tetrahydrofuran (THF) or toluene. This produced in all three cases the respective complexes in moderate yields from 45 to 63% (Scheme 1). During the synthesis of the **Ni** ^**X**^ complexes, their different solubilities became evident. While **Ni** ^**H**^ can be precipitated from solution by the addition of *n*-pentane to its solution in toluene, **Ni** ^**F**^ is even soluble in pure alkanes. Therefore, its isolation was done by removing the solvent under vacuum. In contrast, **Ni** ^**Cl**^ immediately precipitated from its THF solution as a brick-red solid.

### Characterization

Crystals of **Ni** ^**F**^ suitable for single-crystal X-ray diffraction (scXRD) could be obtained by slow cooling of a 1,2-difluorobenzene (*o*DFB) solution layered with *n*-pentane. The crystallization of **Ni** ^**Cl**^ was done by slow cooling of a boiling CCl_4_ solution to room temperature. **Ni** ^**F**^ and **Ni** ^**Cl**^ appear to be the first homoleptic perhalogenated aryl isocyanide complexes to be structurally characterized.

Comparison of the known solid-state structure of **Ni** ^**H**^ ^56^ with its perhalogenated analogues, **Ni** ^**F**^ and **Ni** ^**Cl**^ did not show any significant differences in the Ni–C and C≡N distances nor the Ni–C≡N angles (see Supporting Information Table S2). The direct coordination environment of the nickel atom [NiC_4_] is almost tetrahedral in all cases, with only a few degrees of deviation from the ideal tetrahedral angle of 109.5°. An attractive halogen–halogen interaction between two chlorine atoms in the *ortho*-positions in the aryl moieties **Ni** ^**Cl**^ lead to a slight compression of two C–Ni–C angles to 102.9°. The Hirshfeld surface analysis^57,58^ shows a trend of increasing directional interactions from **Ni** ^**H**^ over **Ni** ^**F**^ to **Ni** ^**Cl**^ (Figure 2), similar to the corresponding benzene derivatives: while the fingerprint plot of **Ni** ^**H**^ only shows irregular, mostly Van-der-Waals (VdW) driven interactions, including weak H···H interactions and minor C–H···π contacts, the interactions become more defined in the case of **Ni** ^**F**^, showing weak, likely dispersion driven F···F interactions and C–F···π* contacts.^59,60^ In the case of **Ni** ^**Cl**^, the intermolecular interactions become very evident: offset π-π stacking leads to well-defined C···C and Cl···C distances below the sum of the VdW radii. Additionally, moderate σ-holes are leading to some moderately strong Cl···Cl interactions. Through the similar VdW radii of carbon and chlorine, these features overlap in the Hirshfeld fingerprint plot. The element filtered Hirshfeld surfaces are discussed in the Supporting Information section 5.

**Figure 2:**
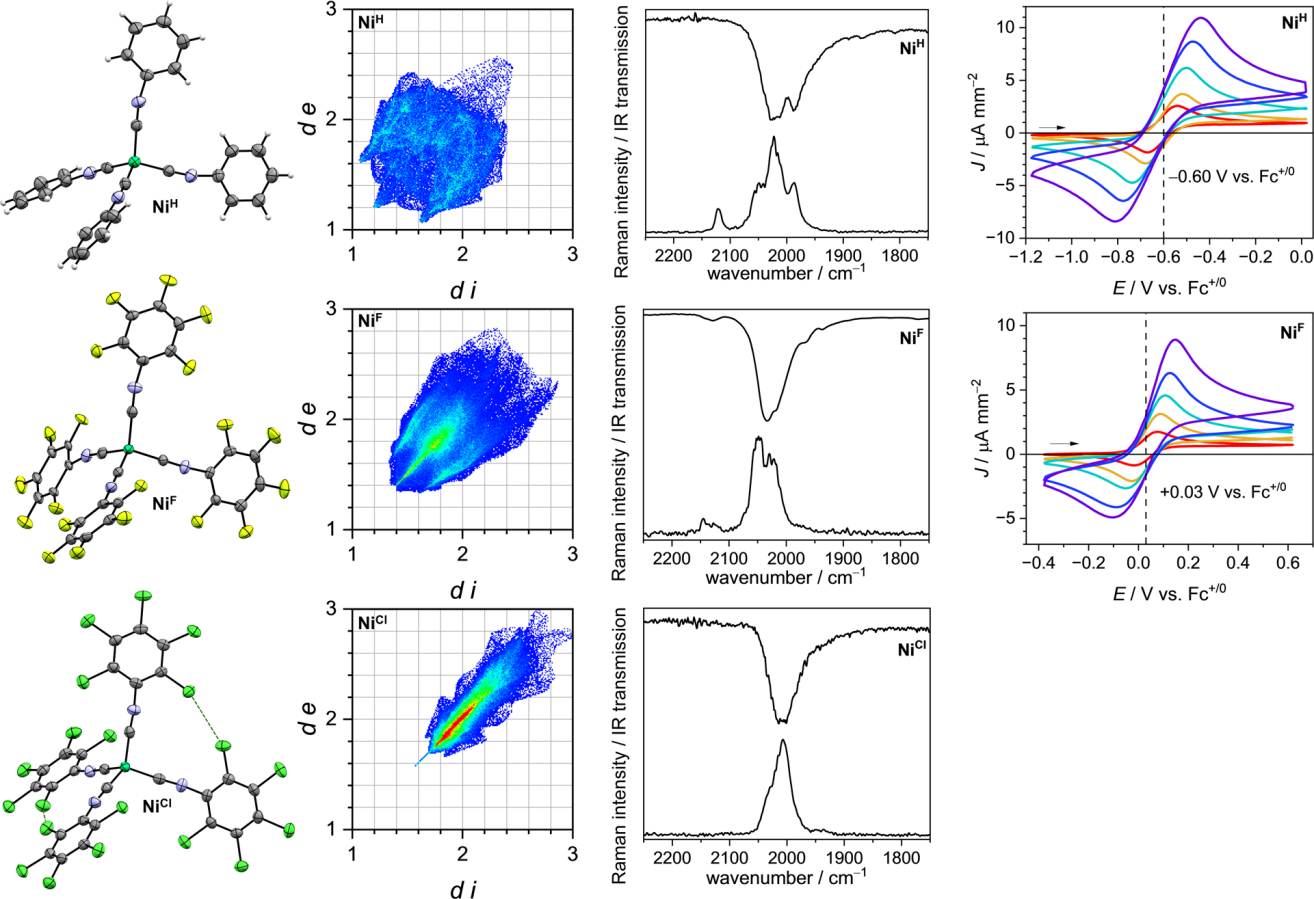
Left: molecular structures of **Ni** ^**H**^ (top, taken from Hahn et al.^56^ CSD number 234058), **Ni** ^**F**^ (middle), and **Ni** ^**Cl**^ (bottom) determined by single-crystal X-ray diffraction. Thermal displacement ellipsoids set at 50% probability. Color code: nickel – turquoise, chlorine – dark green, fluorine – light green, nitrogen – light blue, carbon – dark gray, hydrogen – light gray. Middle-left: corresponding Hirshfeld surface fingerprints of the **Ni** ^**X**^ complexes. *d e* = external core – surface distance; *d i* = internal core – Hirshfeld surface distance. Increasing number of points increases from blue over green and yellow to red. Middle right: C≡N stretching region of the vibrational spectra of the **Ni** ^**X**^ complexes composed of the ATR-IR spectra (top) and the Raman spectra (bottom). Right: Cyclic voltammograms (CVs) of **Ni** ^**H**^ and **Ni** ^**F**^ at scan rates between 20 mV s^–1^ (red) and 1000 mV s^–1^ (purple) in a 10 mM *o*DFB solution. Electrochemical data of **Ni** ^**Cl**^ could not be obtained due to its insufficient solubility.

The π-back-donation can be traced using the vibrational spectra of the compounds. All three **Ni** ^**X**^ complexes feature a band between 2050–2000 cm^–1^ from the ν­(C≡N) stretching vibration in the vibrational spectra, which corresponds to a red-shift of ca. 100 cm^–1^ relative to the free isocyanide ligands (Table 1). Baseline distortions of strong ATR-IR bands, attributed to anomalous dispersion effects, were minimized by measuring a Nujol dispersion for the case of **Ni** ^**H**^ and **Ni** ^**Cl**^. However, the bands attributed to the ν­(C≡N) stretching vibrations remained broad. The ν­(C≡N) stretching bands are similarly broad in the Raman spectra of the three complexes. For **Ni** ^**H**^ and **Ni** ^**F**^, additionally, the *A*_1_ symmetric stretching vibrations are found at 2122 and 2147 cm^–1^, respectively. The band positions are in good agreement with DFT calculations and match the expected patterns for the *pseudo*-tetrahedral {Ni­(CN)_4_} core.

**Table 1:** Observed ν­(C≡N) Stretching Vibrations in the IR- and Raman-Spectra in Comparison with the DFT-Calculated Values (B3LYP­(D3BJ)/def2-TZVP Level of Theory) Scaled by 0.967 (*in Italics*)­^a^

isocyanide	free ligand (cm^–1^)	NiL_4_ complex (cm^–1^)
CN–C_6_H_5_	2125 (IR)^a^	2008 (IR)/2122, 2023 (Raman)
	*2134 (A_1_)*	*2144 (A_1_), 2052 (T_2_)*
CN–C_6_F_5_	2131 (IR)^b^	2127, 2033 (IR)/2147, 2049 (Raman)
	*2133 (A_1_)*	*2150 (A_1_), 2055 (T_2_)*
CN–C_6_Cl_5_	2115 (IR)/2124 (Raman)	2028 (IR)/2007 (Raman)
	*2127 (A_1_)*	*2144 (A_1_), 2038 (T_2_)*

a The Mulliken symbols are given for the approximated *T*_d_ symmetry of the {Ni­(CN)_4_} core, although the complexes have a lower overall symmetry.

b From ref 61.

c From ref 45.

### Electrochemistry

Upon perfluorination of aromatic systems, typically a substantial increase in the ionization energy (IE) is observed. For the case of the perfluorination of benzene, this amounts to 0.66 eV.^62,63^ In contrast, the perchlorination of benzene does not alter its IE.^64^ However, in the condensed phase, the oxidation potentials (*E*_ox_) of perchlorinated arenes are only slightly smaller than the ones of perfluorinated arenes, while nonhalogenated arenes have significantly lower potentials.^65,66^

As the HOMO of the **Ni** ^**X**^ complexes is expected to be located essentially on the metal in all three investigated cases, any change of the oxidation potential is most likely indirect, transmitted over the altered ligand properties of the phenyl isocyanide ligand. As the perhalogenation positively affects the π-acceptor properties, it is expected that the *d*-orbitals at the metal atom are further stabilized, and a higher oxidation potential is observed.

In cyclic voltammetry (CV) measurements of **Ni** ^**H**^ in *o*DFB using [NBu_4_]^+^[PF_6_]^−^ as supporting electrolyte, the electrochemically quasi-reversible oxidation [**Ni** ^**H**^]^+/0^ is observed at −0.60 V vs Fc^+/0^. Performing CV measurements with **Ni** ^**F**^ under similar conditions yields an oxidation peak only (without corresponding reduction back-reaction) at ca. 0 V vs Fc^+/0^, indicating an EC (electrochemical-chemical) process. Exchanging the anion in the supporting electrolyte from [PF_6_]^−^ to [Al­(OC­(CF_3_)_3_)_4_]^−^ yields an electrochemically quasi-reversible potential at +0.03 V vs Fc^+/0^, suggesting that the electrochemically generated [**Ni** ^**F**^]^+•^ reacted with the [PF_6_]^−^ anion. Comparing the two potentials shows an increase of the half-wave potential by 0.63 V. This is a similar difference to the increase of the IE upon the perfluorination of benzene; still, the oxidation is attributed to a metal-based orbital. Therefore, comparing the oxidation potentials of the **Ni** ^**X**^ complexes gives a quantification of the net-donor ((σ-donor) – (π-acceptor)) properties of the ligands, showing the stronger relative backdonation properties of the perfluorinated isocyanide ligand. When comparing these values to the oxidation potential of [Ni­(CO)_4_]^+•/0^ with +1.21 V vs Fc^+/0^ it becomes evident that the isocyanides, irrespective of whether they are perfluorinated or not, are still better net-donor ligands than carbonyls.^67^ For reference, in Ni^0^ complexes with chelating aryl isocyanide ligands, the respective potential is – 0.3 V vs Fc^+/0^,^68^ whereas in Ni­(PPh_3_)_4_ and Ni­(PEt_3_)_4_ this oxidation process occurs at −1.0 V and −1.35 V vs Fc^+/0^, respectively.^69^

A similar increase in the oxidation potential would be expected for the perchlorination to **Ni** ^**Cl**^ based on the orbital energies of the CN–C_6_Cl_5_ ligand. Indeed, one of the few studies on CN–C_6_Cl_5_ complexes has validated this effect on a heteroleptic manganese complex.^50^ In the case of the homoleptic nickel complexes, however, no electrochemical data of **Ni** ^**Cl**^ could be gathered due to the very low solubility of the complex (≪1 mM).

### Bond Analysis

As the finer differences in the binding properties of the different ligands in the **Ni** ^**X**^ complexes were not evident from the molecular structures and vibrational spectra, we additionally compared them to the respective Cr­(CO)_5_ complexes, as the chromium pentacarbonyl fragment is an excellent benchmark system for estimating the binding properties of isocyanide ligands.^42,70^ Both the parent phenyl isocyanide complex (OC)_5_Cr­(CN–C_6_H_5_) and its perfluorinated derivative (OC)_5_Cr­(CN–C_6_F_5_) have been previously synthesized and computationally analyzed. We synthesized (OC)_5_Cr­(CN–C_6_Cl_5_) by the combination of (OC)_5_Cr­(THF) with an excess of CN–C_6_Cl_5_. In comparison to all other phenyl isocyanide complex derivatives, Cr­(CO)_5_(CN–C_6_Cl_5_) features the shortest Cr–C distance (1.937(4) Å) and the largest deviation of the CNC angle (169.8(4)°) from linearity, even exceeding the values for the previous record-holder Cr­(CO)_5_(CN–C_6_F_5_) (1.945(4) Å; 173.6(4)°).^70^ This indicates that CN–C_6_Cl_5_ is a superior π-acceptor to CN–C_6_F_5_, which is in accordance with the lower energies of the respective molecular orbitals (Figure 1B).

To computationally confirm this trend, we performed an energy decomposition analysis (EDA-NOCV) of (OC)_5_Cr interacting with the CN–C_6_X_5_ (X = H, F, Cl) ligands. While it was already previously reported that CN–C_6_F_5_ is a superior π-acceptor to CN–C_6_H_5_, the σ-donor properties remain similar in the case of Cr­(CO)_5_,^70^ here, we additionally performed calculations on the same level of theory (BP86­(D3BJ)/def2-TZVPP//BP86­(D3BJ)/TZ2P) for CN–C_6_Cl_5_. Interestingly, the perchlorinated phenyl isocyanide is both a superior π-acceptor and σ-donor to both the perfluorinated and nonfluorinated phenyl isocyanide, yielding an increase of the total (Δ*E*_int_) and orbital interaction energies (Δ*E*_orb_) along the series CN–C_6_H_5_, CN–C_6_F_5_, CN–C_6_Cl_5_ (see SI Table S3).

Performing the EDA-NOCV analysis for the **Ni** ^**X**^ complexes yields the same trend in the Δ*E*_int_ and Δ*E*_orb_ values. Furthermore, we calculated the Gibbs complexation energies (Δ_rxn_ *G*°, B3LYP­(D3BJ)/def2-TZVPP level of theory) in the gas-phase between the Ni atoms with the ligands forming the **Ni** ^**X**^ complexes. This reaction is 35 kJ mol^–1^ more exergonic for the case of **Ni** ^**F**^, as in the case of **Ni** ^**H**^. The exchange from **Ni** ^**F**^ to **Ni** ^**Cl**^, shows even a slight increase of the coordination ability of by 4 kJ mol^–1^ (Table 2).

**Table 2:** Gibbs Complexation Energies Ni­(T) + 4xL­(S) → NiL_4_(S) Calculated on the B3LYP­(D3BJ)/def2-TZVPP Level of Theory and Results of the EDA-NOCV Calculations Ni­(S)+4xL­(S) → NiL_4_(S) on the BP86­(D3BJ)/def2-TZVP//BP86­(D3BJ)/TZ2P Level of Theory^a^

NiL_4_	Δ_rxn_ *G*°	Δ*E*_int_	Δ*E*_Pauli_	Δ*E*_elstat_	Δ*E*_disp_	Δ*E*_orb_
Ni^H^	–385	–1225	4544	–2097	–22	–3650
Ni^F^	–420	–1232	4693	–2154	–22	–3749
Ni^Cl^	–424	–1243	4687	–2145	–27	–3758
Ni(CO)_4_	–374	–932	2594	–1715	–16	–1794

a All energies are given in kJ mol^–^^1^.

### Optical Absorption Spectroscopy

The UV–vis absorption spectra of the **Ni** ^**X**^ complexes have a similar overall structure to the copper­(I) diimine complexes, consisting of intense π-π* transitions of the ligand in the UV–B/C regime and MLCT transitions in the UV-A/blue regime. The parent **Ni** ^**H**^ complex features an MLCT absorption band at 375 nm. The absorption band maximum of the **Ni** ^**F**^ complex is at a similar wavelength (368 nm), but the **Ni** ^**Cl**^ complex is bathochromically shifted to 421 nm (Figure 3).

DFT calculations indicate that the lowest energy transitions have strong ^1^MLCT character in all three complexes (B3LYP­(D3BJ)/def2-TZVP level of theory). Furthermore, the experimental UV–vis absorption spectra are in excellent agreement with their calculated DFT electronic transitions and oscillator strengths, including the pronounced red-shift of the MLCT absorption band in **Ni** ^**Cl**^ with respect to **Ni** ^**H**^ and **Ni** ^**F**^.

**Figure 3:**
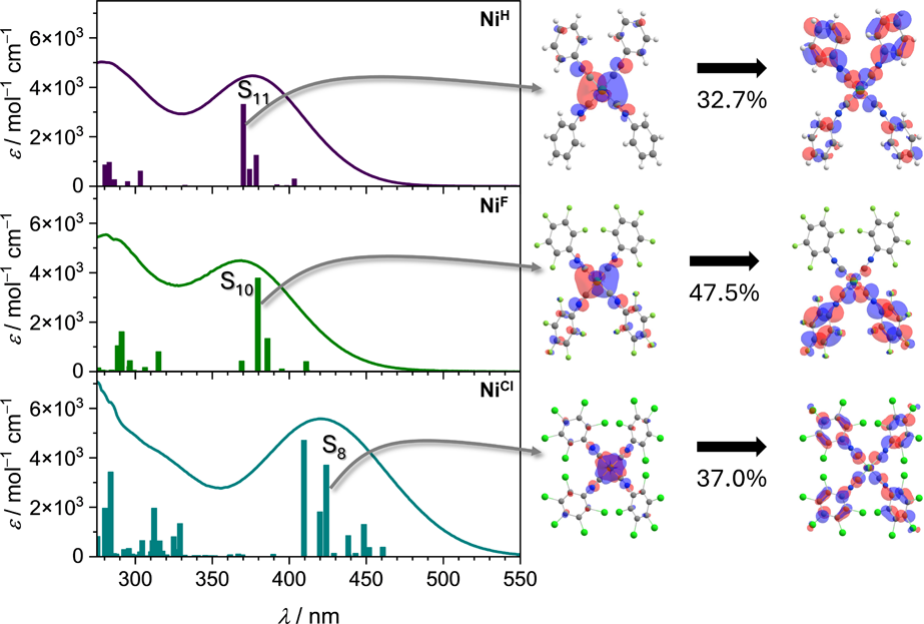
Left: experimental UV–vis absorption spectra of **Ni** ^**H**^ (top), **Ni** ^**F**^ (middle), and **Ni** ^**Cl**^ (bottom) in THF (solid lines) compared with calculated oscillator strengths (vertical bars) obtained at the B3LYP­(D3BJ)/def2-TZVP level of theory. Right: dominant hole–particle natural transition orbital (NTO) pair associated with a representative excitation. NTO phases are chosen arbitrarily; orbitals are visualized at an isodensity value of 0.03 *e* Bohr^–3^; B3LYP­(D3BJ)/def2-TZVP level of theory). Complete NTO analyses for singlet excited states S_1_–S_12_ including all contributions exceeding 2% of the total transition density, are provided in the Supporting Information in Tables S3–S5.

The difference in the spectra of **Ni** ^**H**^ and **Ni** ^**F**^ is surprisingly small, given the 0.63 V difference in the respective half-wave potentials of the Ni^+/0^ oxidation events. Comparing the molecular orbital diagrams of **Ni** ^**H**^ and **Ni** ^**F**^ shows that the HOMO–LUMO gap increases only by 0.02 eV upon the perfluorination. This is in accordance with the experimentally observed blue shift of the absorption band by 0.06 eV and the strong increase of the Ni^+/0^ oxidation potential. The strongly red-shifted band in the perchlorinated complex **Ni** ^**Cl**^ implies a decrease of the HOMO–LUMO gap, which can be traced down to a decrease of the LUMO energy and an increase of the HOMO energy, as seen from the MO-scheme. These two effects can be rationalized on the basis that the CN–C_6_Cl_5_ ligand is a better net-donor ligand in comparison to CN–C_6_F_5_, increasing the HOMO energy, and has lowered energies of its aryl π-system, decreasing the LUMO energy. The respective decrease of the Δ*E*(HOMO–LUMO) of **Ni** ^**Cl**^ relative to **Ni** ^**F**^ by 0.39 eV is in excellent accordance with the red-shift of the ^1^MLCT transition (0.42 eV) relative to **Ni** ^**F**^ (Figure 4, Table 3).

**Figure 4:**
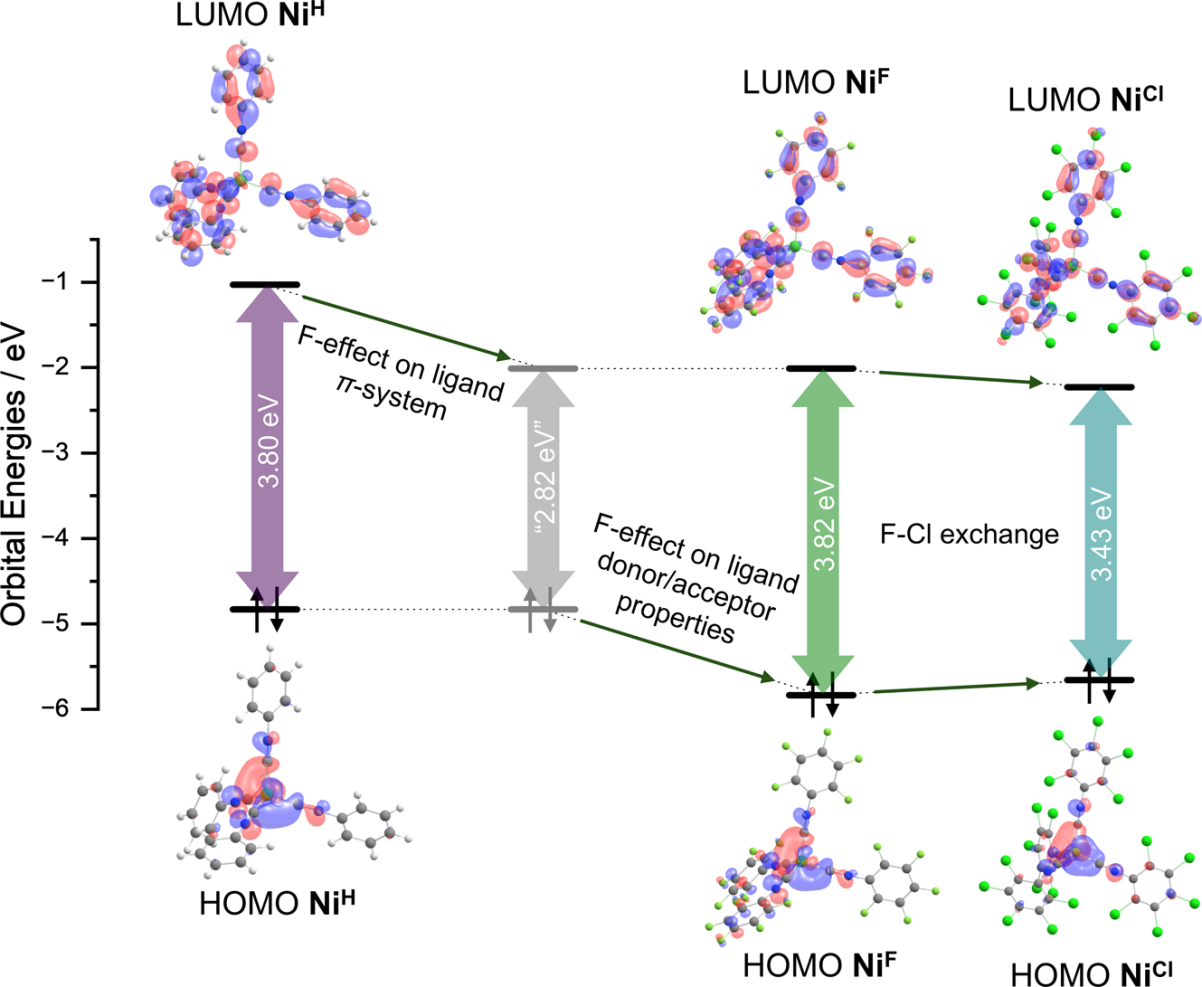
Calculated frontier molecular orbital energies of the NiX complexes, illustrating the parallel stabilization of both the HOMO and LUMO from **Ni** ^**H**^ to **Ni** ^**F**^, as well as the reduced HOMO–LUMO gap in **Ni** ^**Cl**^ relative to the other complexes. Molecular orbitals are visualized at an isodensity value of 0.03 *e* Bohr^–3^. Calculations were performed at the B3LYP­(D3BJ)/def2-TZVP level of theory.

**Table 3:** Key Experimental UV–Vis Absorption Spectroscopy Data in Comparison with the Calculated Wavelengths and Energies on the B3LYP­(D3BJ)/def2-TZVP Level of Theory (in *Italics*, FWHM = 30 nm), Calculated HOMO-LUMO Gaps, Experimental Excited State Lifetime of the ^3^MLCT Obtained through the Global Analysis from Transient Absorption Spectroscopy, and Half-Wave Potentials of the First Oxidation Event

	*ε*_max_ (nm/eV)	Δ*E* (HOMO–LUMO) (eV)	*τ*(^3^MLCT)/ps	*E*_1/2_(+/0) vs Fc^+/0^ (V)
**Ni** ^**H**^	375/3.31	3.80	99 ± 10	–0.60
	374/3.32			
**Ni** ^**F**^	368/3.37	3.82	66 ± 15	+0.03
	382/3.25			
**Ni** ^**Cl**^	421/2.95	3.43	141 ± 11	^a^
	418/2.97			

a No redox event observed by cyclic voltammetry due to the very low solubility of **Ni** ^**Cl**^.

To study the photophysical properties of the ^3^MLCT excited state that is anticipated to be populated after intersystem crossing from the initially excited ^1^MLCT states, we measured photoluminescence spectra of the three complexes. Unfortunately, no emission could be detected either at room temperature in THF solution or at 77 K in a 2-methyltetrahydrofuran glass. Evidently, nonradiative processes dominate the excited state relaxation under the present conditions. Based on our previous work on photoactive metal complexes bearing chelating aryl isocyanides,^38–40^ we anticipate that photoluminescence could be observed with perhalogenated derivatives of aryl isocyanide ligands. However, achieving this would require additional synthetic efforts that fall beyond the scope of the current study, which is focused on establishing perhalogenation as a design principle for photoactive metal complexes.

### Transient UV–Vis Absorption Spectroscopy

The absence of deactivation pathways via metal-centered excited states in complexes with a *d* ^10^ electron configuration usually facilitates long-lived charge transfer states in first-row transition-metal complexes. For four-coordinate tetrahedral complexes, the flattening distortion is, however, a fast deactivation pathway.^71^ An extreme case is [Cu­(phen)_2_]^+^ (phen = 1,10-phenanthroline), where the corresponding deactivation is so rapid that the intersystem crossing is not fast enough to even substantially populate the T_1_ (^3^MLCT) state, as the S_1_ (^1^MLCT) state depopulates to the ground-state (S_0_) in less than 2 ps.^72^ Already applying a little steric hindrance to flattening distortion using dmphen (dmphen = 2,9-dimethyl-1,10-phenanthroline) allows the population of the T_1_ state and an excited-state lifetime of 40 ns. Optimizing the substituent pattern yields lifetimes in the range of a few microseconds at room temperature.^29^ While these effects have been investigated in detail in the case of copper­(I), little is known about the excited state dynamics of isoelectronic nickel(0) complexes. NiL_4_ complexes with triaryl phosphine and -phosphite ligands exhibit microsecond lifetimes at room temperature in solution, which is likely explained through the restriction of the flattening distortion by the size of the P­(OAr)_3_ ligands.^33,73–75^ Two recently prepared homoleptic nickel(0) complexes containing bulky chelating isocyanide ligands are not luminescent at room temperature, but have luminescence lifetimes of several hundred nanoseconds at 77 K in frozen toluene.^68^

As all the herein investigated complexes do not exhibit any luminescence at room temperature in THF solution, we expected lifetimes below 1 ns under these conditions. Therefore, transient absorption spectroscopy was measured with a femtosecond-pulsed laser, allowing the collection of data with picosecond resolution. For all three complexes, a ground-state bleach (GSB) and an excited-state absorption (ESA) were observed (Figure 5), whereby the observation of an ESA band suggests that we are indeed directly monitoring a charge-transfer excited state. The data has been analyzed using global analysis (see Supporting Information). The longest component (τ­(^3^MLCT)) was assigned to the energetically lowest ^3^MLCT excited state. While the ^3^MLCT state has a lifetime of ca. 99 ps in **Ni** ^**H**^, it is shorter in the case **Ni** ^**F**^ with ca. 66 ps.^91^ Of this set of compounds, the perchlorinated complex **Ni** ^**Cl**^ features the longest ^3^MLCT lifetime of ca. 141 ps.

**Figure 5:**
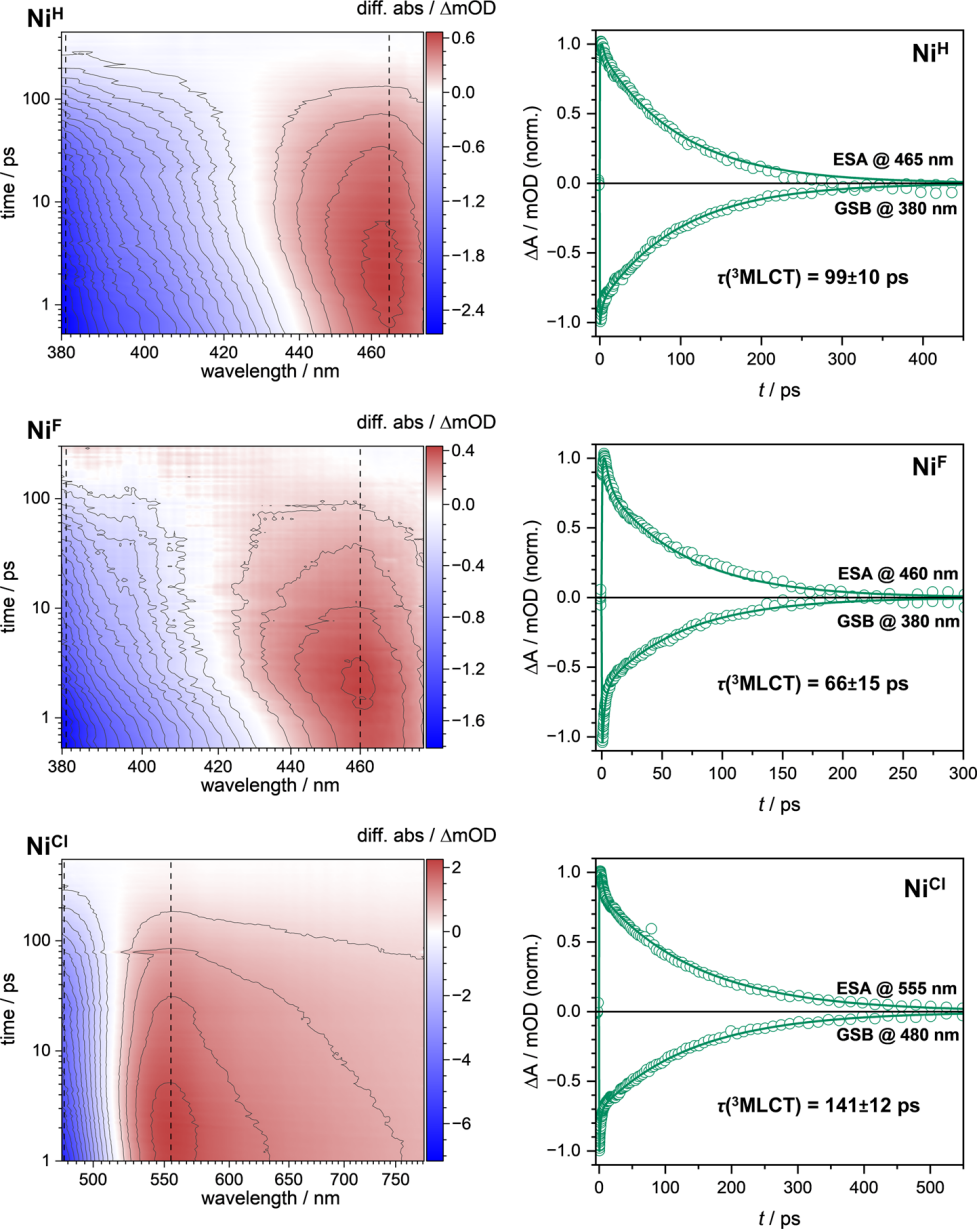
Left: femtosecond transient absorption spectra of the **Ni** ^**X**^ complexes. 2D plots show ΔA as a function of probe wavelength (nm) and pump–probe delay (ps). Negative (blue) and positive (red) signals correspond to GSB and ESA, respectively. Contour lines are drawn at constant ΔA intervals for both signs; the zero-line is omitted. Contour spacings: **Ni** ^**H**^ – 0.15 mOD, **Ni** ^**F**^ – 0.1 mOD, **Ni** ^**Cl**^ – 0.5 mOD. Right: normalized kinetic traces extracted at the wavelengths corresponding to the most positive ESA and the most negative GSB of the **Ni** ^**X**^ complexes (circles), as determined from the transient absorption spectra, in comparison with the global analyses (lines). The traces were normalized to their respective maximum absolute ΔA values to facilitate comparison of the dynamics. Measurement details: **Ni** ^**H**^ (top, exc. @370 nm; pulse energy – 0.18 μJ; OD@370 nm = 0.3; solvent – THF), **Ni** ^**F**^ (middle, exc. @360 nm; pulse energy – 0.16 μJ; OD@360 nm = 0.3; solvent – THF) and **Ni** ^**Cl**^ (bottom, exc. @390 nm; pulse energy – 0.22 μJ; OD@390 nm = 0.4; solvent – THF). UV–vis spectra before and after the measurements, logarithmic plots of the kinetic traces and all transient absorption spectra below 6 ps can be found in the Supporting Information section 6 in Figures S27–S41.

## Conclusion

The homoleptic nickel(0) complexes [Ni­(CN–C_6_F_5_)_4_] and [Ni­(CN–C_6_Cl_5_)_4_] represent rare examples of perhalogenated aryl isocyanide coordination chemistry. Relative to [Ni­(CN–C_6_H_5_)_4_], the perfluorinated complex exhibits an anodic shift of the Ni^0^/Ni^+^ couple by 0.6 V from −0.60 V to +0.03 V, reflecting the π-acceptor strength imparted by C–F substitution. The MLCT absorption energy nonetheless remains essentially unchanged, due to the parallel stabilization of the ligand-centered LUMO in addition to the metal-based HOMO. By contrast, the perchlorinated analogue displays a red-shifted MLCT band, consistent with asymmetric tuning of frontier orbital energies. This finding introduces a conceptually new approach to tuning the photophysical properties of first-row transition metal complexes. The ability to tune HOMO and LUMO energies is crucial for controlling luminescence color, photoredox properties, and excited-state energies.^6,76^ However, achieving this level of control has been significantly more challenging in first-row transition metal complexes compared to photoactive compounds based on precious metals.^28^

Our study establishes perfluorinated and perchlorinated aryl isocyanides as powerful, oxidation-stabilizing ligands that enable fine control over excited-state redox potentials, an attractive feature for photocatalysis, light-mediated cross-coupling, and photophysical devices where other systems currently dominate. The inherent oxidative robustness of the C–F motif could extend the operational window of Ni-based photosensitizers under strongly oxidizing or protic conditions where C–H analogues often degrade.

Looking ahead with nickel(0), the development of chelating or polydentate perfluorinated isocyanide ligands appears promising for enhancing both complex stability and photophysical properties. More broadly, the concept of perhalogenation could be extended to a wide range of ligand classes suitable for coordinating various earth-abundant transition metals, opening new perspectives in photophysics and photochemistry beyond conventional approaches.

## Experimental and Computational Methods

### General Conditions

All reactions and workups were performed in previously heated glassware under an atmosphere of argon using standard Schlenk techniques^77^ and an oil pump vacuum of 10^–3^ mbar. Room temperature (r.t.) refers to 25 °C. The addition of liquid reagents and solvents was done by using 3-fold argon-flushed disposable syringes and septa, while solids were added in an argon stream. Anhydrous THF was freshly distilled under potassium and stored over activated 3 Å molecular sieves. 1,2-Difluorobenzene (Apollo Scientific) was dried over 3 Å mol sieves. Deuterated solvents CDCl_3_ and THF-*d*_8_ were used as purchased and stored over activated 3 Å molecular sieves. NH_2_C_6_F_5_ and NH_2_C_6_Cl_5_ were purchased from BLD Pharm, CCl_4_ and AlCl_3_ from ABCR, CoCp_2_ from Sigma-Aldrich and Ni­(COD)_2_ from TCI.

### Nuclear Magnetic Resonance (NMR) Spectroscopy

NMR spectroscopy was measured on a JEOL ECX 400 (400 MHz) in the reported deuterated solvents THF-*d*_8_. The ^13^C NMR spectra are calibrated on the respective resonance signals of CDCl_3_ (δ = 77.16 ppm relative to tetramethylsilane). The ^19^F-spectra are internally reference to CFCl_3_. The given multiplicities are phenomenological; thus, the actual appearance of the signals is stated and not the theoretically expected one. The following abbreviations were used and analogously combined to designate multiplicities: s (singlet), d (doublet), t (triplet), q (quartet), m (multiplet), m_c_ (centrosymmetric multiplet). Evaluation of spectra was performed with Mestrelab Research MNova 7.^78^

### Infrared (IR) Spectroscopy

IR spectra were measured on an FT (Fourier transformation) Nicolet spectrometer. The samples were either measured directly or dispersed in Nujol oil before the measurement. In both cases, the spectra have been obtained by the ATR (attenuated total reflection) technique. Characteristic absorptions are given in wavenumbers ν̃ [cm^–1^] and intensities are stated as vs (very strong), s (strong), m (medium) and w (weak).

### Raman Spectroscopy

Raman spectra were recorded on a Bruker MultiRAM II Fourier-transform Raman spectrometer using a Nd:YAG laser (λ = 1064 nm) as the excitation source. The scattered radiation was detected with a liquid-nitrogen-cooled Ge detector operated at ca. 77 K. Spectra were acquired in a 180° backscattering geometry with a laser power of 60 mW at the sample, a spectral resolution of 2 cm^–1^, and 50 coadded scans. Powder samples were measured without dilution; sample heating or burning was prevented by the use of near-infrared excitation and moderate laser power. Raman bands are reported as wavenumbers ν̃ [cm^–1^] and intensities are stated as vs (very strong), s (strong), m (medium) and w (weak).

### Cyclic Voltammetry (CV)

Cyclic voltammetry was performed under an argon atmosphere in an electrochemical cell (Figure S1). A glassy carbon disc electrode (ϕ = 3 mm) was used as a working electrode and a silver wire was used for the counter- and the reference electrode, each. Samples were referenced against Fc^+/0^ by repeating the measurement with a substance with a known half-wave potential (Fc in the case of **Ni** ^**H**^, decamethylferrocene (Fc*) and N­(4–C_6_H_4_Br)_3_ in the case of **Ni** ^**F**^).

Sample solutions were prepared with a concentration of 10 mM with a supporting electrolyte (100 mM, [NBu]^+^[PF_6_]^−^ for **Ni** ^**H**^ or [NBu_4_]^+^[Al­(OC­(CF_3_)_3_)_4_]^−^ for **Ni** ^**F**^). The sweeping rate was varied between 20 and 1000 mV s^–1^ and was controlled and the response current signals were recorded on a Versastat 3–200 potentiostat (Princeton Applied Research).

### Optical Absorption Spectroscopy (UV–Vis)

Optical absorption spectroscopy was performed using a Cary 5000 instrument from Varian. Samples were measured in custom-built Schlenk-cuvettes with a path-length of 10 mm (Figure S2). The samples were prepared in an argon-filled glovebox.

### Transient Absorption Spectroscopy

Transient UV–vis absorption spectroscopy with subpicosecond time resolution was measured using a HARPIA-TA Instruments (Light Conversion). In this experimental setup, the excitation light is generated by a PHAROS laser (Light Conversion, Yb:KGW laser, source wavelength = 1030 nm, pulse duration ≈ 190 fs, repetition rate = 50 kHz, IRF ≈ 200–300 fs in THF, output power = 10 W, pulse energy = 0.2 mJ), and the actual pump light wavelength was generated by an optical parametric amplifier called ORPHEUS (Light Conversion, used ≈90% of fundamental pulse). The pulse energy at the sample was 0.22 μJ @ 390 nm, 0.18 μJ @ 370 nm and 0.16 μJ @ 360 nm. The probe light was generated by a sapphire (5 mm thickness; ≈10% of the fundamental pulse were used to generate a white light supercontinuum). Sample solutions (absorbance ≈ 0.3–0.4 at the excitation wavelength, see section 5 for more exact values) were measured in a 2 mm quartz cuvette at room temperature. Global analysis of the time-resolved data was performed using CarpetView (Light Conversion) based on species-associated spectra (SAS). The complete data set was analyzed simultaneously over the full spectral and temporal range, assuming that the measured signal can be described as a linear combination of distinct species with characteristic spectra governed by an explicit kinetic model. The SAS and associated time constants were obtained by global minimization of the residuals between experimental and calculated data. The instrument response function parameters (*D*_0_ and fwhm_*p*_) were fixed to values obtained from independent calibration, as early time data around time zero (below 0.5 ps) were excluded from the global analysis. In addition, the data was processed via a third party software (MATLAB v.R2024b) and analyzed globally using Optimus (v.3.04) to obtain the decay-associated spectra (DAS).^79^

### High-Resolution Mass Spectrometry (HRMS)

HRMS was recorded using a Varian MAT 711 spectrometer by electron impact ionization (EI) at the department of mass spectroscopy at the Freie Universität Berlin. A detailed listing of fragmentation is dispensed, instead the molecular ion peak or a characteristic fragment peak is stated.

### Single-Crystal X-ray Diffraction (XRD)

X-ray data were collected on a BRUKER D8 Venture system. Data were collected at 100(2) or 150(2) K using graphite monochromated Mo Kα radiation (λ_α_ = 0.71073 Å). The strategy for the data collection was evaluated by using the Smart software. The data were collected by the standard “ψ-ω scan techniques” and were scaled and reduced using Saint+software. The structure was solved by using Olex2,^80^ the structure was solved with the XT structure solution program^81^ using Intrinsic Phasing and refined with the XL refinement package using Least Squares minimization.^82,83^ Drawings were generated and the geometric parameters were measured with Mercury.^84^

Hirshfeld surfaces were generated using CrystalExplorer version 21.5 based on the refined single-crystal X-ray diffraction data.^57,58^ The surfaces were mapped with the normalized contact distance (d_norm), which highlights intermolecular contacts shorter (red), equal to (white), or longer (blue) than the sum of the van der Waals radii. Two-dimensional fingerprint plots were derived from the Hirshfeld surfaces to quantify the relative contributions of different intermolecular interactions (e.g., C···X, X···X, C···C; X = H, F, Cl) to the overall crystal packing.

### Computational Details

All quantum-chemical calculations were performed using Gaussian 16 (Revision C.01). Initial geometries were optimized in the gas phase using density functional theory with the B3LYP functional in combination with the def2-TZVP basis set for all atoms.^85^ Empirical dispersion corrections were included using Grimme’s D3 scheme with Becke-Johnson damping (GD3BJ). Geometry optimizations employed default tight convergence criteria as implemented in Gaussian.^86–88^

Harmonic vibrational frequency calculations were carried out at the same level of theory to verify that the optimized structures correspond to true minima on the potential energy surface; no imaginary frequencies were found.

Electronic excitation energies and oscillator strengths were computed using time-dependent DFT (TD-DFT) at the B3LYP/def2-TZVP level, based on the optimized ground-state geometries. Full TD-DFT calculations were performed for singlet excited states (TD = singlets, nstates = 50–100); the Tamm-Dancoff approximation was not applied. Oscillator strengths were obtained directly from the TD-DFT transition dipole moments as implemented in Gaussian.

Energy decomposition analysis (EDA) combined with Natural Orbitals for Chemical Valence (NOCV) was performed using the ADF engine of the Amsterdam Modeling Suite (AMS) 2024.101.^89^ Scalar relativistic effects were treated using the ZORA formalism, and dispersion interactions were included via Grimme’s D3 correction with Becke-Johnson damping. The EDA-NOCV calculations were carried out with high numerical accuracy (verygood) to analyze the bonding interactions between the two fragments of the system. Input geometries were obtained using the computational protocol described above, with BP86 employed in place of B3LYP to ensure consistency with established EDA-NOCV methodologies. All EDA-NOCV calculations were performed using the BP86 functional in combination with a TZ2P Slater-type basis set.

Molecular structures, natural transition orbitals and molecular orbitals were visualized using ChemCraft (version 1.8).

#### Dichloro-N-1,2,3,4,5-pentachlorophenyl Imine

Synthesized according to the literature.^90^ In a 250 mL Schlenk flask pentachloroaniline (12.0 g, 0.0452 mol, 1.00 equiv) and AlCl_3_ (18.1 g, 0.136 mol, 3.00 equiv) were suspended in 84 mL of CCl_4_. The reaction mixture was heated to reflux (Attention: HCl evolution!) and stirred for 48 h after which the mixture was quenched with ice. The aqueous mixture was extracted with Et_2_O (3 × 50 mL) and dried with MgSO_4_. The crude product was purified by column chromatography (*n*-hexane). A brown-yellow crystalline solid of Cl_2_CNC_6_Cl_5_ (9.01 g, 0.0259 mol) was obtained with a yield of 57%.

^**13**^ **C­{** ^**1**^ **H}-NMR** (101 MHz, CDCl_3_, rt) δ [ppm] = 141.4, 136.7, 132.3, 130.4, 123.3.

**HRMS** (EI-TOF, positive) *m*/*z* for [Cl_2_CNC_6_Cl_5_]^+^: 344.782; found 344.775.

**FT-IR** (ATR) ν̃ [cm^–1^] = 2923 (w), 1841 (w), 1656 (s), 1355 (vs), 1307 (m), 1248 (s), 1134 (w), 1037 (w), 968 (w), 915 (vs), 770 (s), 723 (vs), 653 (s), 574 (vs).

#### 1,2,3,4,5-Pentachlorophenyl Isocyanide

A 100 mL Schlenk flask was charged with cobaltocene (1.09 g, 5.70 mmol, 2.00 equiv) and Cl_2_CNC_6_Cl_5_ (1.00 g, 2.88 mmol, 1.00 equiv) and THF (30 mL) was added. The reaction was stirred at room temperature for 24 h. The solvent of the yellow-red suspension was evaporated *in vacuo* and the residue sublimed at 100 °C (0.003 mbar) using a cooling finger of 0 °C. A white solid of CN–C_6_Cl_5_ (0.220 g, 0.789 mmol) was received with a yield of 27%.

^**13**^ **C­{** ^**1**^ **H}-NMR** (101 MHz, CDCl_3_, rt) δ [ppm] = 178.1, 135.0, 132.6, 130.6, 125.2.

**HRMS** (EI-TOF, positive) *m*/*z* for [CNC_6_Cl_5_]^+^ calculated: 274.844; measured: 274.848.

**FT-IR** (ATR) ν̃ [cm^–1^] = 2115 (s), 1608 (w), 1360 (vs), 1303 (m), 1234 (m), 920 (w), 804 (w), 742 (vs), 720 (s), 653 (m).

**Raman** ν̃ [cm^–1^] = 2124 (s), 1532 (s), 1245 (m), 385 (m), 320 (m), 213 (w), 150 (m), 107 (m).

#### Tetrakis­(1,2,3,4,5-pentafluorophenyl isocyanide)­nickel(0)

Ni­(COD)_2_ (24.0 mg, 0.0840 mmol, 1.00 equiv) was filled into a 25 mL Schlenk flask and subsequently a solution of CNC_6_F_5_ (66.0 mg, 0.336 mmol, 6.00 equiv) in THF (5 mL) was condensed onto it at –196 °C. The solution was warmed to room temperature and stirred for 2 h. The yellow black solution was evaporated in *vacuo* and extracted using Et_2_O (2 × 2 mL) into a 25 mL Schlenk flask. The yellow black solution was stirred in the presence of air and discolored completely to bright yellow. The product was filtered through a syringe filter and the solvent evaporated *in* vacuo yielding [Ni­(CNC_6_F_5_)_4_] (32.0 mg, 0.0390 mmol) as a yellow solid in a yield of 45%.

^**19**^ **F-NMR** (377 MHz, THF-*d*_8_, rt) δ [ppm] = – 146.9 (m, *ortho*, 2F), – 157.4 (m, *para*, 1F), – 163.6 (m, *meta*, 2F).

^**13**^ **C­{** ^**19**^ **F}-NMR** (101 MHz, THF-*d*_8_, rt) δ [ppm] = 183.7, 143.4, 140.9, 139.1, 107.3).

**HRMS** (ESI-TOF, negative) *m*/*z* for [C_28_F_20_N_4_NiCl]^−^ calculated: 864.8864; measured: 864.9170.

**FT-IR** (ATR) ν̃ [cm^–1^] = 2962 (w), 2033 (s), 1650 (vs), 1507 (w), 1258 (m), 1090 (m), 1019 (s), 978 (vs), 795 (vs), 612 (m), 424 (m).

**Raman** ν̃ [cm^–1^] = 2969 (w), 2908 (w), 2145 (w), 2049 (s), 1653 (vs), 1513 (m), 1460 (m), 1324 (m), 987 (w), 614 (m), 566 (m), 463 (w), 426 (w), 380 (w), 335 (w), 119 (w), 62 (m).

All the spectroscopic data obtained by NMR and IR spectroscopy are consistent with the values reported in literature.^47^

#### Tetrakis­(phenyl isocyanide)­nickel(0)

Ni­(CNC_6_H_5_)_4_ was synthesized according to literature procedure^56^ by combining Ni­(COD)_2_ with and an excess of phenyl isocyanide in toluene at room temperature. After stirring for an hour the solution was brought to –20 °C to crystallize the Ni­(CN–C_6_H_5_)_4_ in 63% yield in the course of 3 days.

#### Tetrakis­(1,2,3,4,5-pentachlorophenyl isocyanide)­nickel­(0)

A 10 mL Schlenk flask was charged with Ni­(COD)_2_ (16.0 mg, 0.0590 mmol, 1.00 equiv) and dissolved in THF (4 mL). CNC_6_Cl_5_ (66.0 mg, 0.239 mmol, 4.00 equiv) was added to the solution and a bright red solid precipitated immediately. The solvent was decanted and the residue dried. The crude product was recrystallized in boiling CCl_4_ and cooled to room temperature overnight. Bright red crystals of [Ni­(CNC_6_Cl_5_)_4_] were received with a yield of 50%.

Due to low solubility a ^13^C NMR experiment did not lead to any sufficient signal.

**HRMS** (ESI-TOF, positive) *m*/*z* for [C_21_Cl_15_N_3_NiK]^+^ calculated: 923.7937; measured: 923.7418.

**FT-IR** (ATR) ν̃ [cm^–1^] = 1966 (vs), 1522 (s), 1376 (s), 1351 (s), 1296 (m), 1247 (s), 1065 (w), 966 (w), 716 (s).

**Raman** ν̃ [cm^–1^] = 2006 (s), 1526 (vs), 1382 (m), 1252 (m), 967 (w), 507 (w), 431 (w), 348 (w), 93 (w).

#### Pentacarbonyl­(1,2,3,4,5-pentachlorophenyl isocyanide)­chromium­(0)

Following a modified procedure for (OC)_5_Cr­(CN–C_6_F_5_).^70^ Instead of CN–C_6_F_5_, CN–C_6_Cl_5_ was used. A 300 mL Schlenk flask was filled with Cr­(CO)_6_ (2.00 g, 8.84 mmol, 12.00 equiv), dissolved in THF (250 mL) subjected to three freeze–pump–thaw cycles. The solution was warmed to room temperature and irradiated for 2 h. A solution of CNC_6_Cl_5_ (200 mg, 0.72 mmol, 1.00 equiv) in THF (5 mL) was added to the orange solution and stirred for 2 h. The now yellow solution was evaporated to dryness. Unreacted Cr­(CO)_6_ was recovered using sublimation (4 × 10^–2^ mbar, 40 °C) for 6 h. The resulting residue was extracted using C_6_H_6_ and filtered under an argon atmosphere. The bright yellow filtrate was evaporated to dryness and the residue dissolved in *n*-pentane (10 mL) and stored in a –70 °C freezer. The crude product was purified by column chromatography (*n*-pentane). Bright yellow crystals of [Cr­(CO)_5_(CNC_6_Cl_5_)] (226 mg, 0.48 mmol) with a yield of 66% were received.

^**13**^ **C­{** ^**1**^ **H}-NMR** (101 MHz, CDCl_3_, rt) δ [ppm] = 215.1, 213.6, 188.8, 133.9, 132.7, 130.2, 119.6.

**HRMS** (EI-TOF, positive) *m*/*z* for [CrC_12_NCl_5_O_5_]^+^ calculated: 466.7594; measured: 466. 7647

**FT-IR** (ATR) ν̃ [cm^–1^] = 2125 (m), 2035 (m), 1937 (vs), 1360 (m), 1258 (s), 1081 (s), 1011 (vs), 862 (w), 790 (vs), 716 (m), 645 (s)

**Raman** ν̃ [cm^–1^] = 2127 (m), 2034 (vs) 2005 (m), 1547 (m), 1530 (s), 1388 (w), 1252 (w), 500 (w), 438 (w), 390 (m), 119 (m), 100 (m).

## Supplementary Material



## Notes

Detailed experimental procedures and characterization data for all compounds discussed in this work, including full synthetic protocols, vibrational and NMR spectroscopic data, and crystallographic details, additional information on the transient absorption spectroscopy experiments, Cartesian coordinates of all DFT-optimized structures, together with the corresponding TD-DFT excitation energies, oscillator strengths, and natural transition orbital (NTO) representations, and thermochemical data derived from the DFT calculations as well as the results of the energy decomposition analysis using the EDA-NOCV method (PDF)
