# Supplementary material for: Controlling Redox and Photophysical Properties of First-Row Transition Metal Complexes via Ligand Perhalogenation
Source: Inorg Chem. 2026 Feb 28;65(10):5374–86. doi: 10.1021/acs.inorgchem.5c05333 (PMC12997154; doi:10.1021/acs.inorgchem.5c05333)
Supplement: Supplementary file 1 [file ic5c05333_si_001.pdf]

Supporting Information

on

Controlling Redox and Photophysical Properties of First-Row Transition Metal Complexes via Ligand Perhalogenation

Tim-Niclas Streit,[#a] Malte Sellin,[#*b] Bruno Lazarevski,[b] Oliver S. Wenger,[*b] Moritz Malischewski[*a]

[a] Freie Universität Berlin
Institut für Chemie und Biochemie
Fabeckstraße 34–36
14195 Berlin, Germany

[b] University of Basel
Department of Chemistry
St. Johanns-Ring 19
4056 Basel, Switzerland

Contact Details

Dr. Malte Sellin – malte.sellin@unibas.ch

Prof. Dr. Oliver Wenger – oliver.wenger@unibas.ch

Dr. Moritz Malischewski – moritz.malischewski@fu-berlin.de

Table of Contents

1. Specialized Glasware 3
2. Vibrational Spectroscopy 4
3. NMR Spectroscopy 9
4. Single-Crystal X-Ray Diffraction 12
5. Transient Absorption Spectroscopy 17
6. Optimized Structures 25
7. TD-DFT Calculations 30
8. Thermochemistry 51
9. EDA-NOCV Analysis 51
10. References 52

1. Specialized Glasware

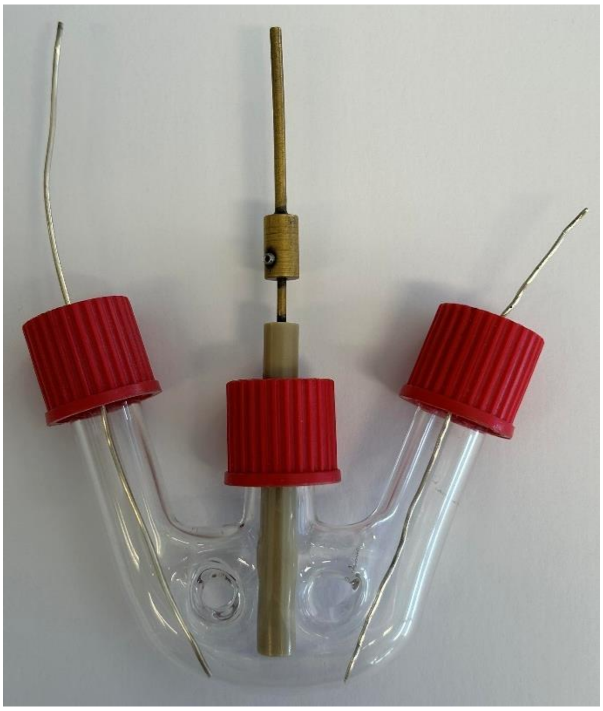

Figure S1: Three-electrode set-up for cyclic voltammetry.

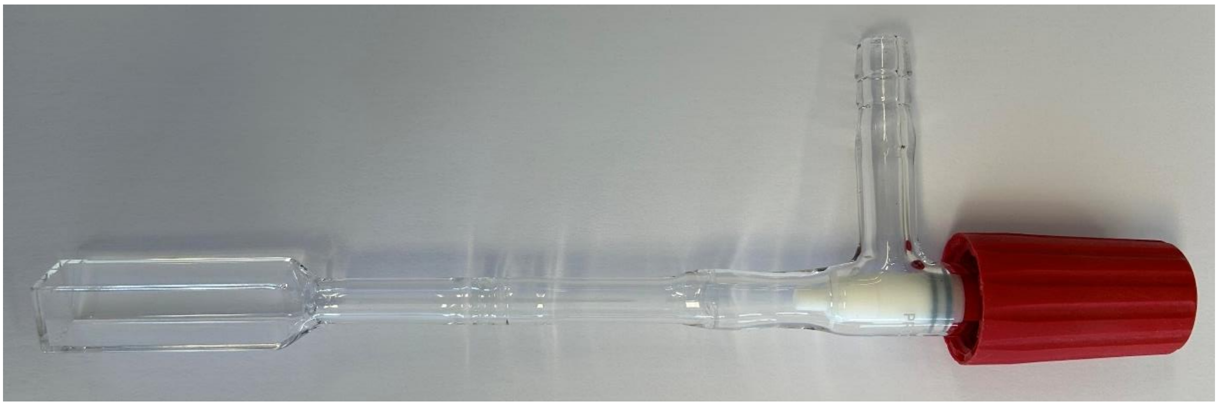

Figure S2: Custom-built Schlenk cuvette with a path-length of 10 mm and quartz glass. Valve from PRODURAN.

2. Vibrational Spectroscopy

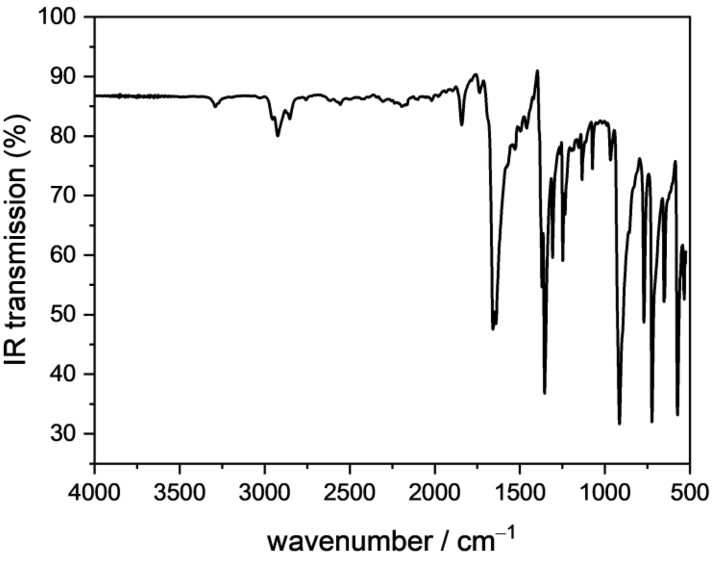

Figure S3: ATR-IR spectrum of $Cl_2C{=}N{-}C_6Cl_5$.

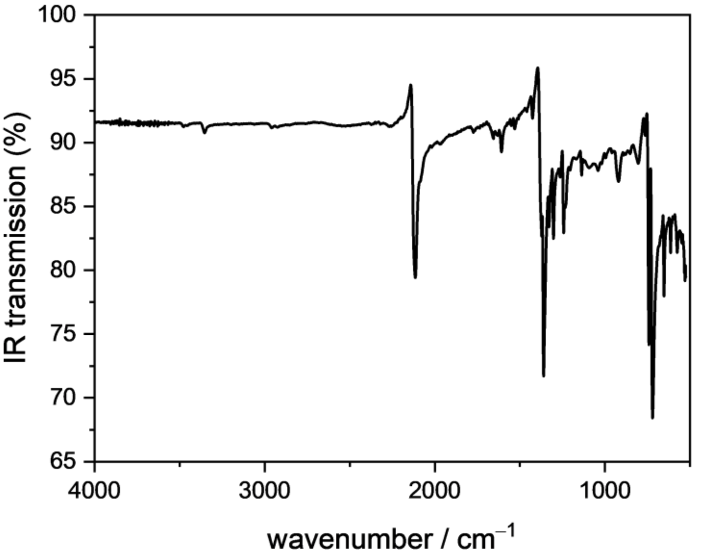

Figure S4: ATR-IR spectrum of $CN{-}C_6Cl_5$.

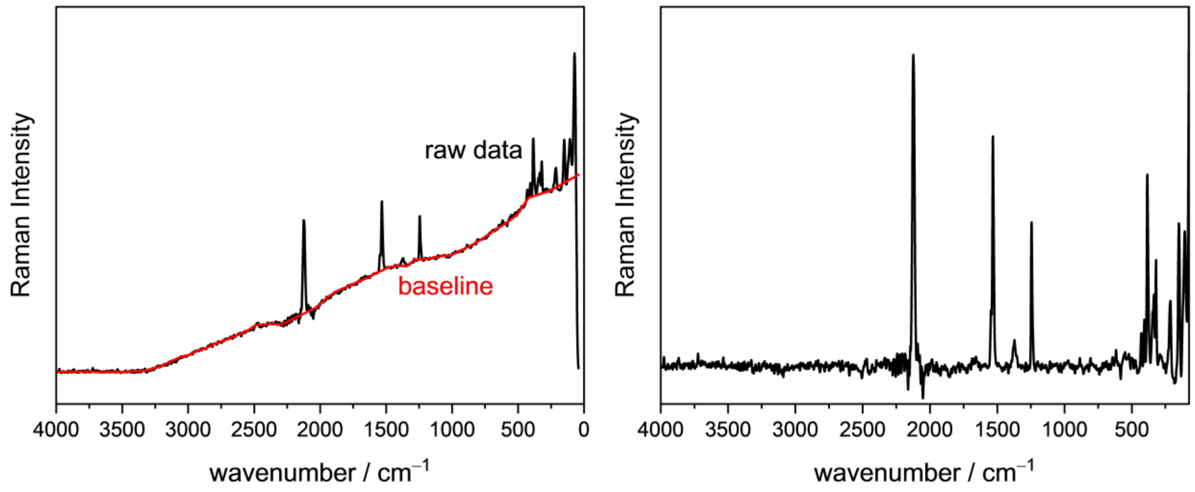

Figure S5: Left: raw Raman spectrum of CN–C_6Cl_5 (black) and baseline correction (red). Right: Baseline corrected Raman spectrum of CN–C_6Cl_5. Baseline correction was performed using a derivative-based algorithm, in which baseline anchor points were identified from zero crossings of the first and second derivatives (50 points), followed by polynomial smoothing (order 2) using a smoothing window of 3 points and a threshold of 0.05.

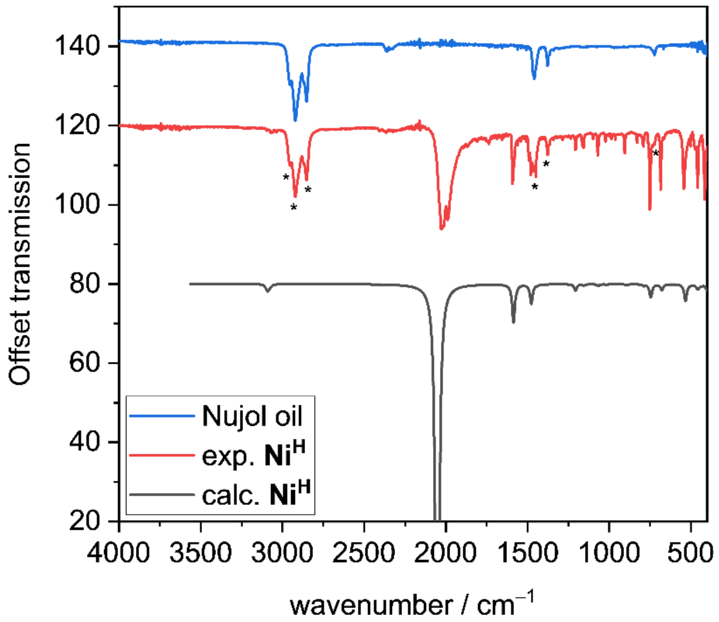

Figure S6: Comparison of the ATR-IR spectra of Nujol oil (blue), **Ni^{H}** dispersed in Nujol oil (red) and the calculated spectrum of **Ni^{H}** (grey line, B3LYP(D3BJ)/def2-TZVP level of theory, scaled by 0.967, Lorentzian broadening, fwhm 20 cm^{-1}). Asterisks (*) denote bands stemming from Nujol oil.

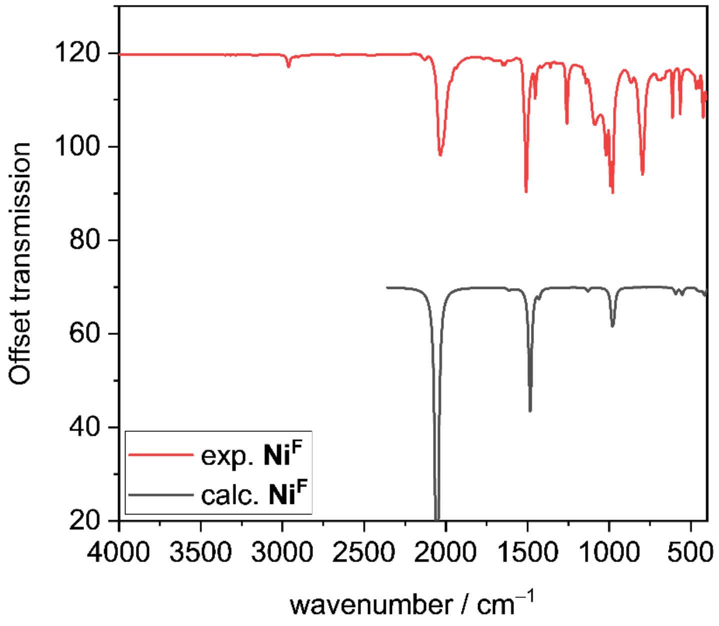

Figure S7: Comparison of the ATR-IR spectrum **Ni**$^{\mathbf{F}}$ powder (red) and the calculated spectrum of **Ni**$^{\mathbf{Cl}}$ (grey line, B3LYP(D3BJ)/def2-TZVP level of theory, scaled by 0.967, Lorentzian broadening, fwhm 20 cm^{-1}).

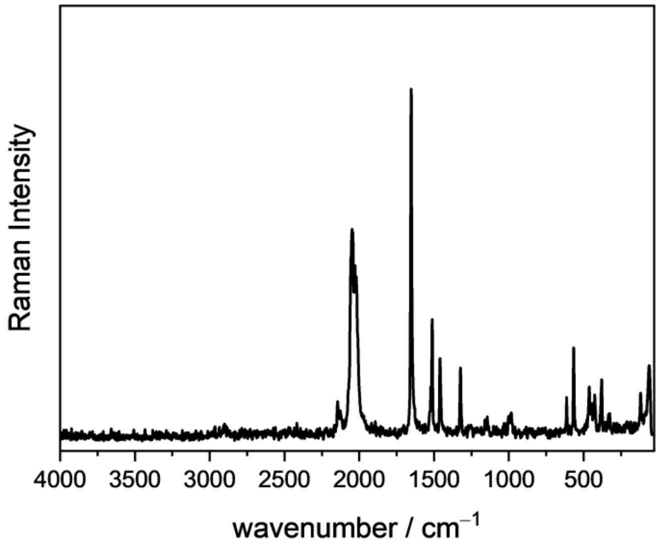

Figure S8: Raman spectrum of **Ni**$^{\mathbf{F}}$ (powder).

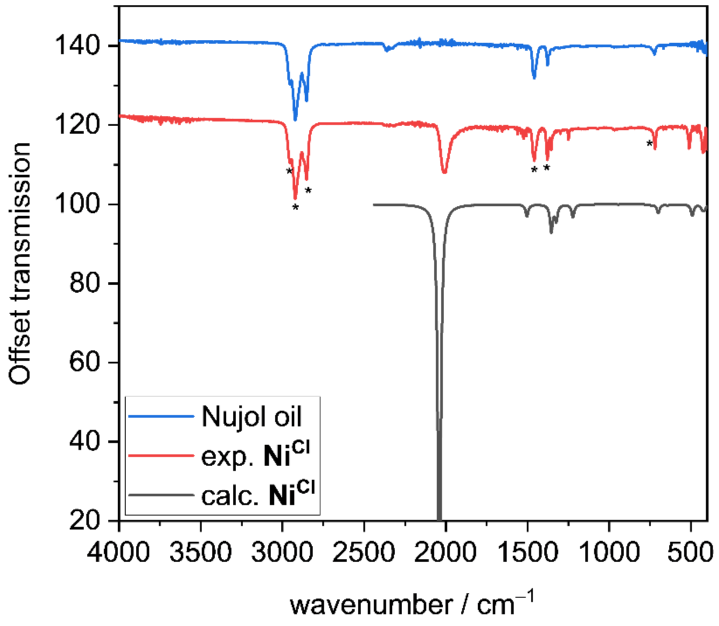

Figure S9: Comparison of the ATR-IR spectra of Nujol oil (blue), **Ni^{Cl}** dispersed in Nujol oil (red) and the calculated spectrum of **Ni^{Cl}** (grey line, B3LYP(D3BJ)/def2-TZVP level of theory, scaled by 0.967, Lorentzian broadening, fwhm 20 cm^{-1}). Asterisks (*) denote bands stemming from Nujol oil.

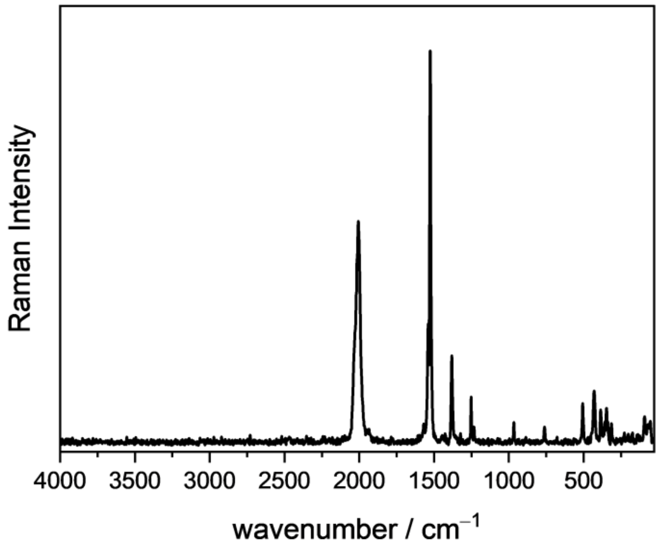

Figure S10: Raman spectrum of **Ni^{Cl}** (powder).

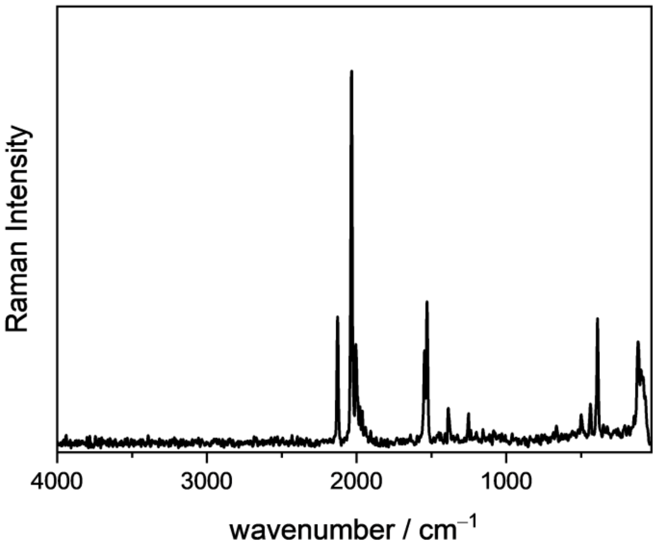

Figure S11: Raman spectrum of $Cr(CO)_5(CN–C_6Cl_5)$ (powder).

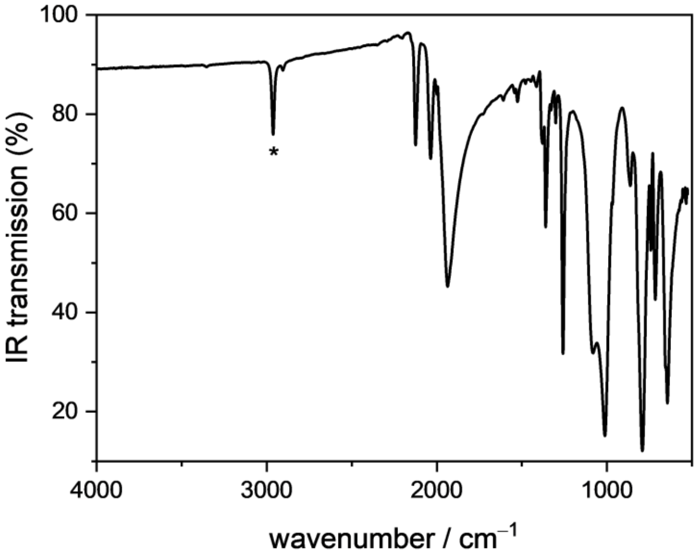

Figure S12: ATR-IR spectrum of $Cr(CO)_5(CN–C_6Cl_5)$ (powder). Asterisk (*) denotes residual THF.

3. NMR Spectroscopy

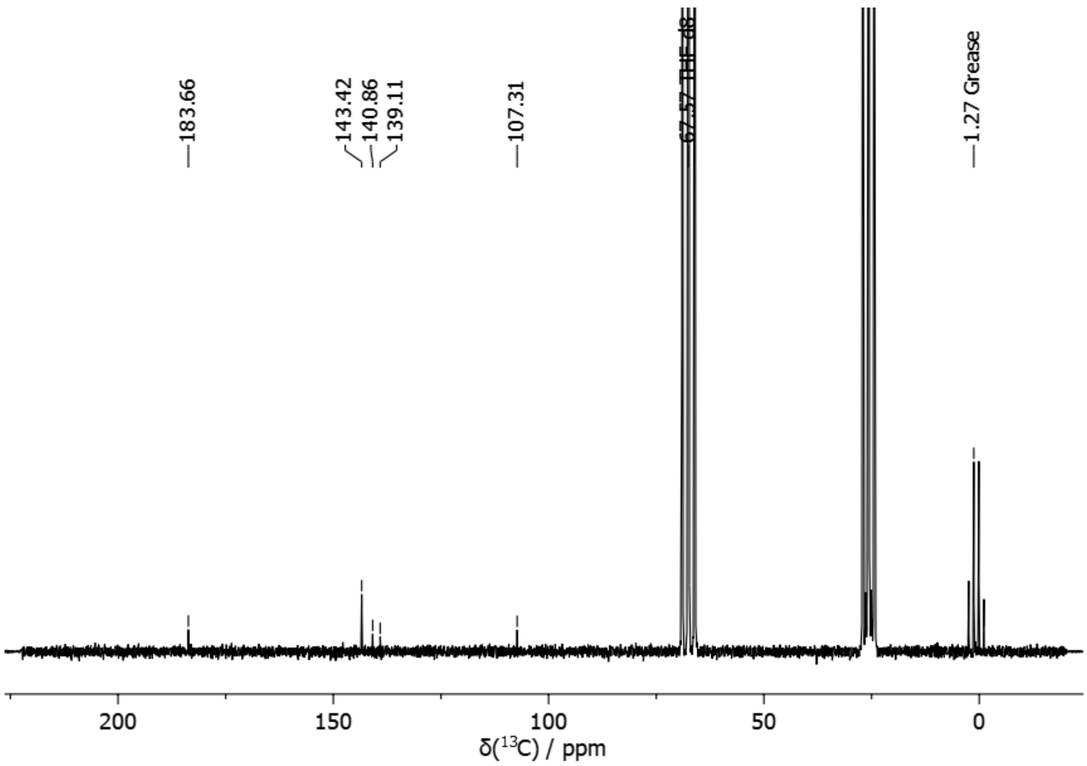

Figure S13: $^{13}C\{^{19}F\}$-NMR (101 MHz, THF-d_8, rt) spectrum of $Ni(CNC_6F_5)_4$ (**Ni^F**).

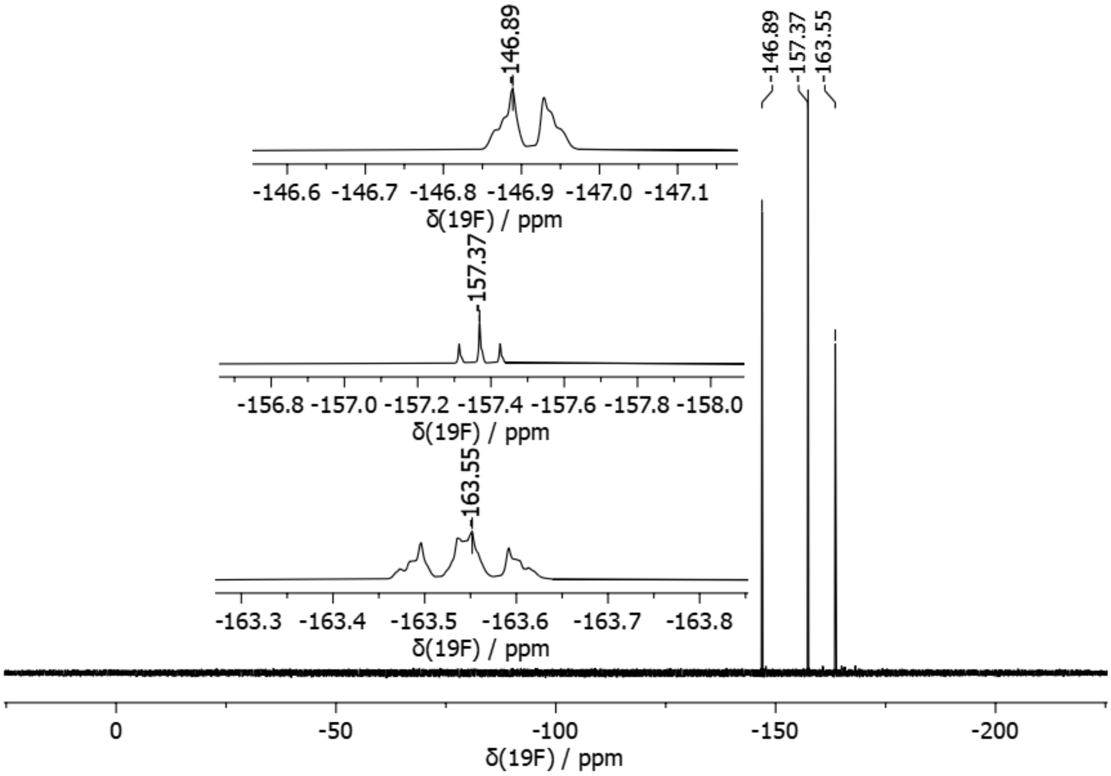

Figure S14: ^{19}F-NMR (371 MHz, THF-d_8, rt) spectrum of $Ni(CNC_6F_5)_4$ (**Ni^F**).

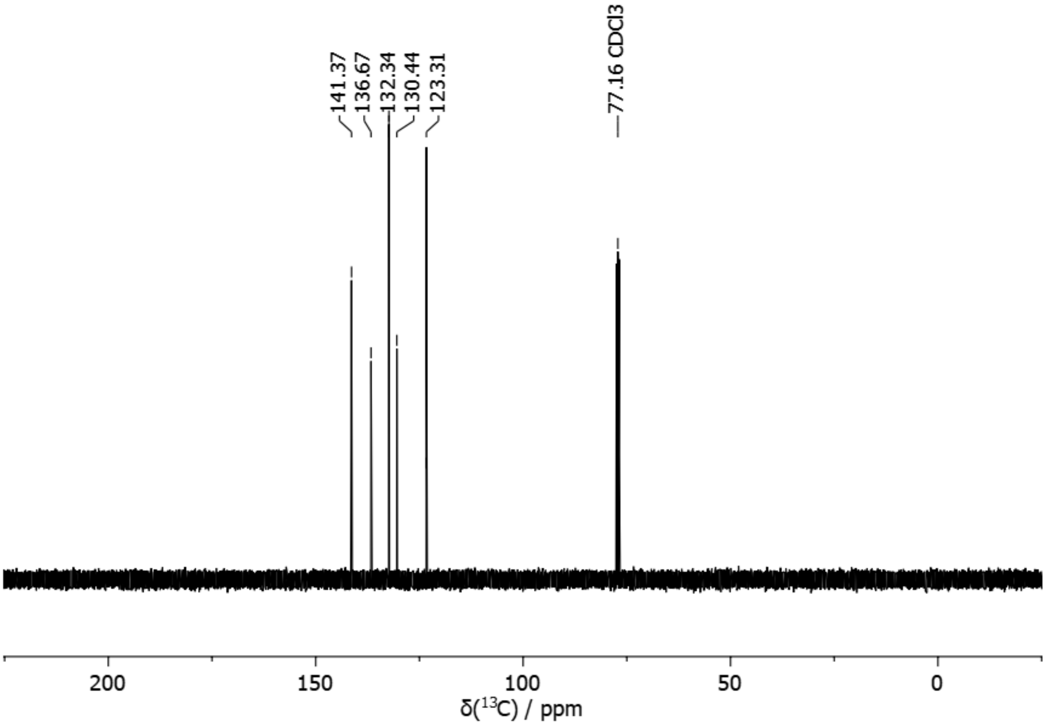

Figure S15: $^{13}C\{^{1}H\}$-NMR (101 MHz, THF-d_8, rt) spectrum of $Cl_2CNC_6Cl_5$.

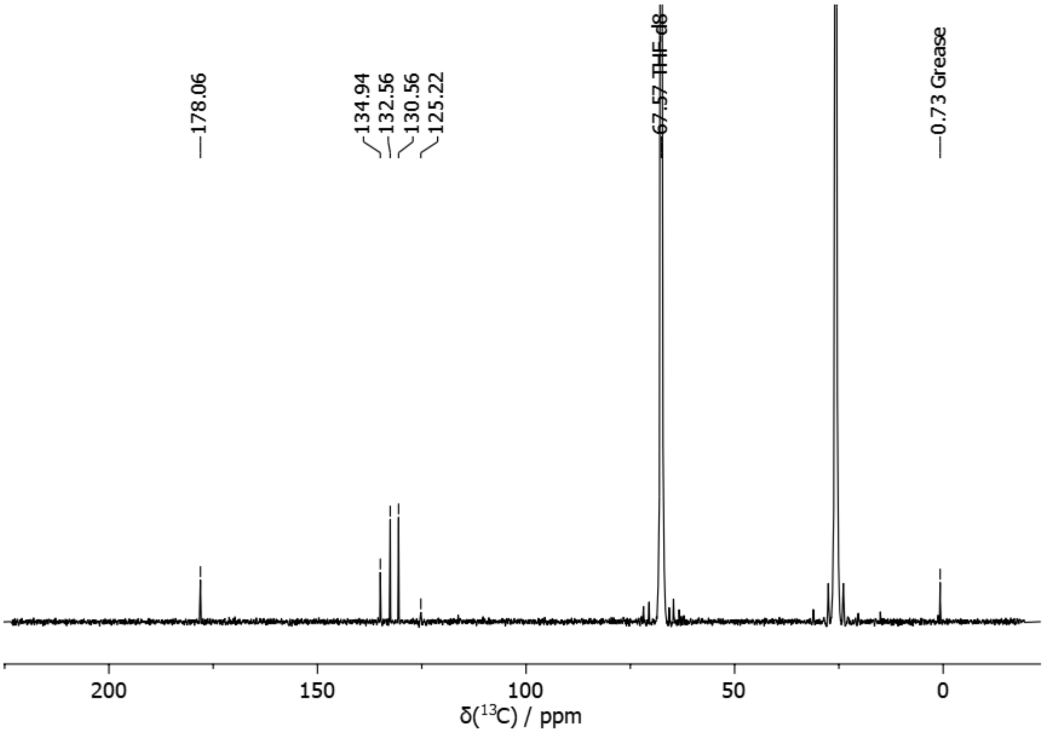

Figure S16: $^{13}C\{^{1}H\}$-NMR (101 MHz, THF-d_8, rt) spectrum of CNC_6Cl_5.

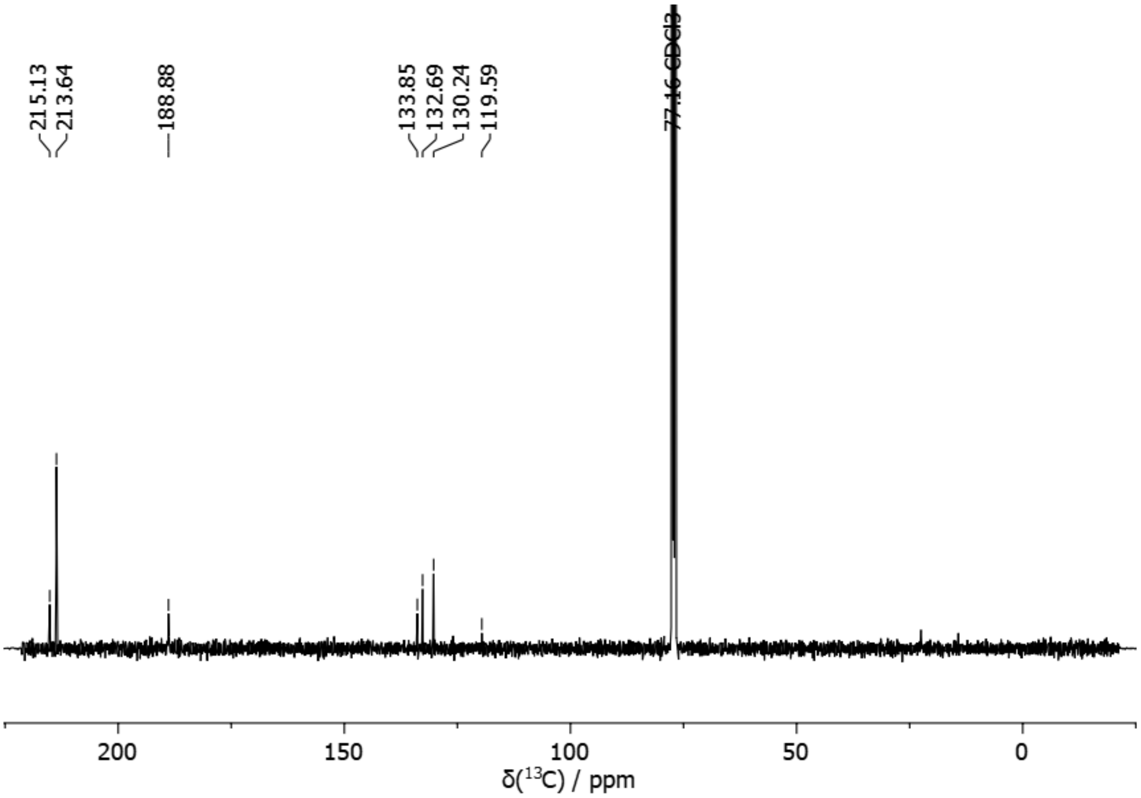

Figure S17: $^{13}C\{^{1}H\}$-NMR (101 MHz, $CDCl_3$, rt) spectrum of $Cr(CO)_5(CNC_6Cl_5)$.

4. Single-Crystal X-Ray Diffraction

Table S1: Crystallographic data tables for $Ni(CN–C_6F_5)_4$ (**Ni^F**), $Ni(CN–C_6Cl_5)_4$ (**Ni^{Cl}**) and $Cr(CO)_5(CN–C_6Cl_5)$.

**Compound**	**$Ni(CN–C_6F_5)_4$**	**$Ni(CN–C_6Cl_5)_4$**	**$Cr(CO)_5(CN–C_6Cl_5)$**
**CCDC number**	2488101	2488102	2488103
**Empirical formula**	$C_{28}F_{20}N_4Ni$	$C_{28}Cl_{20}N_4Ni$	$C_{12}Cl_5CrNO_5$
**Formula weight**	831.03	1160.03	467.38
**Temperature/K**	150.00	100.00	150.00
**Crystal system**	monoclinic	monoclinic	monoclinic
**Space group**	$P2_1/n$	C2/c	C2/c
**a/Å**	25.0840(16)	23.6398(9)	24.1153(14)
**b/Å**	4.9896(3)	11.0874(5)	6.3369(3)
**c/Å**	25.3863(16)	16.7657(8)	22.0272(13)
**α/°**	90	90	90
**β/°**	119.135(2)	116.8500(10)	106.477(2)
**γ/°**	90	90	90
**Volume/$Å^3$**	2775.3(3)	3920.6(3)	3227.9(3)
**Z**	4	4	8
**ρ_{calc}g/cm^3**	1.989	1.965	1.923
**μ/mm^{-1}**	0.865	1.891	1.559
**F(000)**	1616.0	2256.0	1824
**Crystal size/mm^3**	0.447 × 0.067 × 0.058	0.12 × 0.12 × 0.1	0.07×0.163×0.533
**Radiation**	MoKα (λ = 0.71073)	MoKα (λ = 0.71073)	MoK_α (λ=0.71073 Å)
**2Θ range for data collection/°**	4.884 to 50.096	4.15 to 50.852	4.42 to 50.89 (0.83 Å)
**Index ranges**	-29 ≤ h ≤ 29, -5 ≤ k ≤ 5, -30 ≤ l ≤ 30	-28 ≤ h ≤ 28, -13 ≤ k ≤ 13, -20 ≤ l ≤ 20	−28 ≤ h ≤ 28 −6 ≤ k ≤ 7 −26 ≤ l ≤ 26
**Reflections collected**	67972	32330	31361
**Independent reflections**	4883 [R_{int} = 0.0598, R_{sigma} = 0.0265]	3612 [R_{int} = 0.0516, R_{sigma} = 0.0261]	2983 R_{int} = 0.0673 R_{sigma} = 0.0300
**Data/restraints/parameters**	4883/516/578	3612/0/242	2983 / 0 / 217
**Goodness-of-fit on F^2**	1.223	1.160	1.085
**Final R indexes [I>=2σ (I)]**	R_1 = 0.0682, wR_2 = 0.1652	R_1 = 0.0364, wR_2 = 0.0711	R_1 = 0.0406 wR_2 = 0.0682
**Final R indexes [all data]**	R_1 = 0.0756, wR_2 = 0.1694	R_1 = 0.0532, wR_2 = 0.0860	R_1 = 0.0551 wR_2 = 0.0751
**Largest diff. peak/hole / e $Å^{-3}$**	0.88/-0.54	0.22/-0.20	0.34/−0.34

Table S2: Key distances and angles of the **Ni^{X}** complexes from their solid-state structures in comparison to the calculated values (in italics, B3LYP(D3BJ)/def2-TZVP level of theory)

Ni(CN–R)$_4$	R = –C_6H_5 ^{a}	R = –C_6F_5	R = –C_6Cl_5
Ni–C (Å)	1.828(1)<br>*1.852*	1.814–1.834(5)<br>*1.844*	1.829–1.831(1)<br>*1.842–1.845*
C≡N (Å)	1.162(4)<br>*1.177*	1.174–1.185(7)<br>*1.178*	1.171–1.174(6)<br>*1.777–1.779*
N–C (Å)	1.388(5)<br>*1.371*	1.360–1.379(7)<br>*1.356*	1.375–1.377(5)<br>*1.355*
∠(C≡N–C) (°)	168.3(3)<br>*179.6*	175.6–179.0(5)<br>*177.4*	173.0(4)<br>*176.7–176.9*

^{a} taken from ref[1]

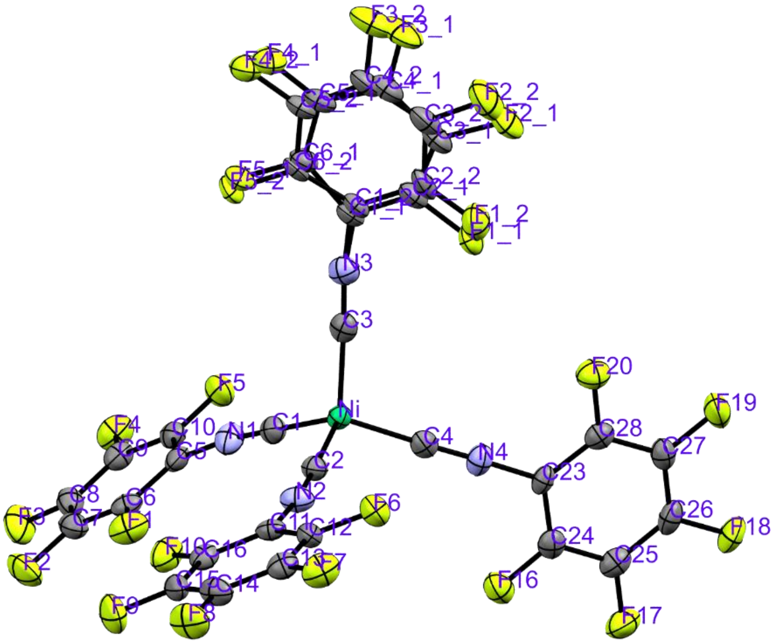

Figure S18: Asymmetric unit of **Ni^{F}**. Thermal displacement ellipsoids set at 50% probability.

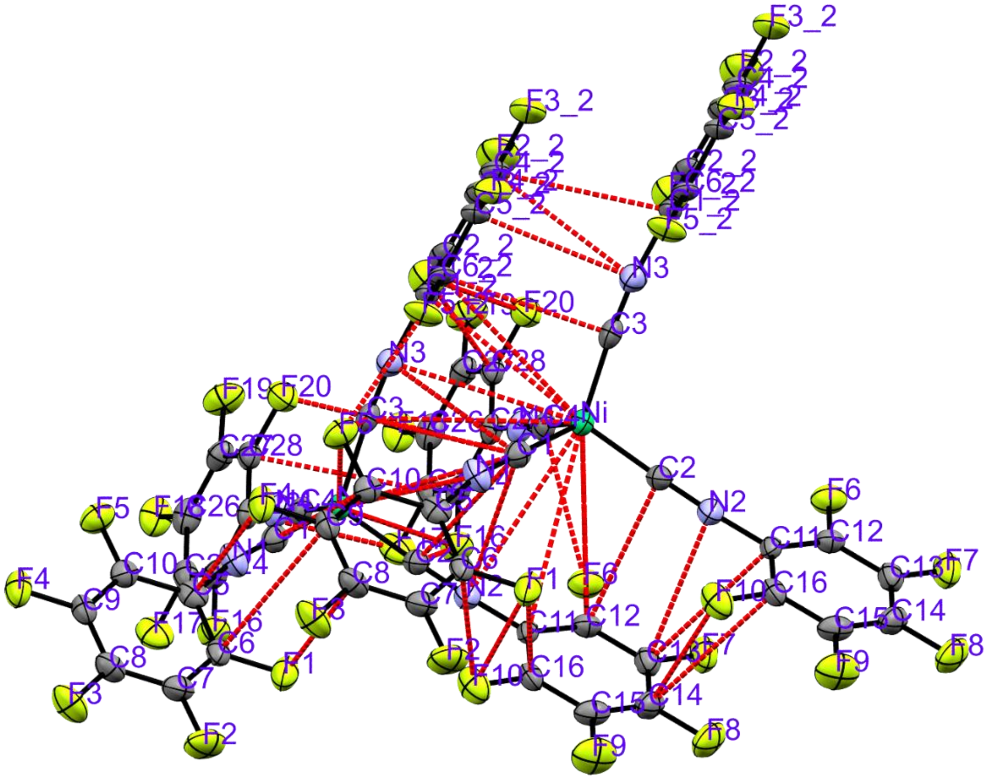

Figure S19: π-Stacking between two **Ni^{F}** moieties. Thermal displacement ellipsoids set at 50% probability. Intermolecular contacts below the sum of the VdW radii are shown as red dotted lines.

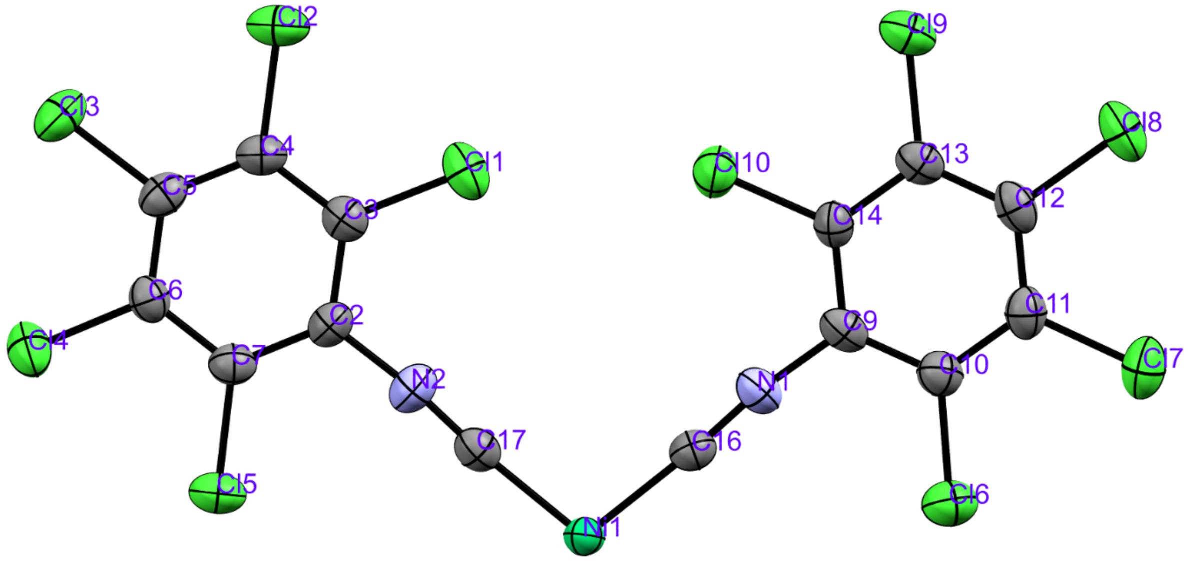

Figure S20: Asymmetric unit of **Ni^{Cl}**. Thermal displacement ellipsoids set at 50% probability.

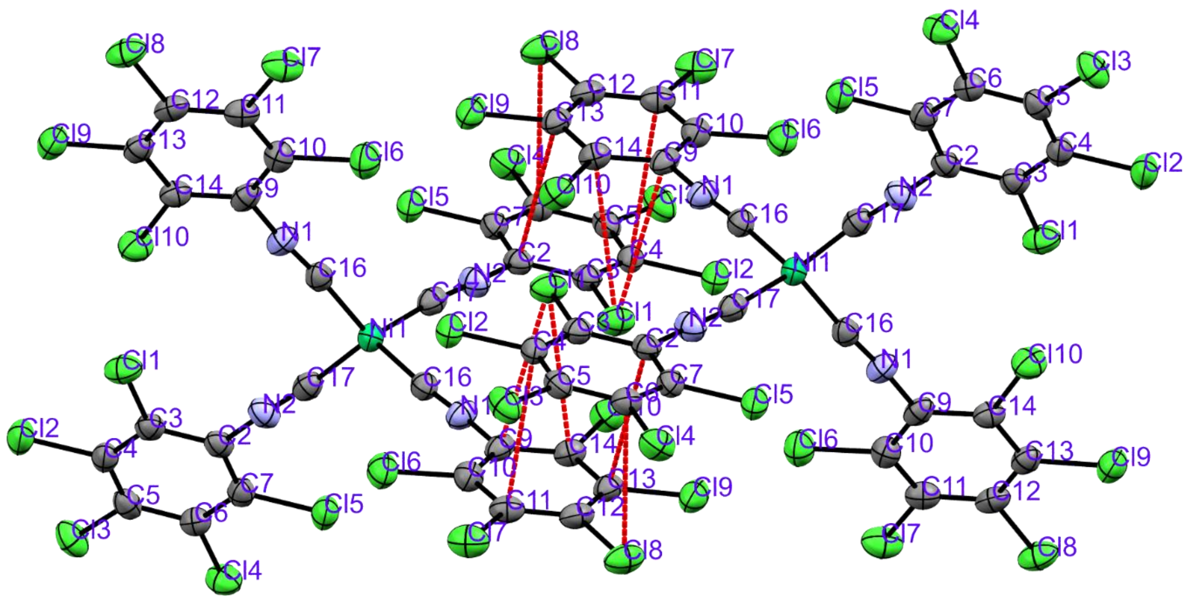

Figure S21: π-Stacking between two **Ni^{Cl}** moieties. Thermal displacement ellipsoids set at 50% probability. Intermolecular contacts below the sum of the VdW radii are shown as red dotted lines.

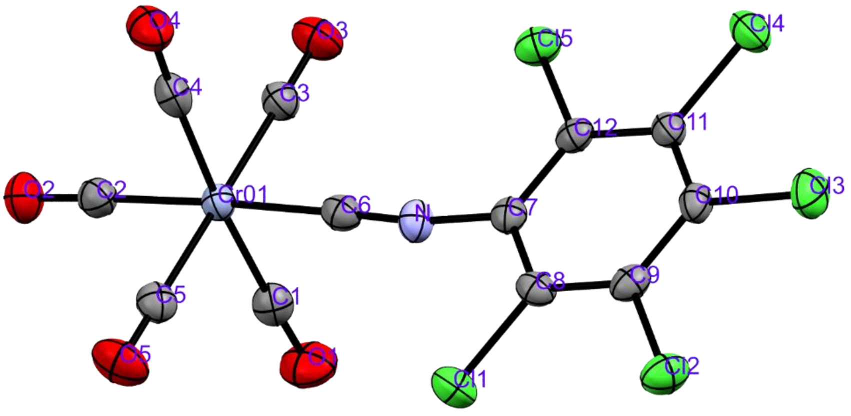

Figure S22: Asymmetric unit of $Cr(CO)_5(CN–C_6Cl_5)$. Thermal displacement ellipsoids set at 50% probability.

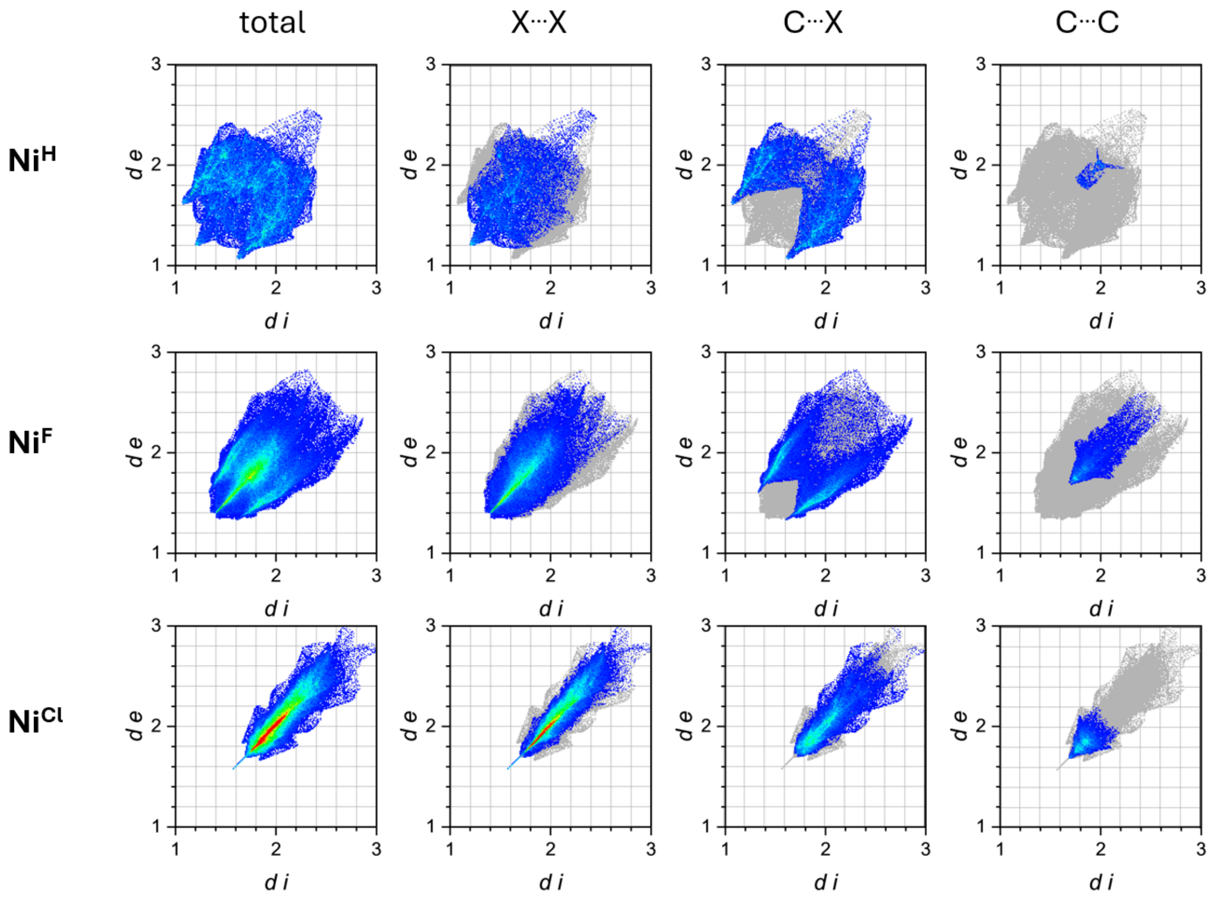

Figure S23: Hirshfeld surface fingerprint plots of **Ni^H** (top), **Ni^F** (middle) and **Ni^{Cl}** (bottom). *d e* = external core – surface distance; *d i* = internal core – Hirshfeld surface distance. Increasing number of points goes from blue over green and yellow to red. Distances are given in Å.

5. Transient Absorption Spectroscopy

A UV-vis spectrum from the sample was taken before and after the TAS measurement to quantify the relative substance loss at the excitation wavelength through the measurement according to eq. 1.

$$\Delta A_{rel}(\lambda) = \frac{A_{pre}(\lambda) - A_{post}(\lambda)}{A_{pre}(\lambda)} \qquad \text{(eq. 1)}$$

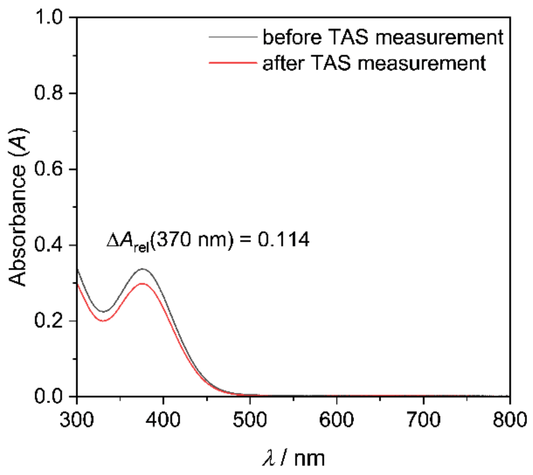

Figure S24: UV-vis spectrum of a sample of **Ni^{H}** in THF taken before and after the TAS measurement.

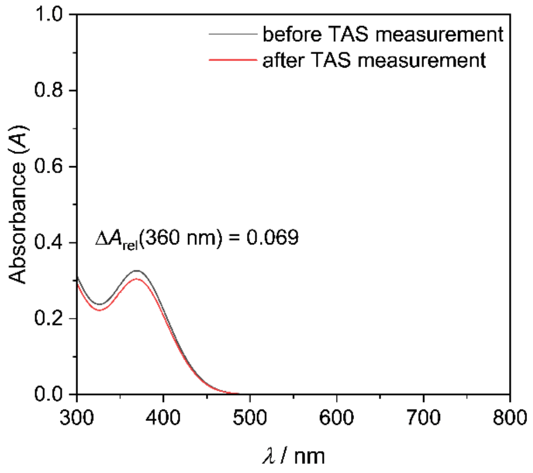

Figure S25: UV-vis spectrum of a sample of **Ni^{F}** in THF taken before and after the TAS measurement.

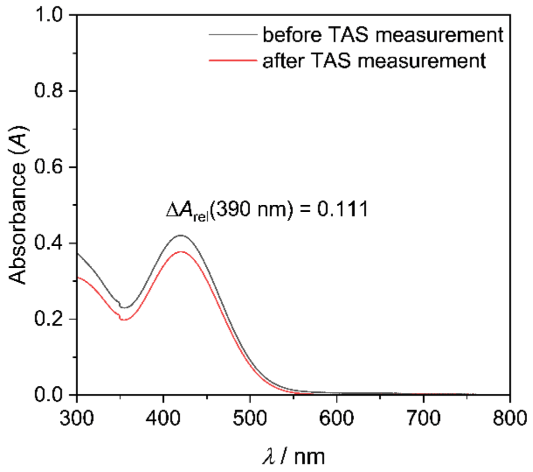

Figure S26: UV-vis spectrum of a sample of **Ni^{Cl}** in THF taken before and after the TAS measurement.

$Ni(CN{-}C_6H_5)_4$ (Ni^H)

A solution of $\mathbf{Ni^H}$ with an optical density of 0.3 was prepared in a 2 mm cuvette. The excitation wavelength was set to 370 nm, and the transient absorption spectra were recorded in the UV probe between 380 and 480 nm. Global analysis was performed using a three-state model ($SAS_1 \rightarrow SAS_2 \rightarrow SAS_3$), where each state corresponds to a species-associated spectrum (SAS). The relative concentrations of the transient species SAS_{1-3} are displayed in Figure S28. Comparison of the global analysis with the kinetic traces attributed to the excited-state absorption (ESA) and ground-state bleach (GSB) are displayed in Figure S30. Additionally, the data was processed via a third party software (MATLAB v.R2024b) and analyzed globally using OPTIMUS (v.3.04) to obtain the decay-associated spectra (DAS).[2] The obtained lifetimes are all well in the error-range of the lifetimes obtained through CARPETVIEW and the SAS are qualitatively similar (Figure S31).

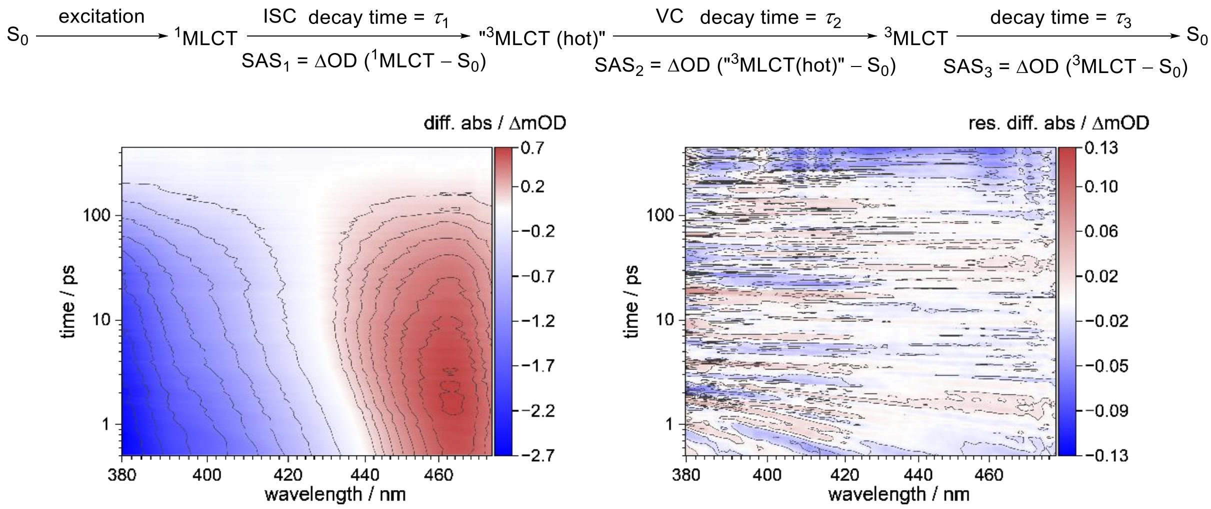

Figure S27: Proposed sequential three-component kinetic model used in the global analysis for the UV-Vis transient absorption spectral data (top) and comparison of the transient absorption data-set (bottom left) with the residual data (bottom right) after the global analysis.

Table S3: Obtained lifetimes of the excited-states through the three-state global-analysis.

Decay in $\mathbf{Ni^H}$	τ_1 (ps)	τ_2 (ps)	$\tau_3 = \tau(^3MLCT)$ (ps)
lifetime	≈1.2±0.4	≈2.8±1.7	99±10

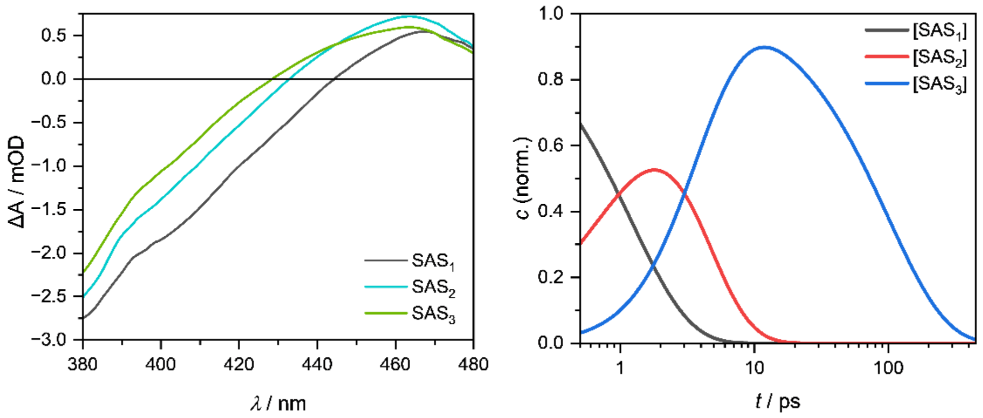

Figure S28: Species-associated spectra (SAS) derived from the global-analysis (left) and the time-resolved concentrations of the transient species S_{1-3} (right).

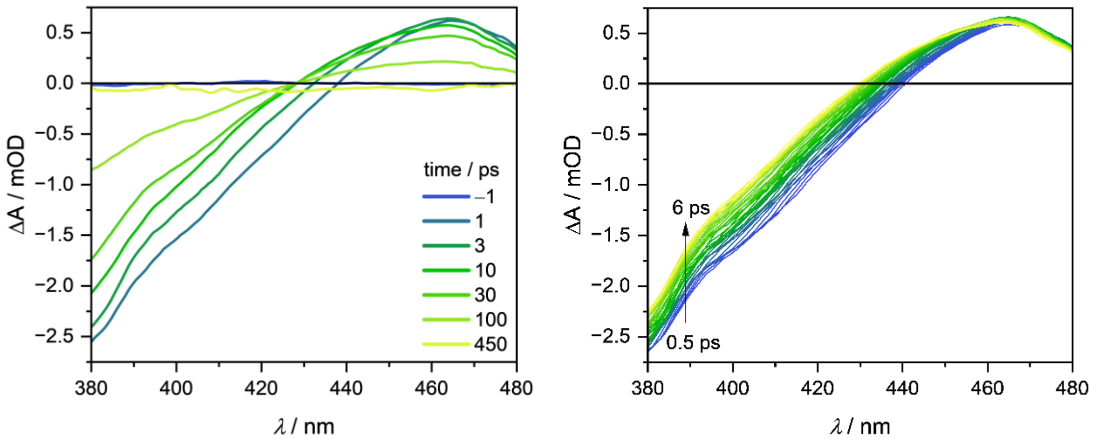

Figure S29: Transient UV-vis absorption spectra of **Ni^{H}** at selected pump-probe delay times (left) and at early delay times after the pump-pulse, highlighting the spectral shifting of the ESA (right).

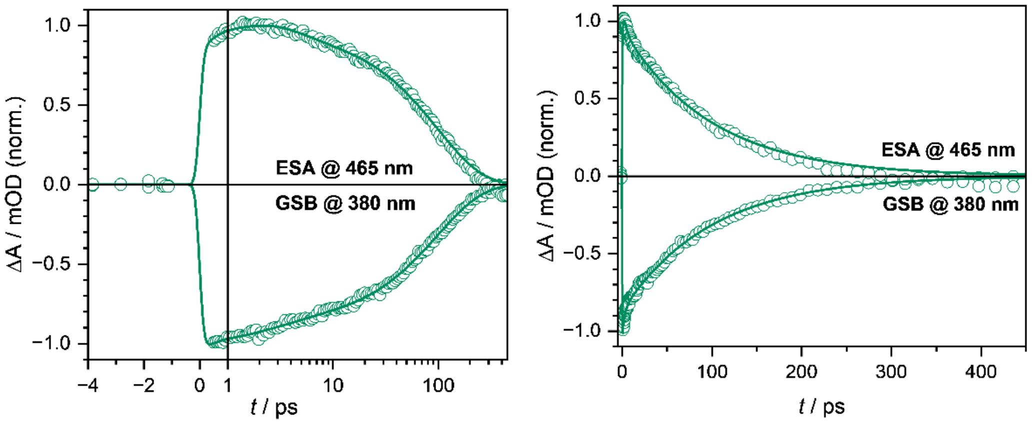

Figure S30: Kinetic traces of **Ni^{H}** at GSB 380 nm & ESA 465 nm (circles) with the global analysis (line) using a semi-logarithmic time-scale (left; break-point @ 1 ps) and a linear time scale (right).

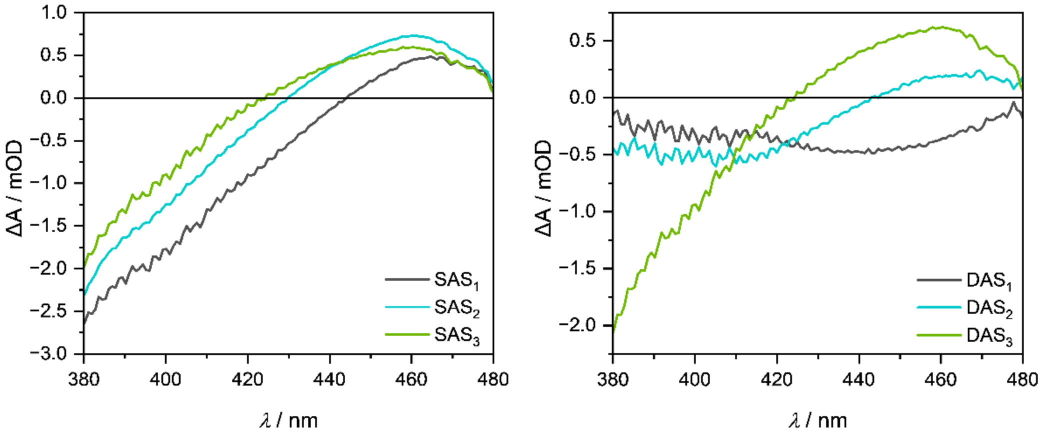

Figure S31: Species-associated spectra (SAS, left) and decay-associated spectra (DAS, right) derived from the global-analysis in OPTIMUS of **Ni^{H}**.

$Ni(CN–C_6F_5)_4$ (Ni^F)

A solution of $\mathbf{Ni^F}$ with an optical density of 0.3 was prepared in a 2 mm cuvette. The excitation wavelength was set to 360 nm, and the transient absorption spectra were recorded in the UV probe between 380 and 480 nm. Global analysis was performed using a three-state model ($SAS_1 \rightarrow SAS_2 \rightarrow SAS_3$), where each state corresponds to a species-associated spectrum (SAS). The relative concentrations of the transient species $SAS_{1–3}$ are displayed in Figure S33. Comparison of the global analysis with the kinetic traces attributed to the excited-state absorption (ESA) and ground-state bleach (GSB) are displayed in Figure S35. Additionally, the data was processed via a third party software (MATLAB v.R2024b) and analyzed globally using OPTIMUS (v.3.04) to obtain the decay-associated spectra (DAS).[2] The obtained lifetimes are all well in the error-range of the lifetimes obtained through CARPETVIEW and the SAS are qualitatively similar (Figure S36).

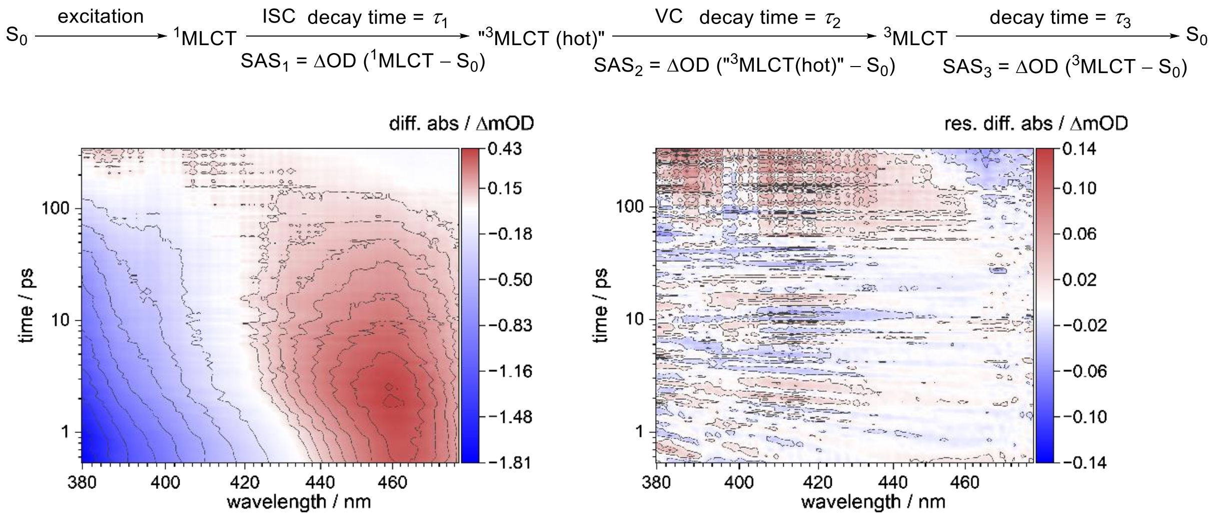

Figure S32: Proposed sequential three-component kinetic model used in the global analysis for the UV-Vis transient absorption spectral data (top) and comparison of the transient absorption data-set (bottom left) with the residual data (bottom right) after the global analysis.

Table S4: Obtained lifetimes of the excited-states in $\mathbf{Ni^F}$ through the three-state global-analysis.

Decay in $\mathbf{Ni^F}$	τ_1 (ps)	τ_2 (ps)	$\tau_3 = \tau(^3MLCT)$ (ps)
lifetime	≈1.1±0.6	≈3.7±2.1	66±15

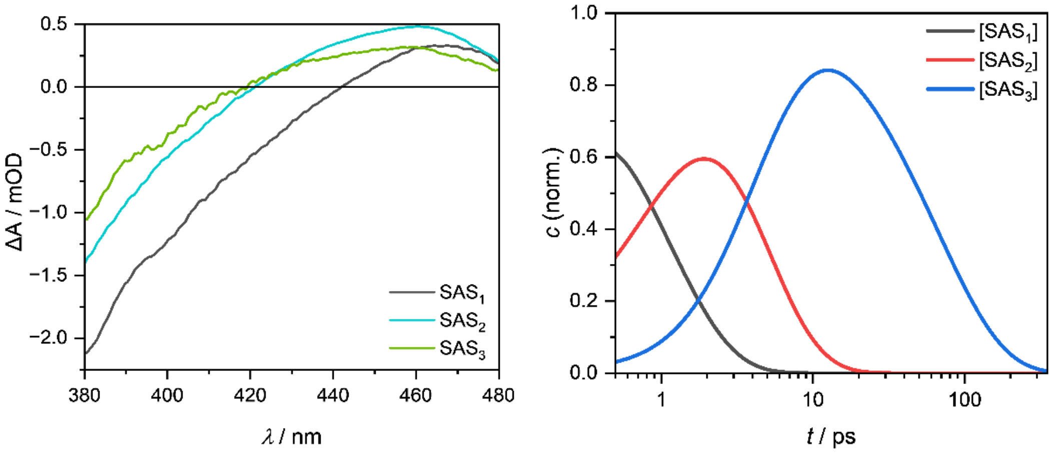

Figure S33: Species-associated spectra (SAS) derived from the global-analysis of $\mathbf{Ni^F}$ (left) and the time-resolved concentrations of the transient species $S_{1–3}$ of $\mathbf{Ni^F}$ (right).

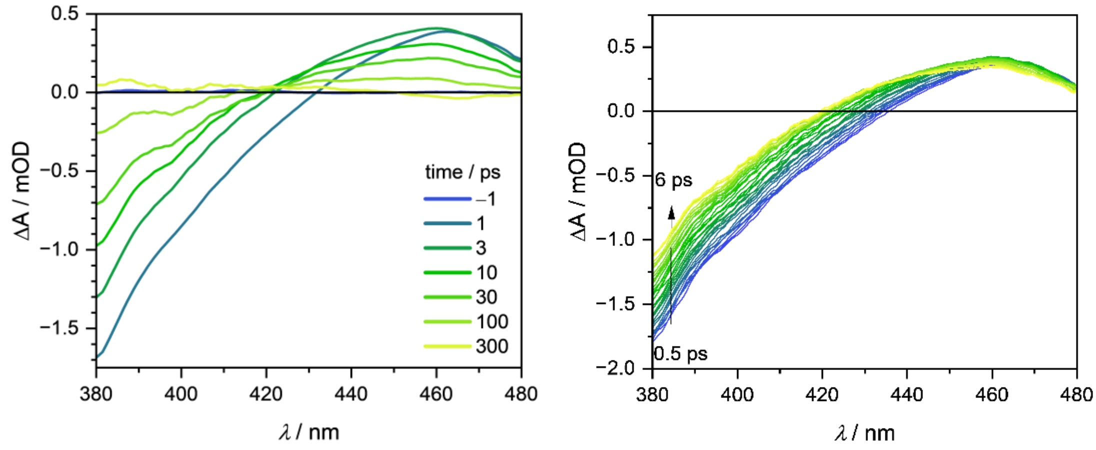

Figure S34: Transient UV-vis absorption spectra of Ni^F at selected pump-probe delay times (left) and at early delay times after the pump-pulse, highlighting the spectral shifting of the ESA (right).

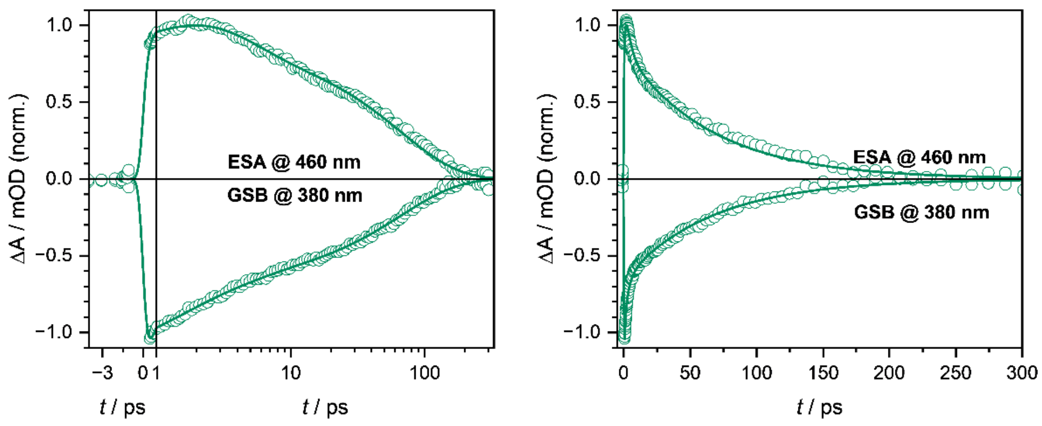

Figure S35: Kinetic traces of Ni^F at GSB 380 nm & ESA 460 nm (circles) with the global analysis (line) using a semi-logarithmic time-scale (left; break-point @ 1 ps) and a linear time scale (right).

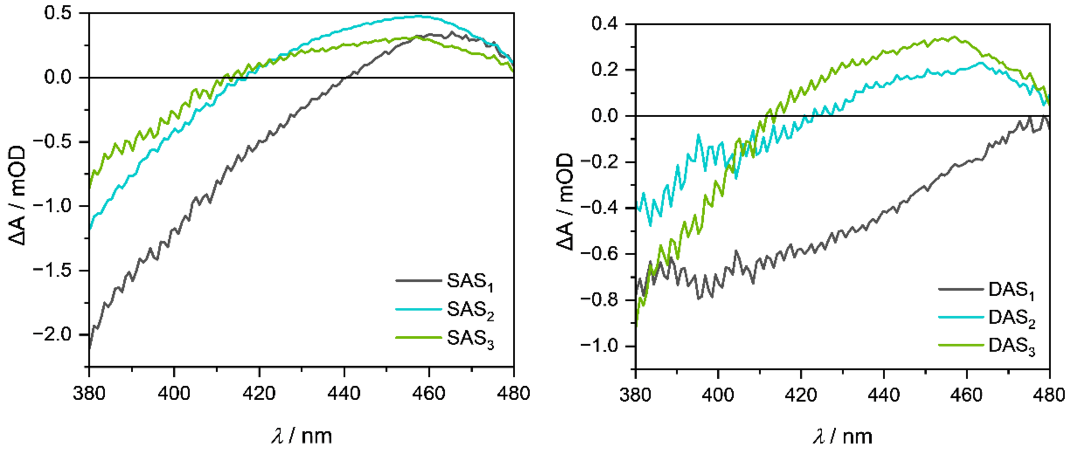

Figure S36: Species-associated spectra (SAS, left) and decay-associated spectra (DAS, right) derived from the global-analysis in Optimus of Ni^F.

Ni(CN–C_6Cl_5)$_4$ (**Ni^{Cl}**)

A solution of **Ni^{Cl}** with an optical density of 0.4 was prepared in a 2 mm cuvette. The excitation wavelength was set to 390 nm, and the transient absorption spectra were recorded in the vis probe between 480 and 780 nm. Global analysis was performed using a three-state model (SAS_1→SAS_2→SAS_3), where each state corresponds to a species-associated spectrum (SAS). The relative concentrations of the transient species $SAS_{1–3}$ are displayed in Figure S38. Comparison of the global analysis with the kinetic traces attributed to the excited-state absorption (ESA) and ground-state bleach (GSB) are displayed in Figure S40. Additionally, the data was processed via a third party software (MATLAB v.R2024b) and analyzed globally using OPTIMUS (v.3.04) to obtain the decay-associated spectra (DAS).[2] The obtained lifetimes are all well in the error-range of the lifetimes obtained through CARPETVIEW and the SAS are qualitatively similar (Figure S41).

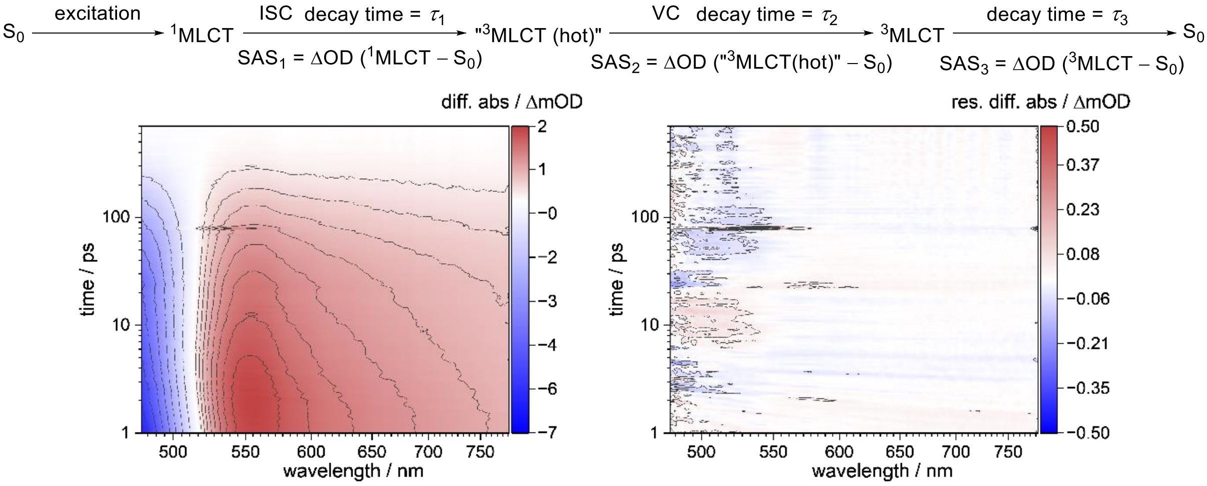

Figure S37: Proposed sequential three-component kinetic model used in the global analysis for the UV-Vis transient absorption spectral data (top) and comparison of the transient absorption data-set (bottom left) with the residual data (bottom right) after the global analysis.

Table S5: Obtained lifetimes of the excited-states in **Ni^{Cl}** through the three-state global-analysis.

Decay in **Ni^{Cl}**	τ_1 (ps)	τ_2 (ps)	τ_3 = τ(^{3}MLCT) (ps)
lifetime	≈1.5±0.5	≈3.7±1.4	141±10

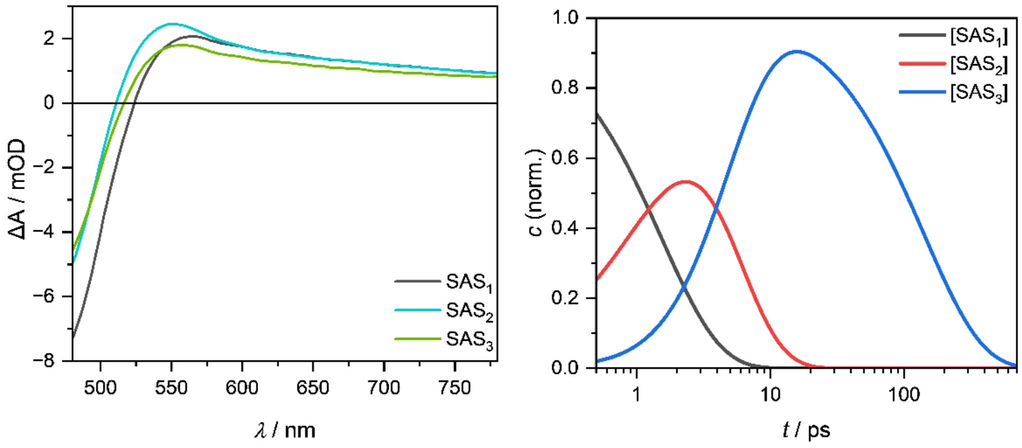

Figure S38: Proposed sequential three-component kinetic model used in the global analysis for the UV-Vis transient absorption spectral data (top), species-associated spectra (SAS) derived from the global analysis of **Ni^{Cl}** (left) and the time-resolved concentrations of the transient species $S_{1–3}$ of **Ni^{Cl}** (right).

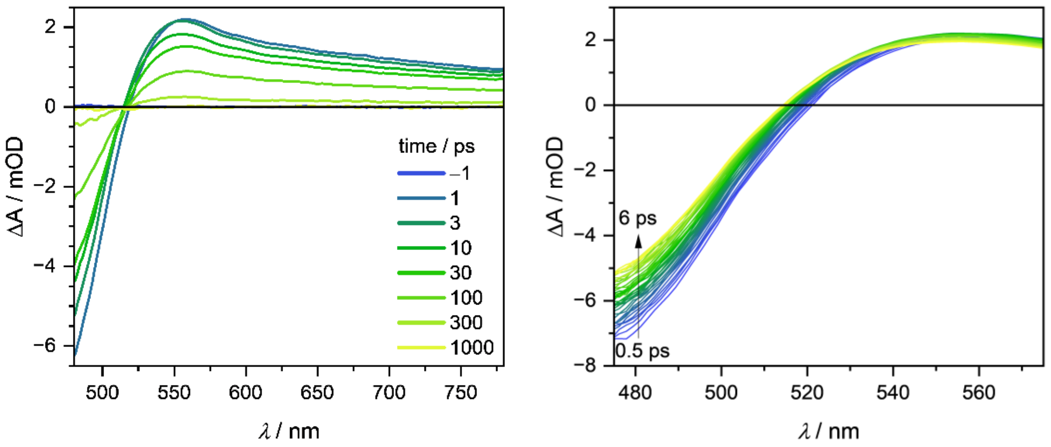

Figure S39: Transient UV-vis absorption spectra of **Ni^{Cl}** at selected pump-probe delay times (left) and at early delay times after the pump-pulse in the sector between 475–575 nm, highlighting the spectral shifting of the ESA (right).

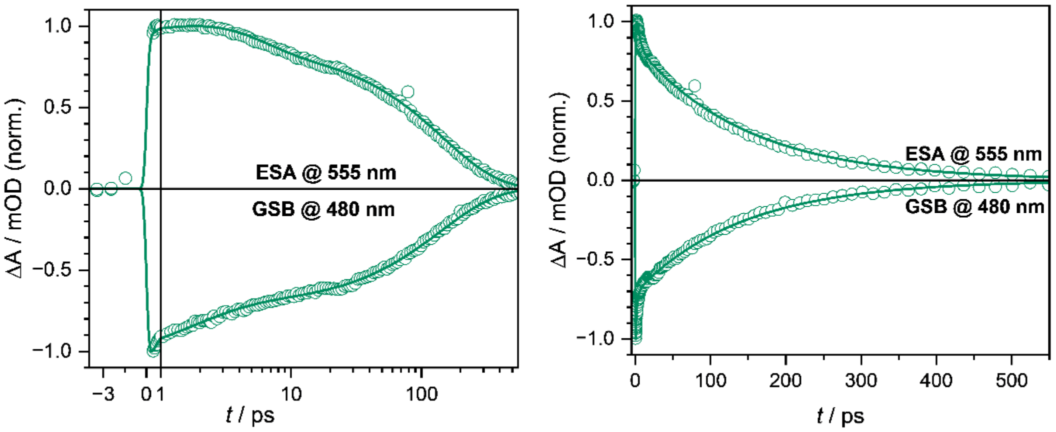

Figure S40: Kinetic traces of **Ni^{Cl}** at GSB 480 nm & ESA 555 nm (circles) with the global analysis (line) using a logarithmic (left) and a linear time scale (right).

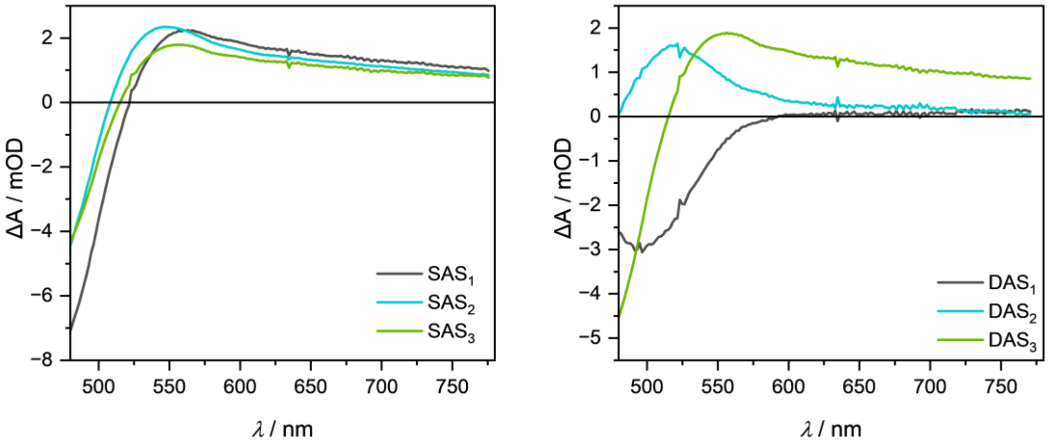

Figure S41: Species-associated spectra (SAS, left) and decay-associated spectra (DAS, right) derived from the global-analysis in OPTIMUS of **Ni^{Cl}**.

6. Optimized Structures

Ni(CN–C_6H_5)$_4$ (neutral, singlet)

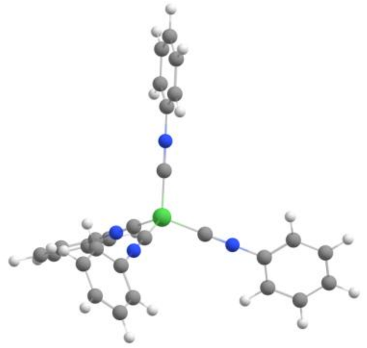

Ni	0.000000	0.000000	0.000000
C	0.000000	1.502801	1.081803
N	-0.003284	2.453235	1.775379
C	-0.006509	3.559230	2.585048
C	-0.708756	4.704239	2.199581
H	-1.244387	4.701677	1.260568
C	-0.707053	5.818955	3.024032
H	-1.251867	6.704247	2.722927
C	-0.012461	5.804252	4.229042
H	-0.014825	6.677116	4.868179
C	0.685102	4.662340	4.608711
H	1.227580	4.644455	5.545010
C	0.692742	3.539898	3.794831
H	1.230786	2.645765	4.077248
C	-0.000000	-1.502801	1.081803
N	0.003284	-2.453235	1.775379
C	0.006509	-3.559230	2.585048
C	0.708756	-4.704239	2.199581
H	1.244387	-4.701677	1.260568
C	0.707053	-5.818955	3.024032
H	1.251867	-6.704247	2.722927
C	0.012461	-5.804252	4.229042
H	0.014825	-6.677116	4.868179
C	-0.685102	-4.662340	4.608711
H	-1.227580	-4.644455	5.545010
C	-0.692742	-3.539898	3.794831
H	-1.230786	-2.645765	4.077248
C	1.502801	-0.000000	-1.081803
N	2.453235	0.003284	-1.775379
C	3.559230	0.006509	-2.585048
C	4.704239	0.708756	-2.199581
H	4.701677	1.244387	-1.260568
C	5.818955	0.707053	-3.024032
H	6.704247	1.251867	-2.722927
C	5.804252	0.012461	-4.229042
H	6.677116	0.014825	-4.868179
C	4.662340	-0.685102	-4.608711
H	4.644455	-1.227580	-5.545010
C	3.539898	-0.692742	-3.794831
H	2.645765	-1.230786	-4.077248
C	-1.502801	0.000000	-1.081803
N	-2.453235	-0.003284	-1.775379
C	-3.559230	-0.006509	-2.585048
C	-4.704239	-0.708756	-2.199581
H	-4.701677	-1.244387	-1.260568
C	-5.818955	-0.707053	-3.024032
H	-6.704247	-1.251867	-2.722927
C	-5.804252	-0.012461	-4.229042
H	-6.677116	-0.014825	-4.868179
C	-4.662340	0.685102	-4.608711
H	-4.644455	1.227580	-5.545010
C	-3.539898	0.692742	-3.794831
H	-2.645765	1.230786	-4.077248

$Ni(CN–C_6F_5)_4$ (neutral, singlet)

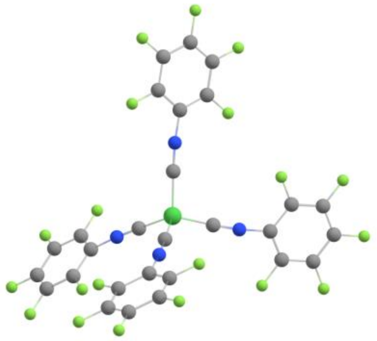

Ni	0.000000	-0.000000	-0.000560
C	-0.401456	-1.399715	-1.132205
N	-0.650446	-2.260363	-1.896836
C	-0.886890	-3.161310	-2.882122
C	-1.468251	-4.401538	-2.600379
F	-1.794982	-4.704097	-1.346577
C	-1.710183	-5.321316	-3.607055
F	-2.264803	-6.497854	-3.321219
C	-1.373444	-5.015733	-4.919409
F	-1.604769	-5.897363	-5.887659
C	-0.794972	-3.789210	-5.220760
F	-0.472979	-3.499494	-6.479958
C	-0.554837	-2.873041	-4.210720
F	0.000000	-1.701560	-4.508340
C	0.401456	1.399715	-1.132205
N	0.650446	2.260363	-1.896836
C	0.886890	3.161310	-2.882122
C	1.468251	4.401538	-2.600379
F	1.794982	4.704097	-1.346577
C	1.710183	5.321316	-3.607055
F	2.264803	6.497854	-3.321219
C	1.373444	5.015733	-4.919409
F	1.604769	5.897363	-5.887659
C	0.794972	3.789210	-5.220760
F	0.472979	3.499494	-6.479958
C	0.554837	2.873041	-4.210720
F	-0.000000	1.701560	-4.508340
C	1.399590	-0.401407	1.131224
N	2.260149	-0.650302	1.895990
C	3.160637	-0.886511	2.881758
C	4.401545	-1.466677	2.600552
F	4.705216	-1.792526	1.346791
C	5.320855	-1.708336	3.607723
F	6.498052	-2.261820	3.322397
C	5.014115	-1.372508	4.920040
F	5.895298	-1.603564	5.888761
C	3.786914	-0.795197	5.220857
F	3.496101	-0.474047	6.480016
C	2.871219	-0.555326	4.210325
F	1.699090	-0.001581	4.507430
C	-1.399590	0.401407	1.131224
N	-2.260149	0.650302	1.895990
C	-3.160637	0.886511	2.881758
C	-4.401545	1.466677	2.600552
F	-4.705216	1.792526	1.346791
C	-5.320855	1.708336	3.607723
F	-6.498052	2.261820	3.322397
C	-5.014115	1.372508	4.920040
F	-5.895298	1.603564	5.888761
C	-3.786914	0.795197	5.220857
F	-3.496101	0.474047	6.480016
C	-2.871219	0.555326	4.210325
F	-1.699090	0.001581	4.507430

Ni(CN–C_6Cl_5)$_4$ (neutral, singlet)

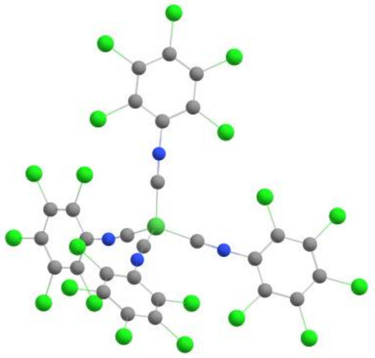

Ni	0.000276	-0.298918	0.000251
Cl	0.844115	4.238770	1.583860
Cl	6.902534	-2.672082	-2.085473
Cl	2.747053	6.297536	3.004521
Cl	5.461978	5.318620	4.201756
Cl	4.514837	-1.211072	-0.654218
Cl	4.336377	0.280644	2.561482
Cl	3.360267	-5.347532	-5.181152
Cl	6.269800	2.308118	3.983989
Cl	6.310776	-4.742648	-4.349407
Cl	1.001889	-3.859379	-3.729266
Cl	-0.844648	4.237295	-1.585925
Cl	-6.902415	-2.669653	2.085891
Cl	-2.747661	6.294828	-3.008274
Cl	-5.461766	5.314510	-4.206222
Cl	-4.513969	-1.210608	0.653885
Cl	-4.335214	0.277612	-2.563291
Cl	-3.361773	-5.340924	5.187032
Cl	-6.268703	2.303840	-3.987484
Cl	-6.311846	-4.737150	4.352938
Cl	-1.002633	-3.854740	3.734367
N	1.876188	-1.982603	-1.659874
N	1.878425	1.500411	1.537591
N	-1.875847	-1.980758	1.661985
N	-1.878074	1.498613	-1.538935
C	3.923329	1.945371	2.695613
C	5.011514	-3.934694	-3.561248
C	4.774794	2.851249	3.326500
C	2.637926	-3.551821	-3.296407
C	2.344538	3.733599	2.255765
C	4.411514	4.199843	3.423276
C	2.703540	2.379903	2.154741
C	3.195125	4.639776	2.887387
C	3.690140	-4.206857	-3.935002
C	5.278144	-3.006788	-2.547145
C	4.226104	-2.352358	-1.908132
C	1.112234	-1.343149	-1.031518
C	1.130069	0.788105	0.972329
C	2.898494	-2.620994	-2.280041
C	-3.922647	1.942414	-2.697982
C	-5.012170	-3.930273	3.564359
C	-4.774146	2.847742	-3.329612
C	-2.638443	-3.547765	3.300238
C	-2.344619	3.731344	-2.258251
C	-4.411263	4.196411	-3.426821
C	-2.703222	2.377574	-2.156797
C	-3.195240	4.636972	-2.890616
C	-3.690992	-4.201933	3.939169
C	-5.278266	-3.003740	2.548860
C	-4.225892	-2.350183	1.909507
C	-1.111741	-1.342039	1.033063
C	-1.129601	0.786991	-0.972965
C	-2.898476	-2.618314	2.282477

Ni(CO)$_4$ (neutral, singlet)

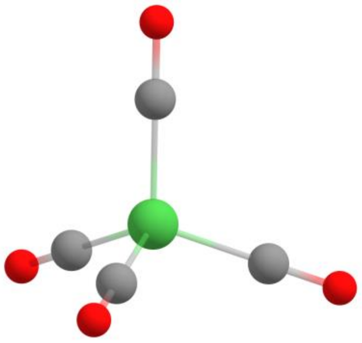

Ni	0.000000	-0.000000	-0.000620
C	-0.000000	1.502798	1.062717
O	-0.000187	2.428827	1.718152
C	0.000000	-1.502798	1.062717
O	0.000187	-2.428827	1.718152
C	1.503437	-0.000130	-1.062779
O	2.430322	-0.000111	-1.717021
C	-1.503437	0.000130	-1.062779
O	-2.430322	0.000111	-1.717021

CN–C_6H_5 (neutral, singlet)

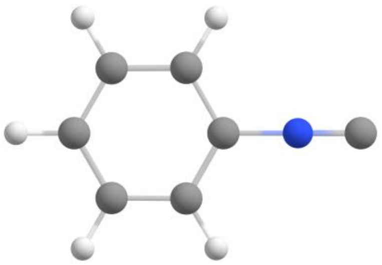

C	0.000000	0.631993	-0.000000
C	1.211855	-0.055944	0.000000
C	1.203977	-1.442884	0.000000
C	-0.000002	-2.138479	0.000000
C	-1.203980	-1.442882	0.000000
C	-1.211856	-0.055941	0.000000
N	0.000002	2.015164	-0.000000
H	2.138476	0.500132	-0.000000
H	2.142169	-1.981263	0.000000
H	-0.000003	-3.220184	0.000000
H	-2.142173	-1.981258	0.000000
H	-2.138476	0.500136	-0.000000
C	0.000005	3.183519	-0.000000

CN–C6F5 (neutral, singlet)

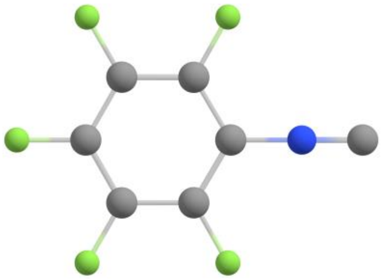

C	-0.000001	-1.206566	-0.000000
C	-1.202024	-0.495940	-0.000000
C	-1.204459	0.889573	0.000000
C	-0.000001	1.581717	0.000000
C	1.204459	0.889572	0.000000
C	1.202024	-0.495939	-0.000000
N	0.000001	-2.573023	-0.000000
F	-2.355872	-1.151859	-0.000000
F	-2.354121	1.558231	0.000000
F	0.000000	2.909263	0.000000
F	2.354119	1.558233	0.000000
F	2.355871	-1.151860	-0.000000
C	0.000002	-3.743571	-0.000000

CN–C_6Cl_5 (neutral, singlet)

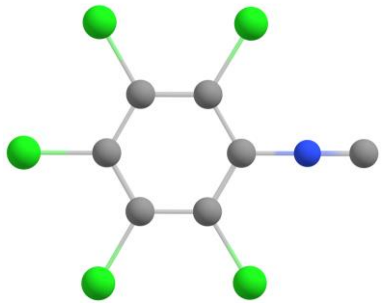

C	-0.000000	-1.462956	0.000000
C	-1.214328	-0.764800	-0.000000
C	-1.214265	0.630607	-0.000000
C	-0.000000	1.327281	-0.000000
C	1.214264	0.630607	0.000000
C	1.214328	-0.764800	0.000000
N	-0.000000	-2.830091	0.000000
Cl	-2.685097	-1.651720	-0.000000
Cl	-2.704776	1.488384	-0.000000
Cl	-0.000001	3.046600	-0.000000
Cl	2.704777	1.488383	0.000000
Cl	2.685097	-1.651720	0.000000
C	0.000000	-4.000626	0.000000

CO (neutral, singlet)

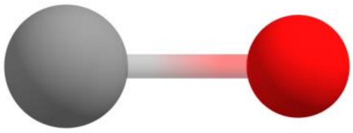

C	-0.000000	0.000000	-0.642783
O	0.000000	-0.000000	0.482087

7. TD-DFT Calculations

Table S6: Calculated excited singlet states (S_1–S_{12}) of **Ni^{H}** obtained from TDDFT calculations at the ground-state optimized S_0 geometry. For each excitation, the dominant hole–electron natural transition orbital (NTO) pairs contributing more than 2.0% to the total transition density are shown, visualized at an isodensity value of 0.03 e B^{-3}. Excitation energies (E_{exc}) and oscillator strengths (f) are listed. NTO phases are chosen arbitrarily. Geometry optimizations and excited-state calculations were performed at the B3LYP(D3BJ)/def2-TZVP level of theory.

State	Excitation energy (E_{exc})	Natural transition orbitals hole → electron			Oscillator strength (f)
S_1	3.0085 eV<br>403.29 nm	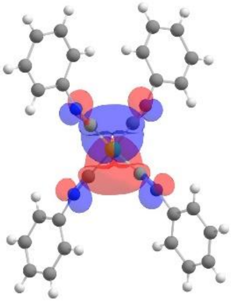<br>120	→ 6.3%	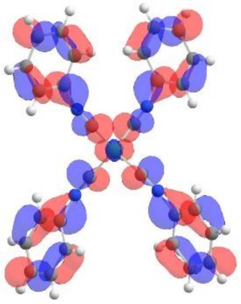<br>125	0
		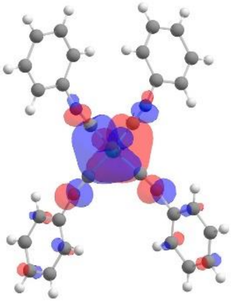<br>121	→ 44.1%	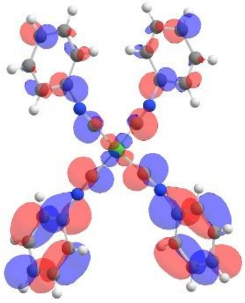<br>124	
		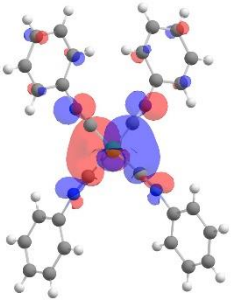<br>122	→ 44.1%	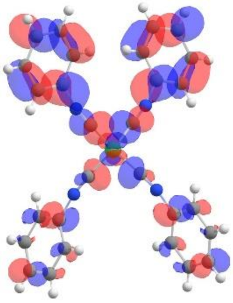<br>123	
S_2	3.0743 eV<br>403.29 nm	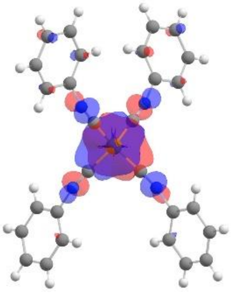<br>120	→ 3.5%	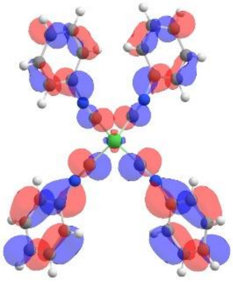<br>125	0.0381

		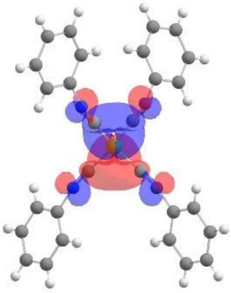 121	→ 16.1%	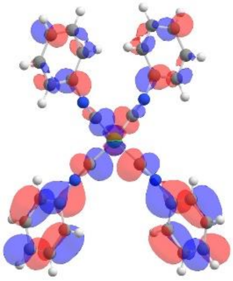 124	
		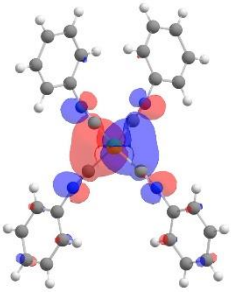 122	→ 74.9%	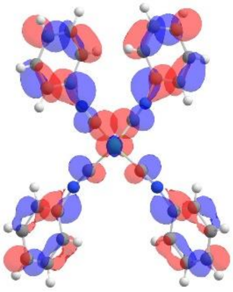 123	
S_3	3.0743 eV<br>403.29 nm	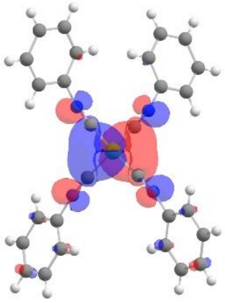 120	→ 3.5%	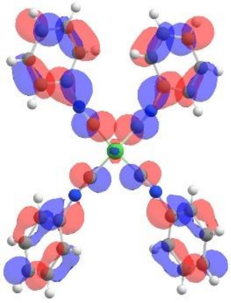 125	0.0381
		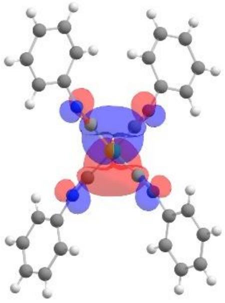 121	→ 16.1%	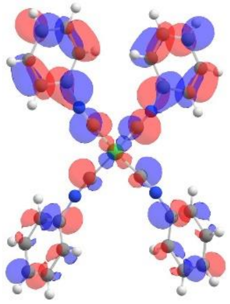 124	
		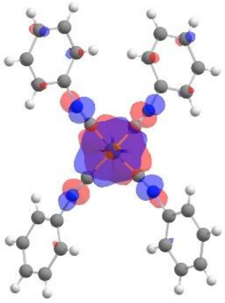 122	→ 74.9%	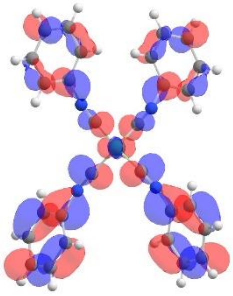 123	

S_4	3.0946 eV<br>400.64 nm	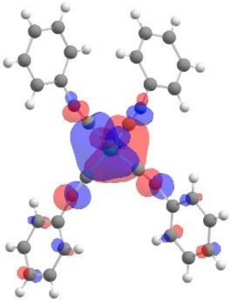<br>120	→<br>5.5%	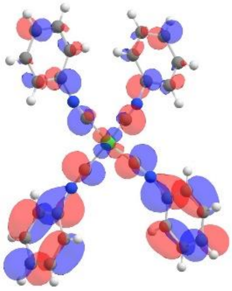<br>125	0
		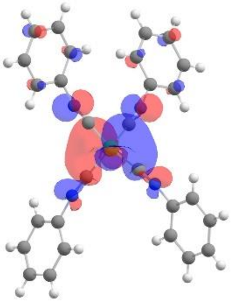<br>121	→<br>5.5%	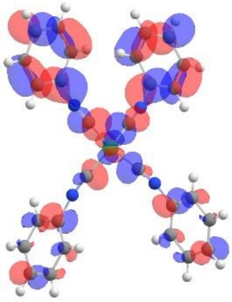<br>124	
		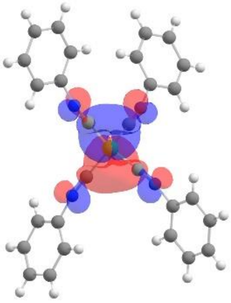<br>122	→<br>87.9%	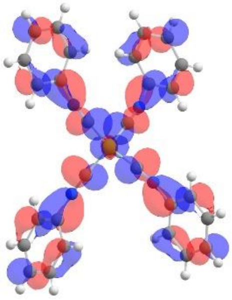<br>123	
S_5	3.1113 eV<br>398.49 nm	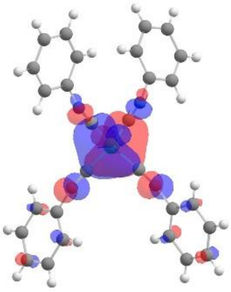<br>121	→<br>47.0%	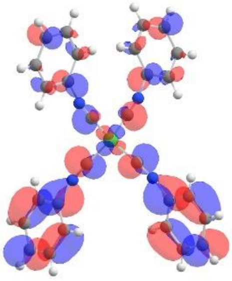<br>124	0.0033
		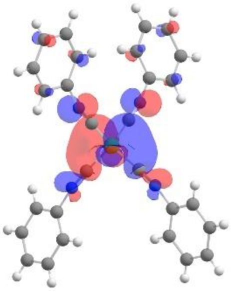<br>122	→<br>47.0%	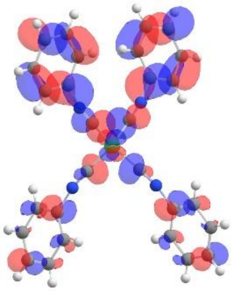<br>123	

S_6	3.1628 eV<br>392.01 nm	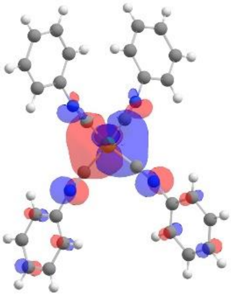<br>120	→<br>2.6%	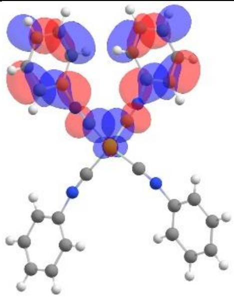<br>125	0.0069
		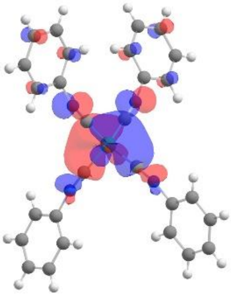<br>121	→<br>21.2%	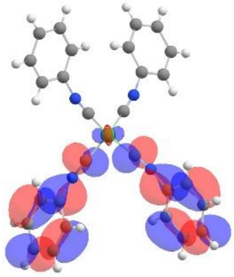<br>124	
		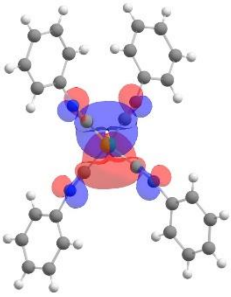<br>122	→<br>71.1%	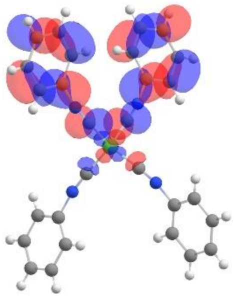<br>123	
S_7	3.1628 eV<br>392.01 nm	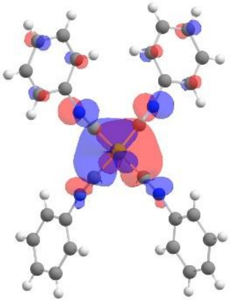<br>120	→<br>2.6%	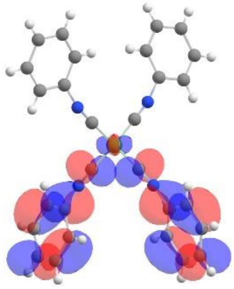<br>125	0.0069
		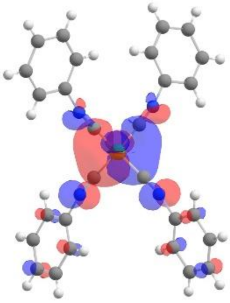<br>121	→<br>21.2%	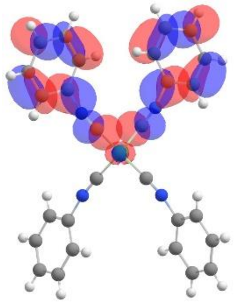<br>124	

		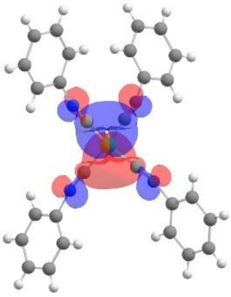 122	→ 71.1%	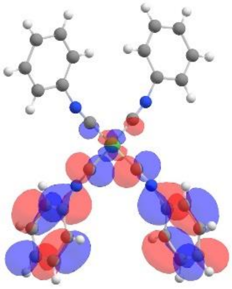 123	
S_8	3.2746 eV 278.62 nm	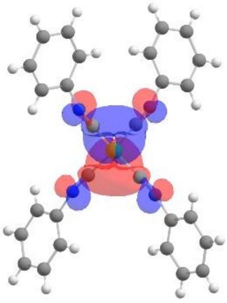 120	→ 7.1%	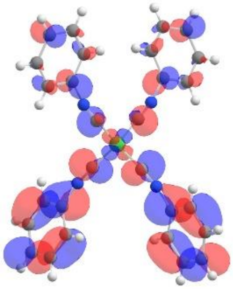 125	0.1663
		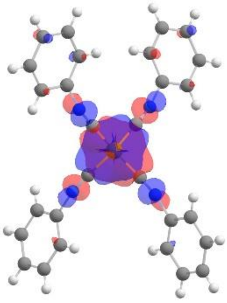 121	→ 9.7%	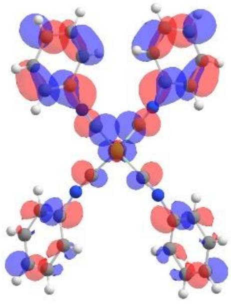 124	
		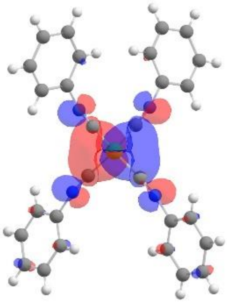 122	→ 79.6%	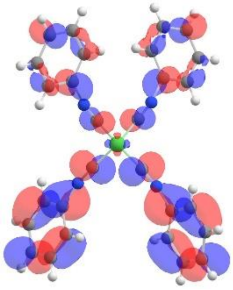 123	
S_9	3.2746 eV 378.62 nm	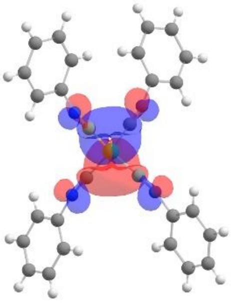 120	→ 7.1%	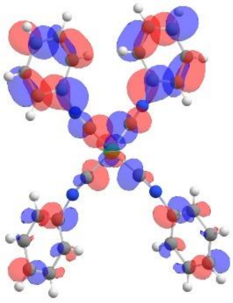 125	0.1663

		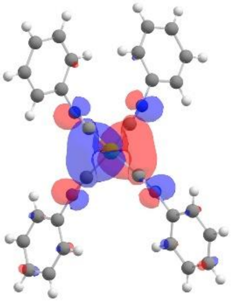<br>121	→ 9.7%	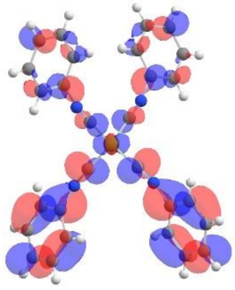<br>124	
		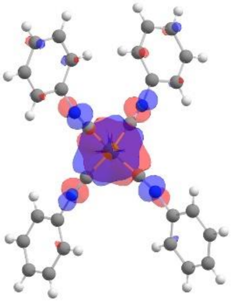<br>122	→ 79.6%	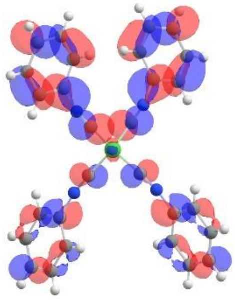<br>123	
S_{10}	3.3128 eV<br>374.25 nm	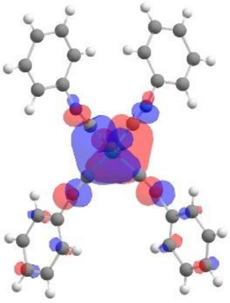<br>120	→ 16.9%	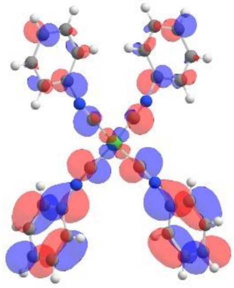<br>125	0.0909
		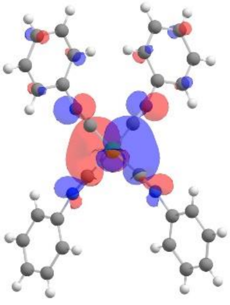<br>121	→ 16.9%	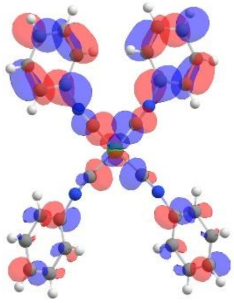<br>124	
		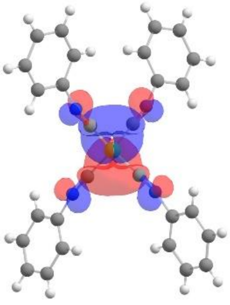<br>122	→ 64.5%	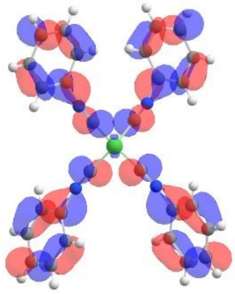<br>123	

S_{11}	3.3481 eV<br>370.31 nm	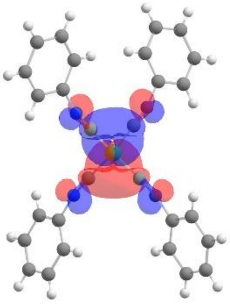<br>120	→<br>32.3%	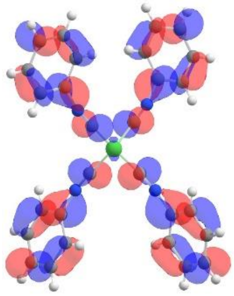<br>125	0.4414
		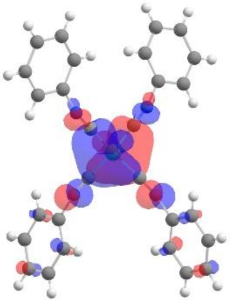<br>121	→<br>32.7%	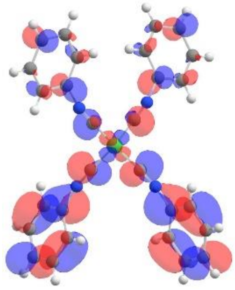<br>124	
		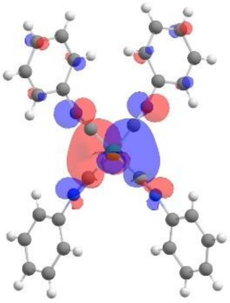<br>122	→<br>32.7%	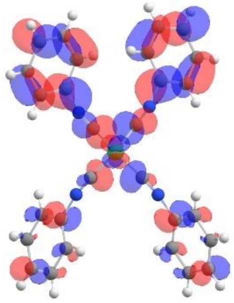<br>123	
S_{12}	3.4162 eV<br>362.93 nm	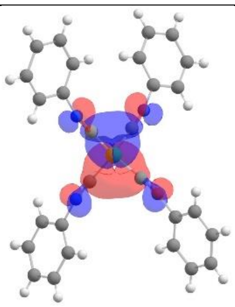<br>120	→<br>11.5%	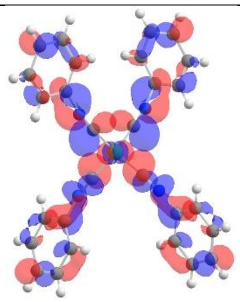<br>125	0
		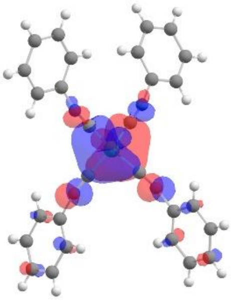<br>121	→<br>40.1%	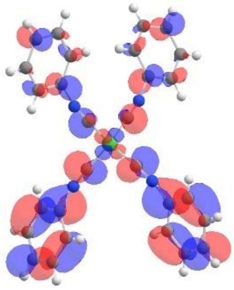<br>124	

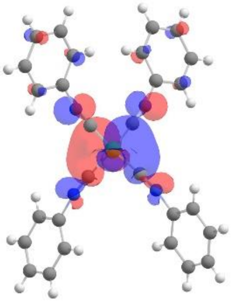

→

40.1%

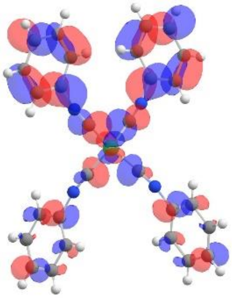

122

123

Table S7: Calculated excited singlet states (S_1–S_{12}) of **Ni^F^** obtained from TDDFT calculations at the ground-state optimized S_0 geometry. For each excitation, the dominant hole–electron natural transition orbital (NTO) pairs contributing more than 2.0% to the total transition density are shown, visualized at an isodensity value of 0.03 *e* B^{-3}. Excitation energies (E_{exc}) and oscillator strengths (*f*) are listed. NTO phases are chosen arbitrarily. Geometry optimizations and excited-state calculations were performed at the B3LYP(D3BJ)/def2-TZVP level of theory.

State	Excitation energy (E_{exc})	Natural transition orbitals hole → electron			Oscillator strength (*f*)
S_1	2.8990 eV<br>427.68 nm	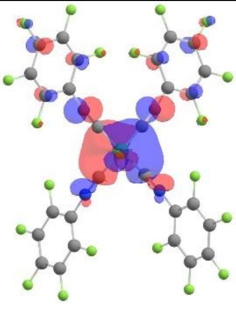<br>201	→ 47.0%	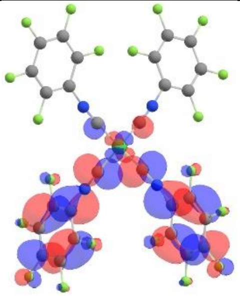<br>204	0
		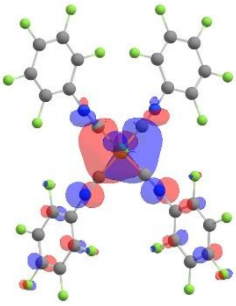<br>202	→ 47.2%	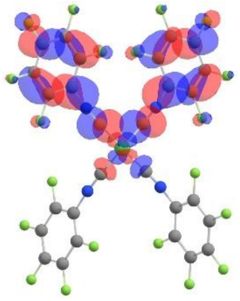<br>203	
S_2	3.0116 eV<br>411.69 nm	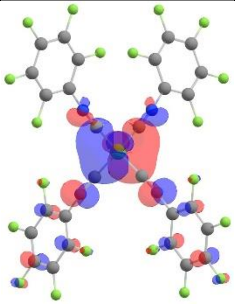<br>201	→ 47.2%	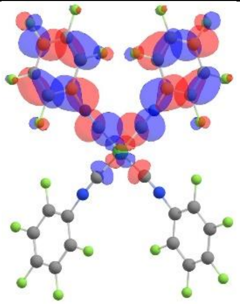<br>204	0.014
		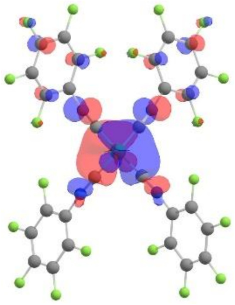<br>202	→ 47.2%	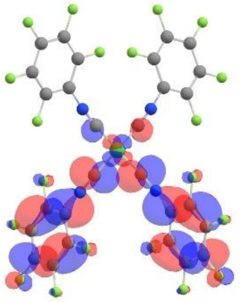<br>203	

S_3	3.0164 eV<br>411.03 nm	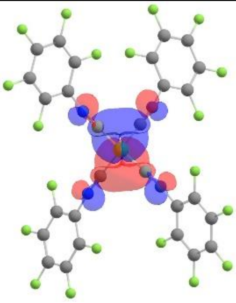<br>200	→<br>4.8%	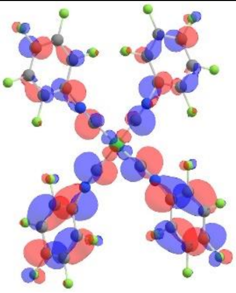<br>205	0.0492
		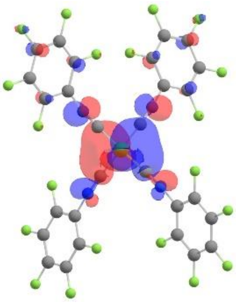<br>201	→<br>8.2%	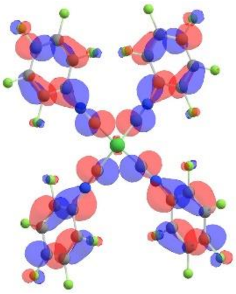<br>204	
		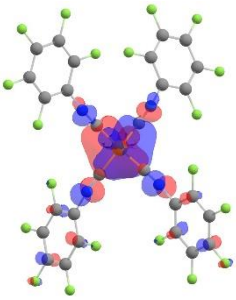<br>202	→<br>83.3%	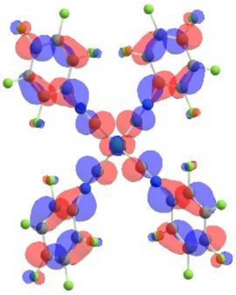<br>203	
S_4	3.0166 eV<br>411.00 nm	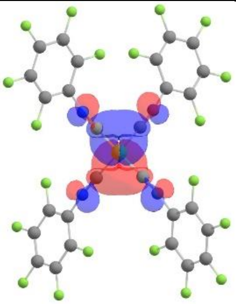<br>200	→<br>4.8%	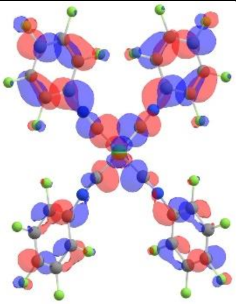<br>205	0.0492
		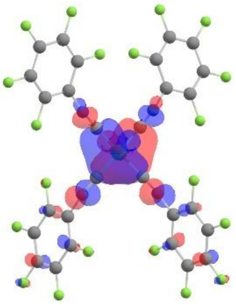<br>201	→<br>8.3%	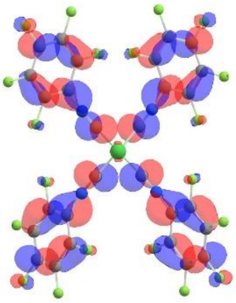<br>204	

		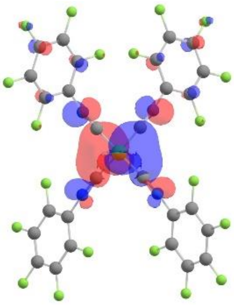 202	→ 83.2%	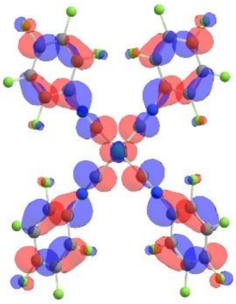 203	
S_5	3.1074<br>399.00 nm	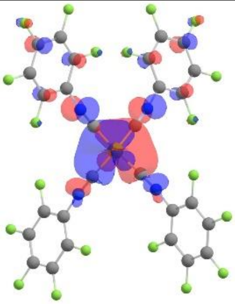 200	→ 2.9%	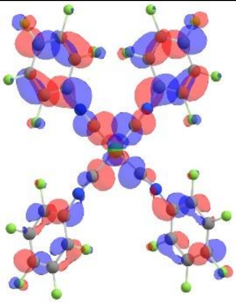 205	0
		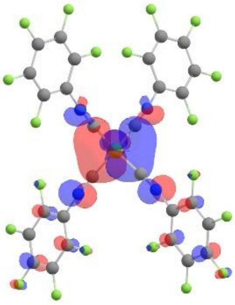 201	→ 2.9%	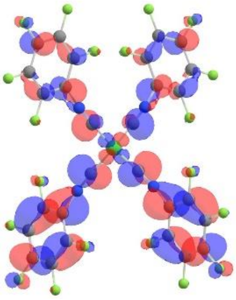 204	
		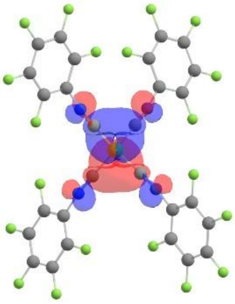 202	→ 92.8%	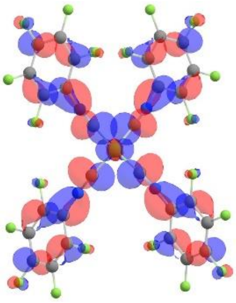 203	
S_6	3.1376 eV<br>395.16 nm	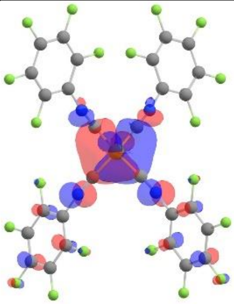 201	→ 20.9%	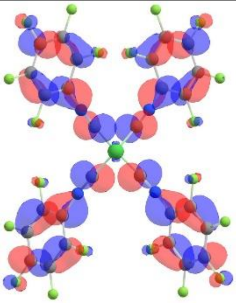 204	0.0091

		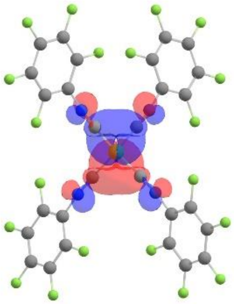 202	→ 72.9%	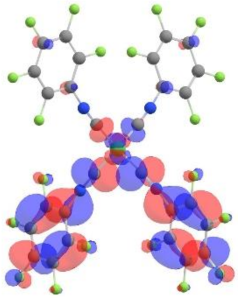 203	
S_7	3.1377 eV 395.14 nm	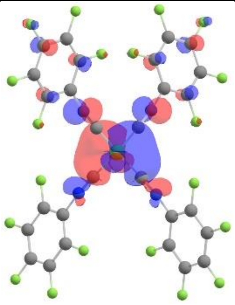 201	→ 20.7%	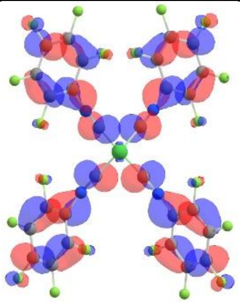 204	0.0090
		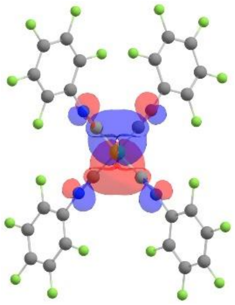 202	→ 73.1%	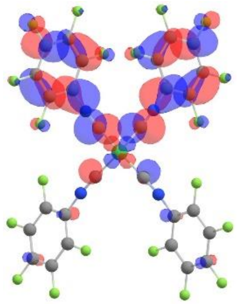 203	
S_8	3.2135 eV 385.82 nm	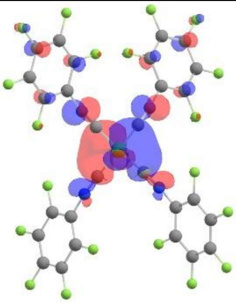 200	→ 9.5%	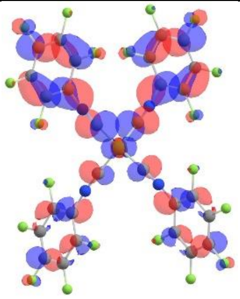 205	0.1744
		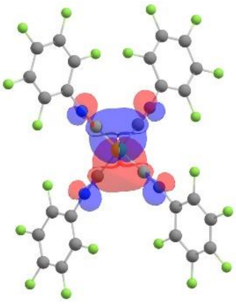 201	→ 15.6%	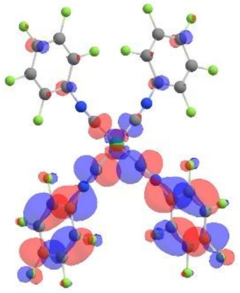 204	

		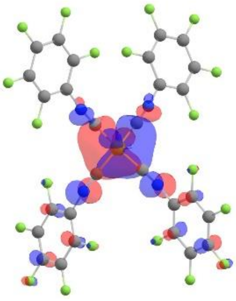<br>202	→ 70.9%	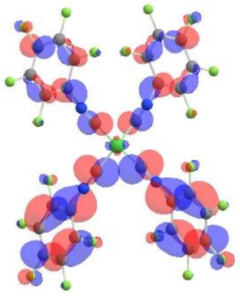<br>203	
S_9	3.2135 eV<br>385.82 nm	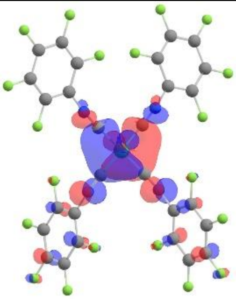<br>200	→ 9.5%	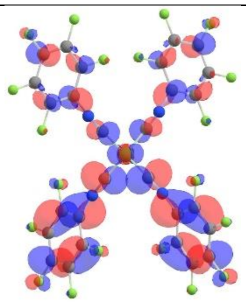<br>205	0.1747
		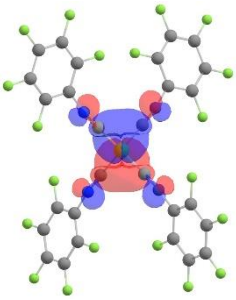<br>201	→ 15.4%	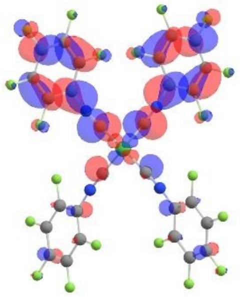<br>204	
		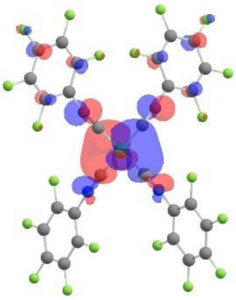<br>202	→ 71.2%	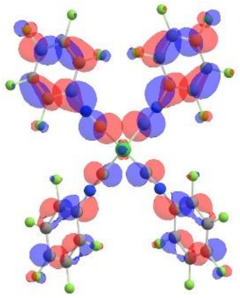<br>203	
S_{10}	3.2665 eV<br>379.56 nm	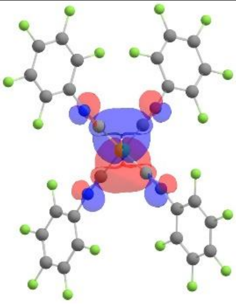<br>200	→ 2.3%	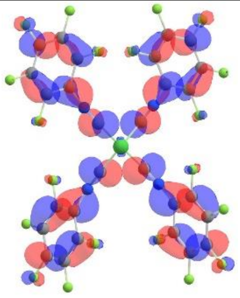<br>205	0.5013

		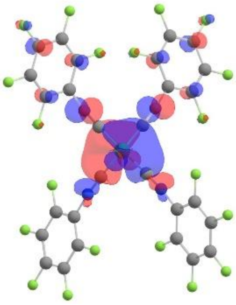<br>201	→ 47.2%	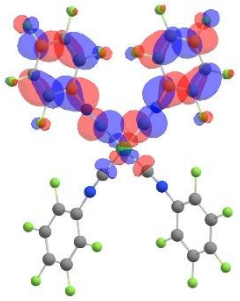<br>204	
		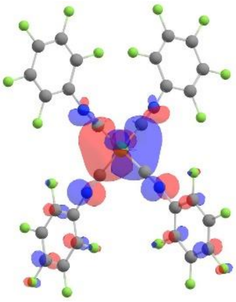<br>202	→ 47.5%	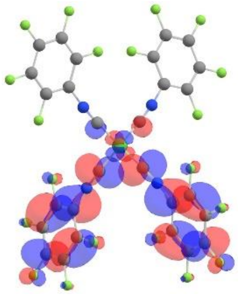<br>203	
S_{11}	3.3398 eV<br>371.23 nm	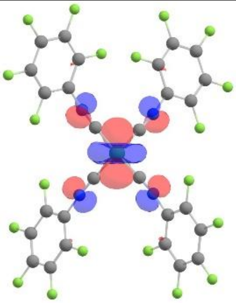<br>199	→ 3.0%	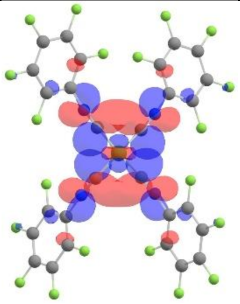<br>206	0
		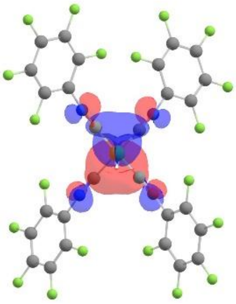<br>200	→ 12.7%	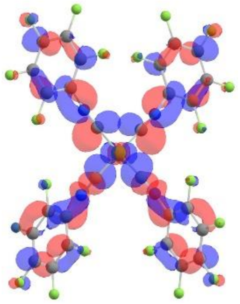<br>205	
		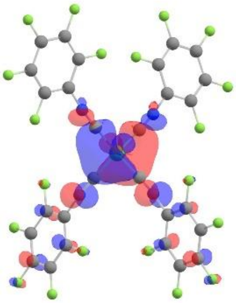<br>201	→ 39.5%	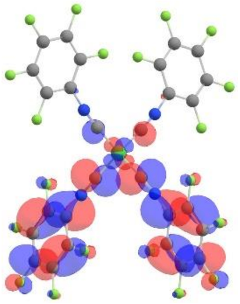<br>204	

		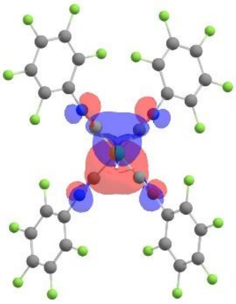 202	→ 40.1%	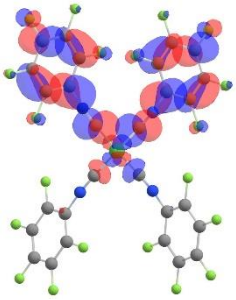 203	
S_{12}	3.3590 eV 369.11 nm	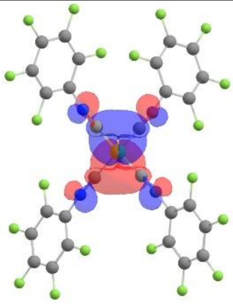 202	→ 95.3%	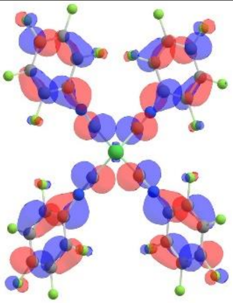 203	0.0525

Table S8: Calculated excited singlet states (S_1–S_{12}) of **Ni^{Cl}** obtained from TDDFT calculations at the ground-state optimized S_0 geometry. For each excitation, the dominant hole–electron natural transition orbital (NTO) pairs contributing more than 2.0% to the total transition density are shown, visualized at an isodensity value of 0.03 *e* B^{-3}. Excitation energies (E_{exc}) and oscillator strengths (*f*) are listed. NTO phases are chosen arbitrarily. Geometry optimizations and excited-state calculations were performed at the B3LYP(D3BJ)/def2-TZVP level of theory.

State	Excitation energy (E_{exc})	Natural transition orbitals hole → electron			Oscillator strength (*f*)
S_1	2.6901 eV<br>460.89 nm	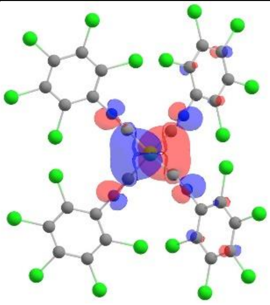<br>281	→ 36.4%	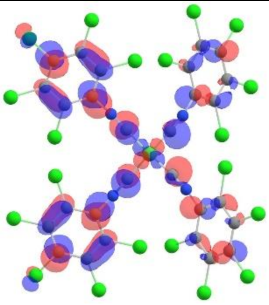<br>284	0.0285
		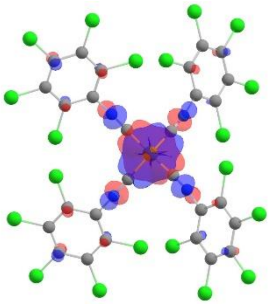<br>282	→ 59.4%	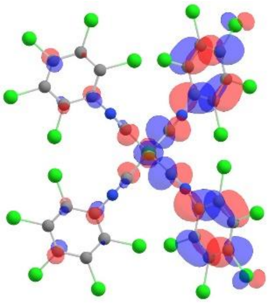<br>283	
S_2	2.7433 eV<br>451.95 nm	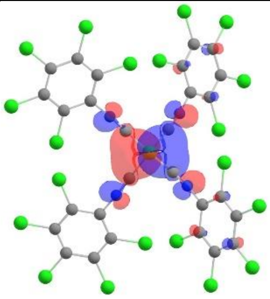<br>281	→ 4.2%	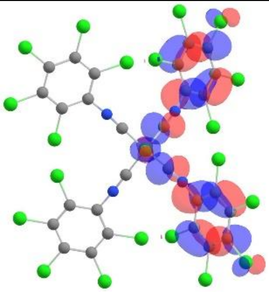<br>284	0.1029
		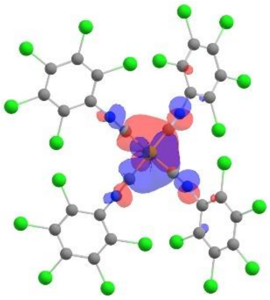<br>282	→ 89.8%	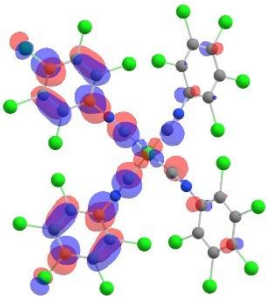<br>283	

S_3	2.7640 eV<br>448.56 nm	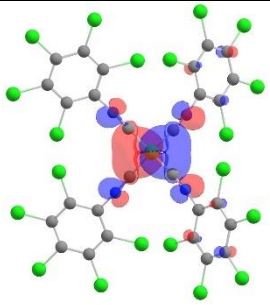<br>281	→ 3.1%	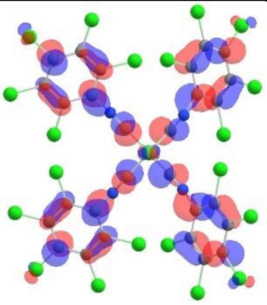<br>284	0.1029
		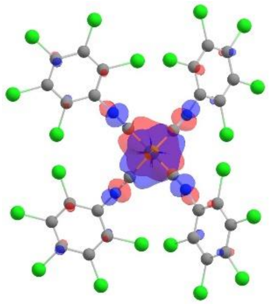<br>282	→ 92.7%	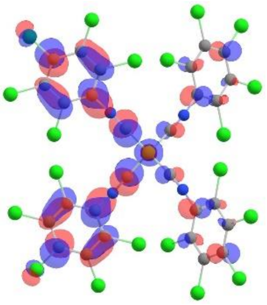<br>283	
S_4	2.7975 eV<br>443.20 nm	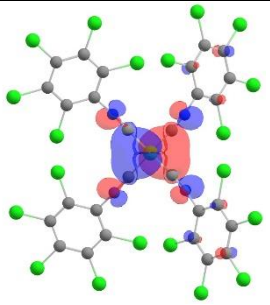<br>282	→ 94.6%	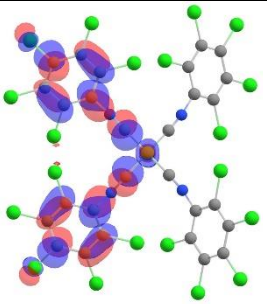<br>283	0.0086
S_5	2.8258 eV<br>438.76 nm	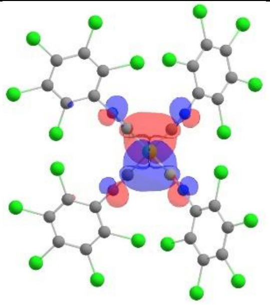<br>280	→ 5.6%	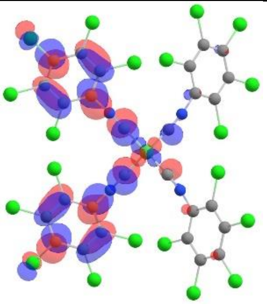<br>285	0.0039
		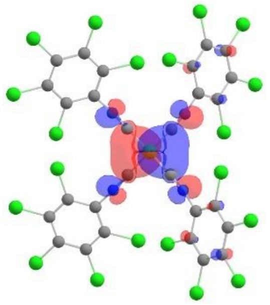<br>281	→ 27.5%	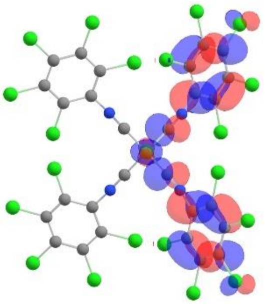<br>284	

		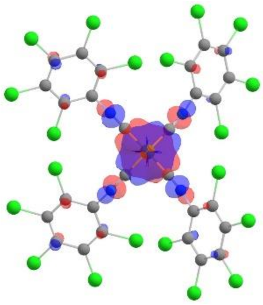<br>282	→ 64.2%	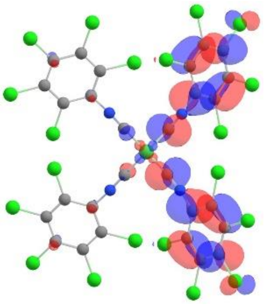<br>283	
S_6	2.8279 eV<br>438.43 nm	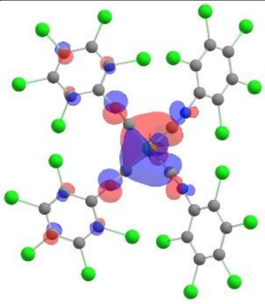<br>280	→ 3.4%	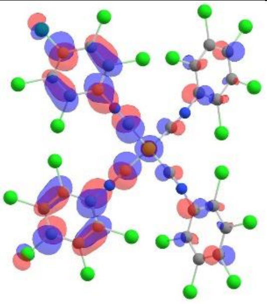<br>285	0.0664
		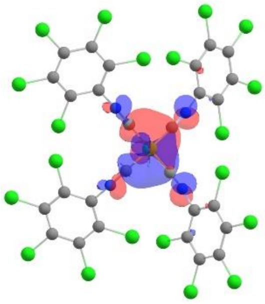<br>281	→ 41.8%	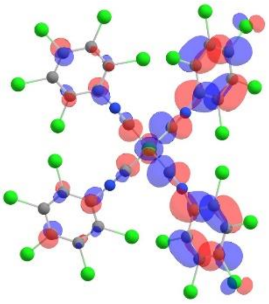<br>284	
		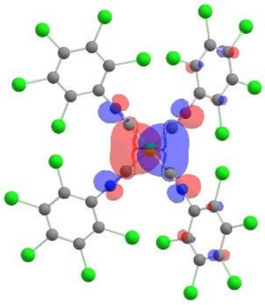<br>282	→ 50.9%	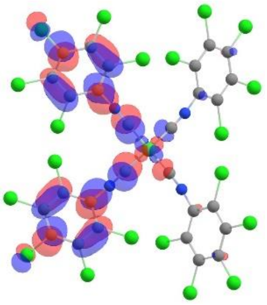<br>283	
S_7	2.8891 eV<br>429.15 nm	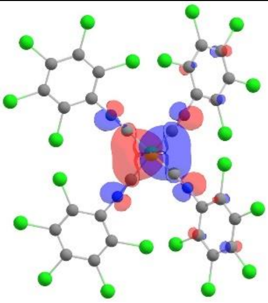<br>281	→ 10.1%	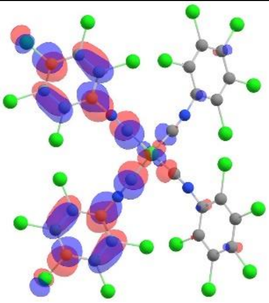<br>284	0.0087

		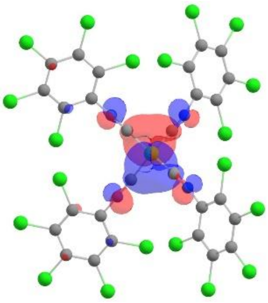 282	→ 85.3%	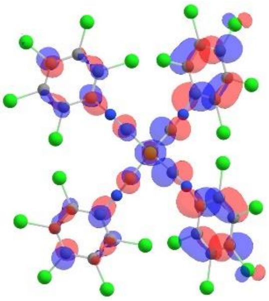 283	
S_8	2.9229 eV 424.18 nm	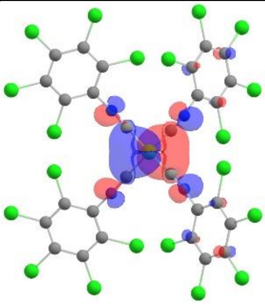 280	→ 26.9%	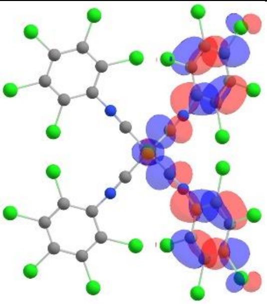 285	0.2957
		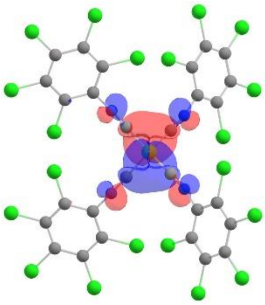 281	→ 30.4%	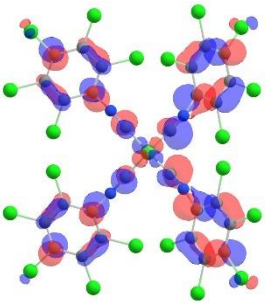 284	
		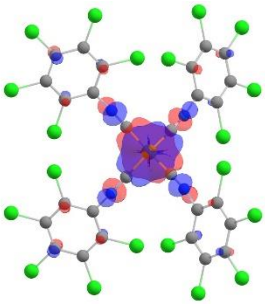 282	→ 37.0%	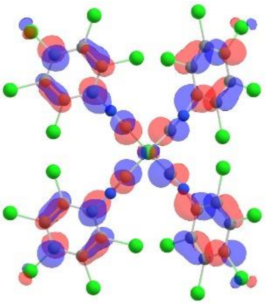 283	
S_9	2.9518 eV 420.03 nm	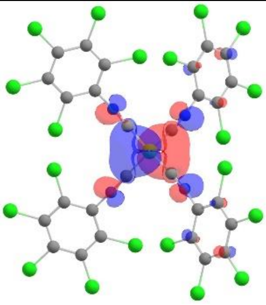 281	→ 8.6%	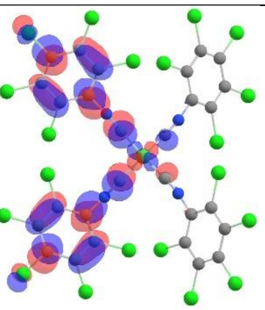 284	0.1438

		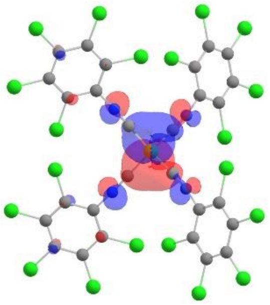<br>282	→ 86.2%	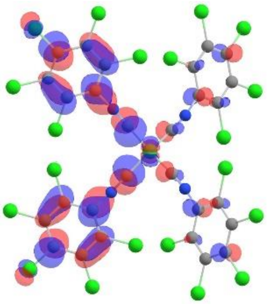<br>283	
S_{10}	3.0264 eV<br>409.67 nm	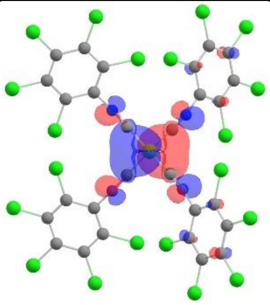<br>280	→ 3.8%	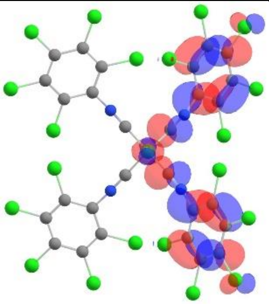<br>285	0.0051
		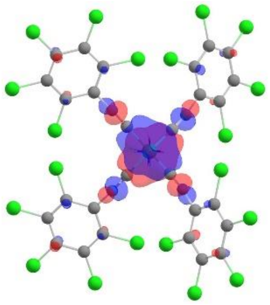<br>281	→ 5.1%	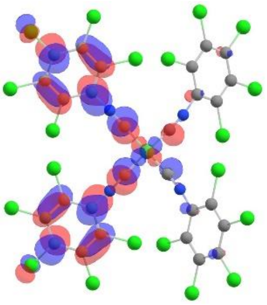<br>284	
		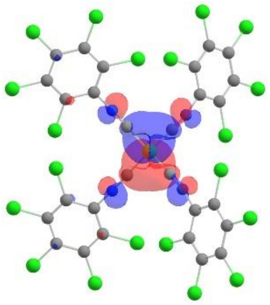<br>282	→ 87.7%	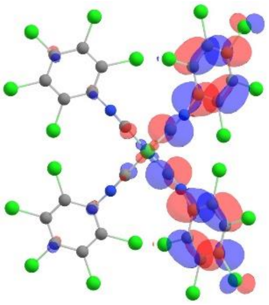<br>283	
S_{11}	3.0268 eV<br>409.62 nm	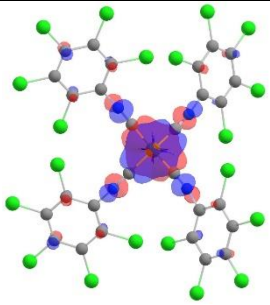<br>281	→ 10.7%	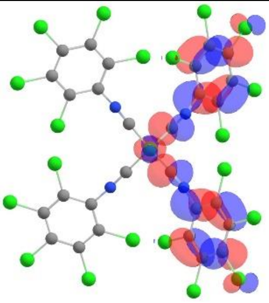<br>284	0.3765

		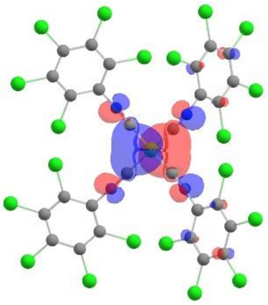 282	→ 83.1%	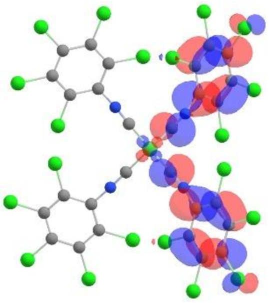 283	
S_{12}	3.1816 eV 389.69 nm	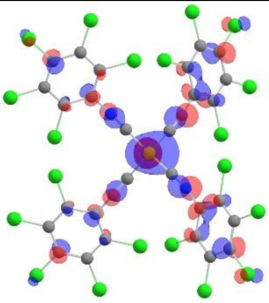 279	→ 5.3%	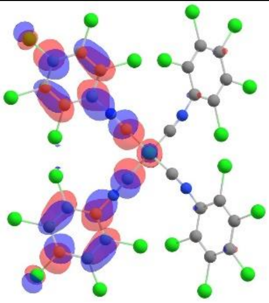 286	0.0053
		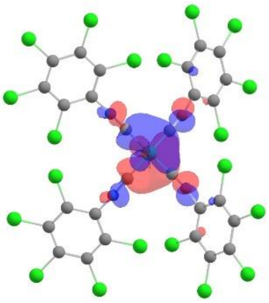 280	→ 12.5%	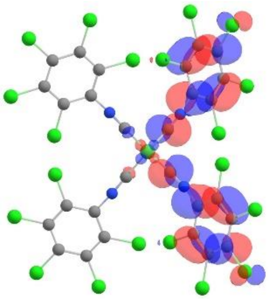 285	
		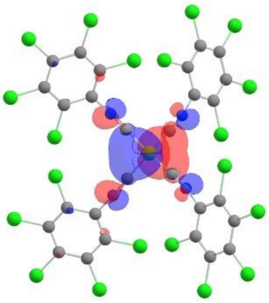 281	→ 32.6%	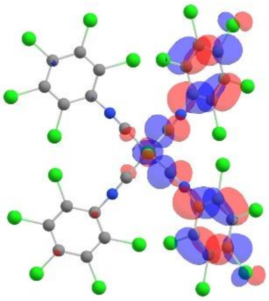 284	
		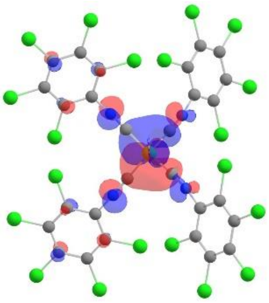 282	→ 41.0%	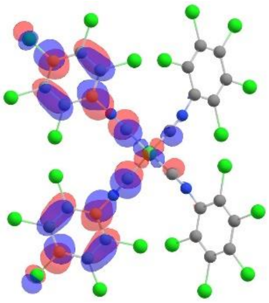 283	

8. Thermochemistry

The Gibbs energies of the complexation reactions in the gas-phase ($\Delta G_{rxn.g}$) were calculated using the reactions given in equations 1–4 on the B3LYP(D3BJ)/def2-TZVP level of theory.

$$Ni_{(g)} + 4\ CO_{(g)} \longrightarrow Ni(CO)_{4\ (g)} \quad \Delta G_{rxn,g} = -373.8\ kJ\ mol^{-1} \quad (eq.\ 1)$$

$$Ni_{(g)} + 4\ CN\text{–}C_6H_{5\ (g)} \longrightarrow Ni(CN\text{–}C_6H_5)_{4\ (g)} \quad \Delta G_{rxn,g} = -385.5\ kJ\ mol^{-1} \quad (eq.\ 2)$$

$$Ni_{(g)} + 4\ CN\text{–}C_6F_{5\ (g)} \longrightarrow Ni(CN\text{–}C_6F_5)_{4\ (g)} \quad \Delta G_{rxn,g} = -420.3\ kJ\ mol^{-1} \quad (eq.\ 3)$$

$$Ni_{(g)} + 4\ CN\text{–}C_6Cl_{5\ (g)} \longrightarrow Ni(CN\text{–}C_6Cl_5)_{4\ (g)} \quad \Delta G_{rxn,g} = -423.6\ kJ\ mol^{-1} \quad (eq.\ 4)$$

9. EDA-NOCV Analysis

Table S9: EDA-NOCV results of several $Cr(CO)_5L$ complexes from the $Cr(CO)_5$ (S) + L (S) → $Cr(CO)_5L$ (S) fragmentation. Energies given in kJ mol^{-1}. Calculated on the BP86(D3BJ)/def2-TZVPP//BP86(D3BJ)/TZ2P level of theory

**[Cr(CO)$_5$L]**	ΔE_{int}	ΔE_{Pauli}	ΔE_{elstat}	ΔE_{disp}	ΔE_{orb}	NOCV stabilization interaction	
						$\Delta E_{orb\ \sigma}$	$\Delta E_{orb\ \pi}$ (2x π-acceptor)
**CO**[a]	−211	446	−326	−21	−310	−138/**45%**	−162/**52%**
**CNC$_6$H$_5$**[a]	−227	486	−404	−27	−282	−152/**54%**	−118/**42%**
**CNC$_6$F$_5$**[a]	−229	495	−392	−28	−304	−152/**50%**	−140/**46%**
**CNC$_6$Cl$_5$**	−237	509	−403	−35	−310	−156/**50%**	−142/**46%**
**CNCH$_3$**[a]	−220	443	−383	−25	−255	−144/**56%**	−100/**39%**
**CNCF$_3$**[a]	−228	486	−376	−26	−312	−148/**47%**	−155/**49%**

[a] taken from ref.[3]

10. References

(1) Hahn, F. E.; Münder, M.; Fröhlich, R. Homoleptic Nickel(0) Phenyl Isocyanide Complexes. *Z. Naturforsch. B* **2004**, *59* (8), 850–854. DOI: 10.1515/znb-2004-0814.

(2) Slavov, C.; Hartmann, H.; Wachtveitl, J. Implementation and evaluation of data analysis strategies for time-resolved optical spectroscopy. *Anal. Chem.* **2015**, *87* (4), 2328–2336. DOI: 10.1021/ac504348h. Published Online: Feb. 3, 2015.

(3) Streit, T.-N.; Sievers, R.; Sellin, M.; Malischewski, M. Revisiting CNC_6F_5: The Quest for Isocyanide Ligands with Strong πAcceptor Properties Evaluated by Energy Decomposition Analysis. *ACS Omega* **2025**, *10* (31), 35095–35102. DOI: 10.1021/acsomega.5c04766. Published Online: Jul. 30, 2025.
